# Supplementary material for: Adolescent health and well-being check-up programme in three African cities (Y-Check): protocol for a multimethod, prospective, hybrid implementation-effectiveness study
Source: BMJ Open. 2024 Jun 21;14(6):e077533. doi: 10.1136/bmjopen-2023-077533 (PMC11331354; doi:10.1136/bmjopen-2023-077533)
Supplement: Author's manuscript [file bmjopen-2023-077533.draft_revisions.pdf]

BMJ Open

BMJ Open is committed to open peer review. As part of this commitment we make the peer review history of every article we publish publicly available.

When an article is published we post the peer reviewers' comments and the authors' responses online. We also post the versions of the paper that were used during peer review. These are the versions that the peer review comments apply to.

The versions of the paper that follow are the versions that were submitted during the peer review process. They are not the versions of record or the final published versions. They should not be cited or distributed as the published version of this manuscript.

BMJ Open is an open access journal and the full, final, typeset and author-corrected version of record of the manuscript is available on our site with no access controls, subscription charges or pay-per-view fees (http://bmjopen.bmj.com).

If you have any questions on BMJ Open's open peer review process please email info.bmjopen@bmj.com

BMJ Open

An adolescent health and wellbeing check-up programme in three African cities (Y-Check): protocol for a multimethod, prospective, hybrid implementation-effectiveness study

Journal:	*BMJ Open*
Manuscript ID	bmjopen-2023-077533
Article Type:	Protocol
Date Submitted by the Author:	07-Jul-2023

Complete List of Authors:	Banati, Prerna; World Health Organization,<br>Ross, David Anthony; Stellenbosch University<br>Weobong, Benedict; University of Ghana<br>Kapiga, Saidi ; National Institute for Medical Research Mwanza Research Centre; London School of Hygiene & Tropical Medicine<br>Weiss, Helen; London School of Hygiene & Tropical Medicine<br>Baltag , Valentina; World Health Organization<br>Nzvere, Farirai; London School of Hygiene & Tropical Medicine; Biomedical Research and Training Institute<br>Glozah, Franklin; University of Ghana<br>Nsanya, Mussa; National Institute for Medical Research Mwanza Research Centre<br>Greco, Giulia; London School of Hygiene & Tropical Medicine<br>Ferrand, Rashida; London School of Hygiene & Tropical Medicine, Department of Infectious Disease Epidemiology; Biomedical Research and Training Institute<br>Doyle, Aoife; London School of Hygiene & Tropical Medicine; Biomedical Research and Training Institute

Keywords:	Adolescent, PUBLIC HEALTH, Mass Screening, Schools

SCHOLARONE™
Manuscripts

An adolescent health and wellbeing check-up programme in three African cities (Y-Check): protocol for a multimethod, prospective, hybrid implementation-effectiveness study

Banati P, PhD MPH[1*], Ross DA, MA, BMBCh, MSc, PhD[2] Weobong B, MSc, PhD[3], Kapiga S, MD, MPH, ScD[4,5], Weiss HA PhD MSc[6], Baltag V MD PhD[1], Nzvere F[5,7], Glozah F, MPhil, PhD[3], Nsanya MK, MD, MSc[4], Greco G, PhD MSc[5], Ferrand RA, PhD MBBS[5,7], and Doyle AM, PhD[6,7] for the Y-Check Research Programme Team[8]

[1] Adolescent and Young Adult Health (AYH) Unit, Maternal, Newborn, Child and Adolescent Health and Ageing (MCA) Department, World Health Organization (WHO), Ave Appia 20, 1211 Geneva 27, Switzerland

[2] Institute for Life Course Health Research, Stellenbosch University, Tygerberg, South Africa

[3] University of Ghana School of Public Health, College of Health Sciences (UGSPH), Ghana

[4] Mwanza Intervention Trials Unit, National Institute for Medical Research Mwanza Research Centre (MITU/NIMR), Isamilo, Mwanza, United Republic of Tanzania

[5] Department of Infectious Disease Epidemiology, London School of Hygiene and Tropical Medicine, Keppel St, London WC1E 7HT, UK

[6] MRC International Statistics and Epidemiology Group, London School of Hygiene & Tropical Medicine, Keppel St, London WC1E 7HT, UK

[7] Biomedical Research and Training Institute (BRTI), 10 Seagrave Road, Avondale, Harare, Zimbabwe

[8] The Y-Check Research Programme Team in alphabetical order includes Prof. Philip B. Adongo, MA, PhD, Department of Social and Behavioural Sciences, School of Public Health, University of Ghana; Mr. Kenneth. S. Adde, MPhil, Department of Population and Health, University of Cape Coast; Prof Evans Agbeno, FGCS, MPH, MBCHB, Department of Obstetrics & Gynaecology, University of Cape Coast; Prof. Patricia Akweongo, MA, PhD, Department of Health Policy Planning and Management, School of Public Health, University of Ghana; Ms Tsitsi Bandason MSc, The Health Research Unit Zimbabwe/ Biomedical Research and Training Institute; Dr. Sarah Bernays, PhD MA, Global Health and Development, London School of Hygiene and Tropical Medicine and School of Public Health, University of Sydney; Dr Rudo Chingono PhD, The Health Research Unit Zimbabwe/ Biomedical Research and Training Institute Dr Chido Dziva Chikwari PhD, MSc, BSc, The Health Research Unit Zimbabwe/ Biomedical Research and Training Institute and MRC International Statistics & Epidemiology Group, Department of Infectious Disease Epidemiology, London School of Hygiene & Tropical Medicine, London, UK; Ms Ethel Dauya MPH, The Health Research Unit Zimbabwe/ Biomedical Research and Training Institute; Dr. S. Derry, PhD, Department of Bio-Statistics, School of Public Health, University of Ghana; Dr. Eric Koka, MPhil, PhD, Department of Sociology and Anthropology, University of Cape Coast; Dr. Constance Mackworth-Young, PhD MSc, Global Health and Development, London School of Hygiene and Tropical Medicine, Ms. Salome Manyau MSc, The Health Research Unit Zimbabwe/ Biomedical Research and Training Institute; Mr. Bernard A. Owusu, MPhil, Department of Population and Health, University of Cape Coast; Dr. Yovitha Sedekia, MPHDC, PhD, Mwanza Intervention Trials Unit (MITU)/National Institute for Medical Research (NIMR); Dr. Hannah Taylor-Abdulai, MPH, MPhil, PhD, Department of Physician Assistant Studies, University of Cape Coast; ; Dr. Mandi Tembo, PhD, The Health Research Unit Zimbabwe/ Biomedical Research and Training Institute and Department of Global Health and Development, Faculty of Public Health and Policy, London School of Hygiene and Tropical Medicine, London, United Kingdom.

* Corresponding Author

Email addresses

Banati P (banatip@who.int); Ross DA (dross.rbridge@gmail.com); Weobong B (bweobong@ug.edu.gh); Kapiga S (Saidi.Kapiga@lshtm.ac.uk); Weiss HA (Helen.Weiss@lshtm.ac.uk); Baltag V (baltagv@who.int); Nzvere F (Farrie.Nzvere@lshtm.ac.uk); Glozah F (fglozah@ug.edu.gh); Nsanya MK (mussa.nsanya@mitu.or.tz); Greco G (Giulia.Greco@lshtm.ac.uk); Ferrand RA (rashida.ferrand@lshtm.ac.uk); Doyle AM (aoife.doyle@lshtm.ac.uk)

For peer review only

Article Summary

Abstract

Background: During adolescence, behaviours are initiated that will have substantial positive or negative impacts on the individual's short- and long-term health and wellbeing, educational attainment and employment prospects. However, adolescents rarely have regular contact with health services, especially for health promotion and disease prevention, and services are not always appropriate for their needs. We co-developed with adolescents a health and wellbeing check-up programme, to improve adolescent health and wellbeing (Y-Check). This paper describes the methods to evaluate the feasibility, acceptability, short-term effects, and cost-effectiveness of Y-Check in three African cities: Cape Coast in Ghana, Mwanza in Tanzania and Chitungwiza in Zimbabwe.

Method: This is a multi-country prospective intervention study, with a mixed-method process evaluation, to assess the implementation, effects and short-term cost-effectiveness of Y-Check. The intervention involves screening, on-the-spot care and, if needed, referral of adolescents through health and wellbeing check-up visits in early adolescence (10-14 years) and older adolescence (15-19 years old). In each city, the intervention will be delivered to 2000 adolescents recruited in schools (both age groups) or community venues (older adolescents only). The adolescents will be followed-up at 4 months (all three cities) and 12 months (Zimbabwe only). The study will assess the effects of Y-Check on knowledge and behaviours, as well as clinical outcomes and costs. The primary outcome will be the proportion of those screening positive for at least one condition who receive appropriate on-the-spot care or complete appropriate referral for all identified conditions within four months. Secondary outcomes include yield of untreated conditions, reported health-related risk and protective behaviours, engagement with health services, wellbeing, clinical and educational outcomes. A process evaluation will investigate acceptability, feasibility, uptake, and fidelity, and an economic evaluation will explore cost effectiveness.

For peer review only

Discussion: This study is innovative in evaluating a comprehensive adolescent health and wellbeing check-up intervention which addresses both health conditions that impact on wellbeing during adolescence, and risk factors for future ill-health or lack of wellbeing in three African cities. Evidence of the intervention´s feasibility, acceptability, and short-term positive effects and costs will support larger scale intervention implementation and rigorous, longer-term evaluation.

Keywords: Adolescent, health, wellbeing, check-ups, screening, implementation research, effectiveness, cost-effectiveness

Trial registration: NCT06090006

For peer review only

Additional information

Strengths and limitations of the methodology:

- **Strength**: This study will utilize existing health care infrastructure in low- and middle-income country settings, assessing real world implementation situations and therefore it will be relatively straightforward to directly apply the findings to programs.
- **Strength**: This is a relatively large study of 6000 adolescents in 3 countries. The study takes the views of young people centrally into the design of the intervention.
- **Limitation**: Although the primary outcome is an implementation science / programmatic outcome, the effectiveness data is based on pre-post comparison.
- **Limitation**: This study will have limited ability to assess sustainability of effects over the longer term as the follow up period is 4 months
- **Limitation**: This study is operating in three African cities which may limit generalizability to rural areas.

Authors' contributions: DR, AD, PB conceived and drafted the paper. All other authors contributed to writing.

Funding statement: This work was supported by the Botnar Foundation grant number (RG21-001) and UKRI (MR/T043156/1)

Competing interests: The authors declare no competing interests.

Ethics and Dissemination: This study has received approval from the World Health Organization (WHO/ERC Protocol ID Number ERC.0003778); Ghana Health Service (Protocol ID number GHS-ERC: 027/07/22), the United Republic of Tanzania National Institute for Medical Research (Clearance No. NIMR/HQ/R.8a/Vol.IX/4199), the Medical Research Council of Zimbabwe (Approval number MRCZ/A/2766), and the London School of Hygiene and Tropical Medicine (Approval numbers 26395 and 28312). Issues of consent and disclosure are addressed in the paper.

Manuscript Wordcount: 5023

For peer review only

Background

To unlock human potential and accelerate progress towards achieving the Sustainable Development Goals (SDGs), it is essential to improve the health and wellbeing of adolescents (10-19 years) (Bundy et al., 2018). Health is an essential component of human capital (World Bank, 2019), yet adolescent investments have focused primarily on either health or education services with little attention to synergies between these (Tomlinson et al, 2019). Research investments in the first 1000 days of life have dramatically outweighed investments in the subsequent 7000 days, leaving an evidence gap on how to develop and sustain human potential through adolescence and early adulthood (WHO, 2017).

Among adolescents in low- and middle-income countries (LMICs), HIV/AIDS, road injury, diarrheal diseases, self-harm, iron-deficiency anemia and skin diseases are among the top causes of morbidity and mortality (WHO 2023; WHO, 2019; Kuper et al., 2014). Identifying adolescents with poor health, health-compromising behaviours or undiagnosed disability is important given (1) the growing number of adolescents and their low frequency of regular contacts with health services (Kruk et al., 2022) (2) the high proportion of the total global burden of disease that occurs in adolescence and (3) the fact that many key health conditions (e.g. mental health disorders) and behaviours (e.g. tobacco and alcohol use, unhealthy diet, low physical activity, risky sexual behaviours) that predispose to preventable serious conditions in later life start in adolescence (4) the negative impact of poor health on educational attainment and employability and other transitions to healthy adulthood, and (5) gender-related vulnerabilities, including violence, abuse, unintentional injury, sexual and reproductive health (SRH) and gendered mental health outcomes which may emerge or be exacerbated during this period of life, setting negative trajectories to lifetime and intergenerational health and wellbeing (WHO, 2017).

Systematic reviews have identified individual interventions that are effective at improving various aspects of adolescent health and/or wellbeing (WHO, 2017.) However, most adolescents only come into contact

For peer review only

with health services when they are ill, and services are not always appropriate for their needs (WHO, 2015). This represents a missed opportunity for early detection of health problems, for health promotion, and for the development of health-seeking behaviours. Early and sustained engagement with health and social services could reap a triple dividend for human development by improving the health and wellbeing of adolescents, their health and wellbeing in adulthood and the health and wellbeing of their future offspring (World Bank 2019; WHO 2017; Patton et al., 2016.)

Routine health and wellbeing check-up visits for adolescents that screen for multiple conditions and risk behaviours could provide an entry point into services and be highly cost-effective (Sanci 2011; Harris et al., 2017). Obtaining evidence on the optimum content, delivery, effectiveness and cost of check-ups is a high priority for adolescent health research so that governments can be informed by the evidence on how to initiate or strengthen existing health and wellbeing check-ups during adolescence (Nagata et al., 2018). Many high-income countries have national recommendations related to adolescent health check-ups, which have been largely based on expert opinion (Hagan et al., 2008; Hagan et al., 2011; Royal Australasian College of General Practitioners, 2012). In LMICs, if provided at all, preventive and promotive health services for adolescents are largely provided in schools and are usually limited to deworming and vaccination campaigns. They do not usually address other key conditions and risk factors such as nutrition, mental health, SRH or disability (WHO, 2021a; Baltag and Moran, 2018). If a system-wide approach to check-ups exists in adolescence, in LMICs it is often limited to a screening activity without other components such as brief intervention or anticipatory guidance (Baltag and Moran, 2018).

This paper describes the protocol for the Y-Check: Evaluating the effects of adolescent health check-ups study, a prospective hybrid implementation-effectiveness study evaluating the feasibility, acceptability, short-term effects, costs and cost-effectiveness of the Y-Check intervention in three African cities. This study has received approval from the World Health Organization (WHO/ERC Protocol ID Number ERC.0003778); Ghana Health Service (Protocol ID number GHS-ERC: 027/07/22), the United Republic of

For peer review only

Tanzania National Institute for Medical Research (Clearance No. NIMR/HQ/R.8a/Vol.IX/4199), the Medical Research Council of Zimbabwe (Approval number MRCZ/A/2766), and the London School of Hygiene and Tropical Medicine (Approval numbers 26395 and 28312) .

The Y-Check intervention

Y-Check is a novel intervention delivering a health and wellbeing check-up and where indicated will provide on-the-spot care and/or referral for common conditions on two occasions in adolescence (in young adolescents (10-14 year-olds) – soon after the onset of puberty - and in older adolescents (15-19 year-olds) – when many adolescents become, or are soon to become, sexually active). It will also provide health promotion information and materials to support positive behaviours and healthy lifestyles during adolescence and beyond. The intention is that in the context of a future routinely-delivered programme, every adolescent will have two guaranteed contacts with the health care system. Adolescents will only be screened for conditions that have an accurate, low-cost, acceptable screening test and a locally accessible, effective intervention. The conditions selected for screening will be chosen to reflect the local epidemiological contexts (e.g. screening for malaria will only take place in malaria endemic areas). Respecting specific requests from the Ministries of Education in all three cities, the study will only include sexual and reproductive health screening and services at the community sites (which only include older adolescents).

Figures 1 and 2 present the Theory of Change and description of the intervention. Table 1 applies the TIDieR checklist (Hoffman et al, 2014) to describe details of the intervention.

Locally accessible services will be identified and assessed in terms of their ability to provide the services recommended by local and WHO guidelines, willingness to accept referred adolescents, and the fees charged to the project will be negotiated by the research team for services provided to referred

For peer review only

adolescents (where adequate services are not covered by national health insurance schemes, free NGO services or free public health care).

The intervention was designed following formative research conducted in three African countries between 2019 and 2020 (Chingono, Mackworth-Young et al. 2021; Weobong et al. (in preparation); Sedekia et al (in press)). This formative research revealed that the proposed adolescent health and wellbeing check-ups are likely to be feasible to implement and acceptable to stakeholders in Ghana, Tanzania and Zimbabwe, and are likely to meet the perceived needs of key stakeholders including adolescents, their parents, and key policy makers in the health and education sectors (WHO, 2020). Further, we showed that the programme is likely to produce a substantial yield of important, previously untreated, treatable conditions. Human-centered design techniques were used alongside desk review to define elements of objective and subjective importance to the health and wellbeing of adolescents, identify facilitators and barriers to adolescent health seeking, preferences for delivery of routine health check-ups, and potential effects of interventions to select the content and method of delivery of the Y-Check intervention. Interviews and participatory workshops with adolescents, parents of adolescents and key stakeholders from the ministries of health and education, non-governmental organizations, healthcare workers and teachers found that there was overall support for the introduction of routine health check-ups (Chingono, et al 2021; Weobong et al., in preparation). To navigate potential barriers, stakeholders suggested clear messaging, awareness building, and sensitization campaigns to overcome disinterest in preventative healthcare and, in some contexts, mitigate cultural or religious messaging against healthcare engagement (Chingono, et al 2021).

Insert Figures 1 and 2

For peer review only

Table 1: Template for Intervention Description and Replication (TIDieR) checklist describing the Y-Check intervention

Item	Item
**Brief name**	
1	Evaluating the effectiveness of adolescent health check-ups (Y-Check)
**Why?**	
2	Identifying adolescents with poor health, health-compromising behaviours or undiagnosed disability is important for their health and wellbeing, and also for communities and nations<br>Most adolescents only come into contact with health services when they are ill, and services are not always appropriate for their needs<br>Routine health and wellbeing check-up visits for adolescents that screen for multiple preventable and/or treatable conditions and risk behaviours could provide an entry point into services and be highly cost-effective
**What?**	
3	The intervention includes a comprehensive health check-up for priority conditions customized to national and local contexts.<br>Where indicated, Y-Check will provide on-the-spot care and cover all clinical costs associated with referrals to further care provided by the public health system or non-governmental organizations (NGOs).<br>During the check-up, adolescents will receive health promotion information and limited supplies of key health commodities.<br>Clinical costs of services are covered by the study if accessed within 4 months of the check-up.
4	Adolescent-friendly services will be provided, as defined by WHO (2018). Nationally-approved protocols will be applied. Adolescent privacy and confidentiality will be protected.
**Who provided?**	
5	Y-Check teams will be staffed with health professionals trained to provide quality adolescent-friendly health services in line with nationally-approved protocols. Y-Check teams will also be trained in the use of the digital application which will be used for data collection. Public and private not-for-profit care facilities providing referrals will meet national accreditation guidelines.
**How?**	
6	The Y-Check service will take place over a 60-90 minute period face-to-face. Any referrals will only be subsidized by the study if they take place within 4 months.
**Where?**	
7	The Y-Check service will be provided in schools and community venues, in outdoor tents where required.<br>Referrals will be to public or private not-for-profit providers as close as possible to the adolescent´s home. Providers will be vetted by the study team as being able to provide the necessary referral services to national and WHO-recommended standards.
**When and How Much?**	
8	Within the current phase of the study, each adolescent will receive Y-Check once. Within a routine programme the intention would be that the intervention will be delivered twice during adolescence, once when the adolescent is 10-14 years old, and a second time when they are 15-19 years old.
**Tailoring**	
9	The content of the intervention is tailored to local context. The exact set of conditions that will be assessed as part of Y-Check will be adapted based on burden of disease, and availability of local tests and referral services.

Item	Item
**Modifications**	
10	Any modifications will be reported in the article reporting the results of the study.
**How well?**	
11	Intervention fidelity (adherence, integrity, quality) will be evaluated through a process evaluation including youth-friendly health services quality.
12	Intervention fidelity will be reported in the article reporting the results of the study.

For peer review only

Methods/Design

Aims

The aim of the study is to develop and implement in three African cities a potentially sustainable adolescent health check-up programme, and evaluate the acceptability, feasibility, short-term effects, and cost-effectiveness of the programme to improve health and wellbeing.

Objectives

(1) To develop and pilot test a check-up programme for adolescents that screens for important preventable and treatable health conditions using accurate and acceptable screening tests and provides locally accessible effective interventions.

(2) Through a prospective intervention study in selected schools and communities to:

- Estimate short-term impacts on adolescent health and wellbeing outcomes: clinical outcomes, health-related knowledge and behaviours, intentions, agency, and perceived social support for behaviour change; engagement with health services.
- Understand, through process evaluation, the feasibility and fidelity of implementation, the acceptability and uptake, and the influence of context.

- Estimate the cost-effectiveness of the programme in reducing overall disease burden and improving adolescent wellbeing

(3) Obtain information on key parameters needed for the planning of an evaluation study: prevalence of health conditions and behaviours, acceptability of referral, feasibility of following-up programme participants and delivering quality follow-up care, initial estimates of the impact of the programme on longer-term health, educational and wellbeing outcomes based on the short-term implementation and effectiveness outcomes observed in this phase of the research programme, and factors related to the optimal implementation of the Y-Check intervention.

(4) To refine the programme and its theory of change, and finalise optimal methods for the measurement of the impact of the programme in future studies.

Theory of Change

We hypothesise that a routine health and wellbeing check-up visit for adolescents that screens for multiple conditions and risk behaviours will have an immediate and long-term positive impact on health and wellbeing outcomes (Figure 1).

Health seeking and promotion behaviours among adolescents operate in complex environments and across ecological levels (Patton et al., 2016), with determinants at individual, interpersonal institutional/organizational, community and public policy levels. Drawing from the health promotion literature (Green and Kreuter, 1999; McLeroy 1988), the Theory of Change for Y-Check (Figure 1) draws on thinking that recognizes pre-disposing, enabling and reinforcing factors as capacities to be strengthened in order to achieve adolescent wellbeing at the individual level; that responsive parenting can support adolescents to meet their own health and wellbeing goals; that systems-based approaches (including stronger linkages between health and education systems) can improve outcomes for adolescents, especially reaching the most vulnerable and those in need; and that an enabling environment

For peer review only

(especially in schools and communities) can support adolescents to take action towards improving their health.

Study setting

Our study will be undertaken in three African cities: Cape Coast in Ghana, Mwanza in Tanzania and Chitungwiza in Zimbabwe. These cities are described in Table 1.

Table 1: The study cities, schools and communities

Cape Coast, Ghana	Mwanza, Tanzania	Chitungwiza, Zimbabwe
Cape Coast Metropolis is located on the coast of Ghana, 150kms west of the capital city, Accra. It has a population of 169,894 with three-quarters of the households residing in urban areas.<br><br>Literacy in 11-24 year-olds is about 97%. In 2016, 11,233 (68.8%) of 12-14 year-olds were enrolled in junior high schools while 8,407 (91.6%) of 15-17 year-olds were enrolled in senior high schools. For Ghana as a whole, primary and secondary net enrollment rates in 2019 were 86% and 57%, respectively (UNESCO, 2023)<br><br>There are 36 health facilities (26 public and 10 private) in the metropolitan area, including a regional hospital that serves as a secondary referral facility.<br><br>The study will be conducted in 8 schools and local community venues in four communities that include two relatively affluent communities with trading being the main source of livelihood and two relatively poorer communities where fishing and farming dominate, respectively.	Mwanza is located on the southern shores of Lake Victoria in North-Western Tanzania and is the second largest city in Tanzania with a population of over 900,000 and an annual growth rate of 3% (Tanzania National Bureau of Statistics, 2016). Economic activities in Mwanza include fishing and fish processing, subsistence agriculture and support services to nearby gold and diamond mines.<br><br>Adolescents make up 24.2% of the population of the city (Tanzania National Bureau of Statistics, 2016). As of 2020/21, the primary and secondary school net enrollment rates were 82% and 39%, respectively (Tanzania National Bureau of Statistics, 2022)<br><br>Available public health services include 26 dispensaries, 5 health centres, 2 district hospitals, 1 regional hospital and 1 tertiary/teaching hospital (Tanzania National Bureau of Statistics, 2016; Ilemela Municipal Council, 2017).<br><br>The study will be conducted in 4–6 purposively-selected communities and in up to 8 primary schools and 8 secondary schools within the catchment area of health facilities serving the selected communities in the two districts within Mwanza city.	Chitungwiza is the third largest city in Zimbabwe, located approximately 25km south of the capital city, Harare. It has a population of about 456,000. The houses are mostly high-density, single-story, detached units with small yards that are generally used for growing vegetables. Most of the people work in Harare, as there is little industry in Chitungwiza itself.<br><br>Zimbabwe has a school-going population (8-18 years) of approximately 4.3 million (Ministry of Primary and Secondary Education Zimbabwe, 2022). Net primary enrollment rate across Zimbabwe is 94%; net secondary enrollment rate is 54% (ZIMSTAT, 2021)<br><br>In Chitungwiza, there is one tertiary hospital, 4 public primary healthcare facilities, 20 private medical facilities, 30 government primary schools, and 13 government secondary schools (all mixed sex).<br><br>The study will be conducted in four distinct communities which are representative of the urban, peri-urban and rural populations of Chitungwiza. Eligible schools must have a student population of at least 200 learners in Grade 6 or at least 75 learners in Form 5; and be located in or close to one of the selected study communities.

Study design

In this prospective hybrid implementation-effectiveness study, 2000 adolescents per city who receive the Y-Check intervention will be followed up at 4-months, and at 12-months (Zimbabwe only).

Stakeholder engagement

In each city, the research study is undertaken in partnership with both the national and municipal Ministries of Health and Education. Each country has a policy framework that provides encouragement for the introduction of health and nutrition education and promotion among adolescents, including screening for communicable and non-communicable diseases, immunization, growth monitoring and assessments and nutritional services (Government of Zimbabwe 2018; Ghana Health Service, 2016; Government of Tanzania, 2021).

This study will build on stakeholder engagement, the process for which was established in each research setting during the formative phase. In each city, a Community Advisory Committee (CAC) comprising key community leaders and stakeholders will be reinforced or set up to facilitate input from, and feedback to, participating communities and a Youth Advisory Group (YAG) will provide a forum for adolescents to input into the programme. The YAG will meet with research staff at least 4 times per year, be active participants in programme design and dissemination workshops, and help to ensure that the programme meets the needs of adolescents. Community engagement will be an ongoing process through regular contacts with the CAC, the YAG and other stakeholders, such as teachers, health workers, Community Based Organizations (CBOs), Non-Governmental Organizations (NGOs), and religious leaders. In addition, a key aspect for building confidence within communities is the knowledge that the study has the support of the government.

For peer review only

Intervention development and pilot testing

Prior to implementation, preparatory activities will include community engagement, participatory co-design, negotiating referral arrangements and pre-testing of screening tools, procedures and referral protocols. Pilot studies in each setting will provide initial estimates of the frequency of health and behavioural outcomes, and help to refine the intervention model.

Pilot testing will involve the implementation of the screening tools and procedures with approximately 200 adolescents in each of the three cities with revisions and repeat pilot testing where required. There will be an opportunity for young people and stakeholders to suggest additional client-centered outcomes that may reflect some of their priority concerns or intentions that should be captured.

Intervention implementation

The intervention will be delivered over a period of 2-6 months in each of the settings. The follow-up visits will take place at the same school or community setting as the initial check-up. In addition to covering all clinical costs, the equivalent of USD 5 will be given to each participant who attends the follow-up to cover any transport costs that they might have incurred. Additionally, health and hygiene related items will also be provided for adolescents to take home, including tooth cleaning kit (toothbrush and toothpaste), fruit, bottle of water, two pairs of underpants, pack of reusable sanitary pads (girls only)

Composition and training of Y-Check team

The Y-Check team will be trained to deliver adolescent-responsive and age-appropriate services according to national and WHO guidelines, recognizing also the needs for privacy and confidentiality (WHO, 2015). This includes providing services that are attractive to adolescents, meet their needs comfortably and responsively, and that are attentive to their privacy. These principles and approaches will be embedded into each part of the Y-Check intervention. Visual and auditory privacy will be prioritized, through the use of separate tents, rooms or screens. Health workers will employ standard gowning and draping for clinical procedures.

For infection prevention and control (IPC), all study procedures including interviews, physical examinations and blood tests will take place in well-aerated tents or outdoors, and will follow relevant nationally-approved protocols for all staff and participants.

For peer review only

The Y-Check team will be trained in good clinical practice, data protection and confidentiality, and clinical staff will be trained in counselling for participants testing positive for any of the conditions being screened for within Y-Check as well as in general counselling skills.

Inclusion and exclusion criteria

To be included in the study, adolescents aged 10-19 years must fall into one of the first three categories below and fulfil category 4.

1) Be attending selected classes of Year 5 of primary school in Mwanza (median age 11 years); Grade 5/6 of primary school in Chitungwiza (median age 11 years); or Year 1 of Junior Secondary School in Cape Coast (median age 12 years) OR

2) Be attending selected classes in Year 3 of Secondary School in Mwanza (median age 17 years), Form 3/4 in Chitungwiza (median age 17 years), or Year 2 of Senior Secondary School in Cape Coast (median age 16 years) OR

3) Be resident in a selected community during the time of the Y-Check intervention, and be aged 16-19 years

AND

4) Have a completed and signed Informed Consent form, or a signed Informed Assent Form and signed Parental/Guardian Informed Consent Form if the adolescent is seen in the community and is below the national age of consent or is seen in a school, irrespective of their age.

Consent and Assent procedures

For peer review only

Before the visit of the implementation team, information on the Y-Check programme will be distributed to parents/guardians through the schools and to community members through an active communication campaign in collaboration with the CAC and the YAG. School and community meetings will allow parents and community members to ask questions about the programme and give their feedback.

In schools, adolescents will have a short introductory meeting with a member of the Y-Check team typically in a class or group setting. Parents meetings will then be held in each of the schools, to which all the parents and guardians of eligible learners will be invited. During these sessions, information will be provided about the study, its objectives and procedures, possible risks and procedures that will be used to maintain confidentiality. These meetings will provide an opportunity for the adolescents, parents and guardians of eligible adolescents to learn more about the Y-Check intervention and the research linked to it and to have their questions answered.

No participants will be screened, receive care or be counselled or interviewed without their informed consent (community participants who are above the national age of consent), or, for minors, their assent and parental consent, unless they are determined to be emancipated minors (WHO, 2021b). Following advice from Ministries of Education in all three countries, all adolescents seen in schools will be considered to be minors and require parental consent, irrespective of their age.

Minor adolescents' assent will be ascertained and documented in an assent form. Parents or guardians who would like their adolescent to receive the check-up will be asked to provide their written consent. On the day of the check-up visit, a verbal confirmation of their previous written assent will be requested from the adolescent. In Ghana and Tanzania, where the minimum age for providing consent to medical and health-related research is 18 years, clients of all ages under 18 will provide completed parental consent forms and provide written assent before proceeding through the check-up visit regardless of whether the check-up is in schools or communities. In Zimbabwe, a waiver of parental consent has been

For peer review only

given by the Medical Research Council of Zimbabwe (MRC-Zimbabwe) so that participants aged 16 and 17 years who attend the check-ups in the community venues will be allowed to provide written consent for themselves.

The intervention will be conducted in private and not in the presence of the parent or guardian. Contact details of the study team will be shared with participants in case they have questions at a later stage. All participants will be reminded that participation is entirely voluntary and will be told that they can opt out of the research or services at any time.

Data collection

During the Y-Check intervention and follow up

Data collection during baseline and follow-up visits will include self-completed evaluation questionnaires, self-reported screening tool responses and screening visit consultations, measurements and specimen collection and an exit interview. Data on the implementation process and on adolescent outcomes will be collected in digital and paper-based formats. A user-friendly digital data collection app for the check-ups will be developed and housed on a tablet computer for direct use by the adolescent. Initial sections will include audio-assisted, user-friendly self-completion questions for adolescents to fill out. This will utilize engaging content and processes, tailored to adolescents´ interests. The option of a face-to-face interview will also be available if the adolescent is unable to use the tablet or has low literacy level. Health services registers and school registers will also be reviewed to determine the number of adolescents of the relevant age ranges, and school attendance by the classes involved in Y-Check. To help build the referral process, existing adolescent services will be mapped in the study communities.

Process evaluation

For peer review only - http://bmjopen.bmj.com/site/about/guidelines.xhtml

The process evaluation is guided by the UK MRC's Process Evaluation framework to understand intervention implementation (including feasibility and fidelity), mechanisms of impact (including acceptability and uptake), and the influence of context (Moore et al., 2015). Key implementation outcomes of interest are acceptability, adoption, appropriateness, feasibility, and fidelity. Data on contextual factors and barriers and facilitators to programme implementation will be gathered using routinely-collected programme monitoring data. Qualitative data will be collected through 1) observations of the Y-Check intervention and referrals, as well as team meetings; 2) in-depth interviews with eligible adolescents who received, adolescents who were referred, and adolescents who did not receive Y-Check, as well as with school authorities and the Y-Check service providers; and 3) participatory workshops with teachers, adolescents, and parents. Quantitative programme monitoring data will be collected routinely within the Y-Check visit, including through a participant exit interview. Process evaluation data will be analysed iteratively and thematically, through regular analytical discussions and analytical memos to draw out the main themes emerging from the data. Across the pilot and intervention studies, data collection for the process evaluation will include real-time feedback to the implementation team.

Economic evaluation

A costing study will be conducted to estimate the total costs of developing, setting up, and running the Y-Check package, in school and community settings. A combination of top-down and ingredients-based costing approaches will be used to generate cost estimates for the whole package, and for each component/activity. All costs will be estimated from the perspectives of the adolescents, the schools/community and implementing partners/service providers. Financial and economic costs will be calculated for all inputs. These inputs will be identified and measured using process data, staff interviews and observations, document review, and accounting records.

For peer review only

Costs will be inputted and analysed in an Excel-based costing tool. The cost analysis will describe the distribution of costs across different forms of inputs, and will estimate the unit cost per adolescent reached, screened, and treated on the spot or referred; cost per unit of measure for selected process and effect outcomes such as cost per condition detected, cost per condition appropriately treated on-the-spot or with a completed referral within 4 months, cost for a unit improvement in reported quality of life and Disability Adjusted Life Years (DALYs) averted.

The cost and cost-effectiveness estimates will be compared to other programmes in the region (eg. human papillomavirus vaccination, deworming) and will inform programme replication, scalability, and financial sustainability.

Data protections

Data protection will be strictly observed. After study completion, data will be stored in the LSHTM-curated digital repository 'Data Compass' following General Data Protection Regulation (GDPR) guidelines. Data and code registered in LSHTM Data Compass will be made open access following deposit. A Data Safety and Monitoring Board (DSMB) has been constituted to assist in managing adverse events, though we expect these to be very rare since all treatment and care are standard with no novel treatments.

Study outcomes

Outcomes will be ascertained during the check-up screening visit and through collection of referral vouchers from the referral health facilities, and, for outcomes related to health and wellbeing impacts, through data from the 4-month and, in Zimbabwe only, 12-month follow-up visits. Outcomes related to completed referrals will be triangulated against participants´ self-reports at the 4-month and. In Zimbabwe only, 12-month follow-up visits. Review of school and health service registers will be used to see whether attendance has increased during the period when Y-Check is being implemented.

For peer review only

The primary outcome will be the proportion of those screening positive for at least one condition who receive appropriate on-the-spot care or complete appropriate referral for all identified conditions within 4 months. This will be measured using data collected at the initial check-up visit and through recovery of referral vouchers given to participants to allow them to access referral services for free during the 4-months after the Y-Check screening. Completed referral is defined as attending at least the first referral appointment.

Secondary implementation outcomes will include the proportion of those screening positive for each condition who receive appropriate on-the-spot care or complete appropriate referral for that condition within 4 months, the yield of previously untreated conditions, clinical outcomes at 4 months among those who had originally screened positive for each condition, and intervention acceptability, adoption, appropriateness, feasibility, fidelity and cost. Secondary effectiveness outcomes will include knowledge about health services and health behaviours, self-reported agency and self-efficacy to make decisions about their health, self-reported health-related risk and protective behaviours, reported engagement with health services, wellbeing, self-esteem and quality of life, clinical outcomes, and educational outcomes, which will be collected within the Y-Check and follow-up visits. The short-term cost-effectiveness of the intervention will be estimated (calculated by a comparison of the costs of the intervention against the primary and secondary outcomes and including short-term changes in self-reported quality of life). All outcomes for the study are described in Table 2.

Sample size

In each city, the intervention will be implemented for 10-14 year-olds in up to 6 government primary schools (N=500 for young adolescent girls, and N=500 for young adolescent boys), and for 15-19 year-olds in up to 8 secondary schools and up to 3 community venues (N=500 for older adolescent girls, and N=500 for older adolescent boys), giving a total sample size of 2,000 adolescents (10-19y).

For peer review only

The sample size provides specified precision around the primary outcome. For example, for the primary outcome, within each age group and gender, if 150 (30%) of 500 participants screen positive for at least one condition, and 75% of those who screen positive are correctly managed (n=112), the 95% CI for correct management will be +/- 7%.

Table 2: Study outcomes and means of verification

Outcome	Sources of data
**Primary outcome**	
Proportion of those screening positive for at least one condition who receive appropriate on-the-spot care or complete appropriate referral for all identified conditions within 4 months (i.e. they attend a provider for referral care who has been accredited by the study team and has been shown to be capable of providing appropriate referral care).	• Programme monitoring data including records of attendance for referrals<br>• Screening tool (self-reported symptoms or conditions, measurements and clinical actions)
**Secondary outcomes**	
**Implementation outcomes**	
Proportion of those screening positive for each condition who receive appropriate on-the-spot care or complete appropriate referral for that condition within 4 months.	• Programme monitoring data including records of attendance for referrals<br>• Screening tool (self-reported symptoms or conditions, measurements, and clinical actions)
The yield of previously untreated conditions.	• Programme monitoring data including records of attendance for referrals<br>• Screening tool (self-reported symptoms or conditions, measurements, and clinical actions)
Intervention acceptability (satisfaction): acceptability to adolescents and to other stakeholders (eg. schools, parents, health workers).<br>Intervention adoption (uptake, utilization): Y-Check uptake, referrals completed.<br>Intervention appropriateness (perceived fit, perceived relevance, perceived usefulness): perceived value of the intervention to adolescents and to other stakeholders.<br>Intervention feasibility (actual fit, practicability): Y-Check visits completed, referrals completed, stakeholder support (including community).	• Programme monitoring data including records of attendance for referrals<br>• Screening tool (self-reported symptoms or conditions, measurements, and clinical actions)<br>• Self-completed evaluation questionnaire<br>• Exit interviews<br>• Observations of the Y-Check visits and of selected referrals<br>• Interviews and workshops with adolescents, healthcare providers, community members, teachers, parents and key stakeholders
Intervention fidelity (adherence, integrity, quality): completeness of training for and delivery of intervention	• Interviews and workshops with adolescents, healthcare providers,

For peer review only

components; diagnostic accuracy; youth-friendly health services quality assessment.	community members, teachers, parents and key stakeholders<br>• Observations of the Y-Check visits and of selected referrals, including youth friendly services<br>• Self-reported screening tool
**Economic outcomes**	
Cost of setting up and running the intervention.	• Y-Check documentation and financial records<br>• Interviews with Y-Check staff and staff of the referral facilities.<br>• Programme monitoring data including records of attendance for referrals<br>• Screening tool (self-reported symptoms or conditions, measurements and clinical actions)
Cost per adolescent with a newly diagnosed condition (overall and by condition).	
Cost per adolescent with a newly diagnosed condition who received appropriate on-the-spot care or who completed an appropriate referral within 4 months (overall and by condition).	
Short-term (4 months) cost-effectiveness: cost per improvement in health or wellbeing (e.g. cost per case addressed or cured), cost per unit improvement in QALYs and per DALY averted.	
**Client outcomes**	
Knowledge about health services and health behaviours.	• Programme monitoring data including records of attendance for referrals<br>• Screening tool (self-reported symptoms or conditions, measurements and clinical actions)<br>• Self-completed evaluation questionnaire
Intentions to adopt healthy behaviours.	
Agency to make decisions about health and wellbeing.	
Perceived social support for behaviour change.	
Health-related risk and protective behaviours.	
Improvement in previously diagnosed health and wellbeing conditions.	
Engagement with health and other services within the past 4 months.	
Self-esteem.	
Self-perceived wellbeing.	
Quality of life.	
Clinical outcomes.	
Educational outcomes (e.g. school attendance).	• Self-completed evaluation questionnaire<br>• School register review
Client-defined outcomes (to be determined).	• Self-completed evaluation questionnaire<br>• Exit interviews

Statistical analysis

All primary analyses will be conducted separately by study city; Cape Coast, Chitungwiza and Mwanza. Where comparable, secondary analyses will be conducted with the data from all three cities combined.

In our study sites, a contemporaneous comparison group is not required since no routine screening is currently taking place, and as a result, assessments at baseline will serve as the counterfactual for internal

For peer review only

comparisons. Similarly, since there is no routine screening and treatment provided to adolescents of the target ages in the study population, a before-after comparison is appropriate since it is plausible to assume that reductions in the prevalence of the chronic conditions between the original Y-Check visit and the follow-up at four months will be due to the interventions provided through Y-Check.

We will follow STROBE guidelines for the reporting of cohort studies. Descriptive analyses will be used to compare the community-level and school-level characteristics of the study communities and schools. Quantitative programmatic data, including screening test results, services delivered, and referrals made and completed, will be reported by age, sex, and city. The primary outcome is a single proportion which will be presented with a 95% confidence interval for each of the 4 target groups: 10–14-year-old males, 10-14 year-old females, 15-19 year-old males, 15-19 year-old females.

Secondary outcomes which are measured at a single time point will be presented in a similar way to the primary outcome. For outcomes which are measured at two or more time points, a before-after analysis will be conducted comparing differences in measures between the time points. The unit of analysis will be the individual. For clinical outcomes which are measured at two or more time-points, the initial check-up visit (baseline) will give the prevalence of untreated conditions which will represent the counterfactual. The prevalence of conditions at the 4-month follow-up visit will be formally compared to this counterfactual to estimate the short-term effects of the intervention in improving these clinical outcomes. For analysis of outcomes measured at two timepoints we will use mixed effects logistic regression (binary outcomes) or linear regression (continuous outcomes) adjusting for individual-level clustering as a random effect and school/community as a fixed effect. Health service and client determinants of correct management of conditions at 4 months will be analyzed using multivariable regression.

Discussion

For peer review only

Over the last decade, adolescent wellbeing has become a global priority (WHO, 2023). School health is also a growing area of policy interest (WHO and UNESCO, 2021). WHO guidelines on school health services note that along with health promotion, health education, preventive interventions (such as immunizations and mass drug administration), clinical assessment and health services management, health screenings within school learners are one of the key pillars in the delivery of comprehensive school health services (WHO, 2021a). Screening programs such as Y-Check provide a unique opportunity to detect easily treatable, high-burden health conditions, refer those requiring medical attention, treatment and care, as well as to advise and encourage adolescents to engage in healthy behaviours.

In a 2015 review, school health services were found to exist in at least 102 countries though their content varied considerably across 16 areas including vaccinations, sexual and reproductive health education, vision screening, nutrition screening, and nutrition health education (Baltag *et al.* 2015). If all types of screening were combined, they were the second most commonly reported intervention in school health services, second only to immunization. A later systematic review found evidence of routine health check-ups of school age children having been reported in 86 countries worldwide (Baltag and Moran, 2018). Despite their widespread existence, little quality evidence exists on how to promote good health for adolescents in educational settings (Baltag *et al.* 2015), and even less for multi-component school health services (Levinson *et al.* 2019) especially in low- and middle-income countries (Montgomery *et al.* 2021).

Good practices in conducting adolescent health or wellbeing screenings are rarely reported. In 2023, WHO will release new guidance on well-child and well-adolescent visits, which will recommend expanding routine screening tests to also integrate other wellbeing dimensions through a broader evaluation of social risks, emotional state, and individual and family resources delivered with context-specific recommendations at key moments during the first two decades of life. The successful implementation of

For peer review only

such guidance requires robust measurement of the effectiveness of preventive interventions in adolescence (Banati *et al.*, 2023).

Evaluation of the Y-Check intervention will incorporate implementation science and effectiveness research. Such hybrid designs have important advantages over conducting separate studies. These include the potential for quicker translation of intervention research findings into programmes, the development and selection of more effective implementation strategies, and more useful information for decision makers (Curran *et al*. 2012).

The process evaluation findings will provide guidance for the next stage of the programme and for potential future sustainable and scalable implementation by local health authorities should it prove successful. Data on the short-term changes in clinical and behavioural outcomes will be used as inputs to model both short-term and long-term health and social impacts and as inputs to sample size and power calculations for a third phase of the Y-Check research programme, which plans to undertake a rigorous population level evaluation of the impact of routine check-ups on adolescent health and wellbeing.

Through WHO's advice to member states, findings from the Y-Check study have the potential to shape the delivery of adolescent health check-ups globally including identifying the optimal number, content and delivery for these services. Y-Check will advance the field by providing some of the first rigorous information on the effects of a health screening programme in three African cities, assessing implementation, effectiveness, cost and cost-effectiveness outcomes.

Acknowledgements

Programme Advisors include: Prof Simon Gregson, Dr. Sachin Shinde, Prof Fred Binka, Dr. Suzanne Petroni, Prof. Audrey Pettifor, Prof Shane Norris, Prof. James Hargreaves, Ms. Patience Manhibi, Mr Aveneni

For peer review only

Mangome. The DSMB Chair is Professor Fred Binka. DSMB members are Prof. David Mabey, Dr Andrew Abassa and Dr Nothando Ngwenya.

References

Baltag, V., Pachyna, A., & Hall, J. (2015). Global overview of school health services: data from 102 countries. Health Behavior and Policy Review, 2(4), 268-283.

Baltag V, Moran D. Are Routine Health Examinations Fulfilling their Promise for Prevention? Health Behavior and Policy Review 2018; 5(6): 22.

Banati, P., Banerjee, A., Daelmans, B., & Baltag, V. (2023). The need for new models to measure the impact of prevention. *Journal of Adolescent Health*, *72*(1), 9-11.

Bundy DAP, de Silva N, Horton S, Patton GC, Schultz L, Jamison DT. Investment in child and adolescent health and development: key messages from Disease Control Priorities, 3rd Edition. Lancet 2018; 391(10121): 687-99.

Chingono RM, Mackworth-Young CRS, Ross DA, Tshuma M, Chiweshe T, Nyamayaro C, Sekanevana C, Doyle A, Weiss HA, Mangombe A, Madzima B, McHugh G, Ferrand RA. Designing routine health check-ups for adolescents in Zimbabwe. *J Adolescent Health* 2021;**69**:940-947.

Curran GM, Bauer M, Mittman B, Pyne JM, Stetler C. Effectiveness-implementation hybrid designs: combining elements of clinical effectiveness and implementation research to enhance public health impact. Medical care 2012; 50(3): 217-26.

Damschroder LJ, Aron DC, Keith RE, Kirsh SR, Alexander JA, Lowery JC. Fostering implementation of health services research findings into practice: a consolidated framework for advancing implementation science. Implementation science : IS 2009; 4: 50.

Davis, M., & Fang, A. (2021). Emancipated Minor. In *StatPearls [Internet]*. StatPearls Publishing.

Ghana Health Service. Adolescent health service policy and strategy, 2016-2020. Accra, Ghana Health Service, 2016.

Glasgow RE, Vogt TM, Boles SM. Evaluating the public health impact of health promotion interventions: the RE-AIM framework. Am J Public Health 1999; 89(9): 1322-7.

Glasgow RE, Harden SM, Gaglio B, et al. RE-AIM Planning and Evaluation Framework: Adapting to New Science and Practice With a 20-Year Review. Frontiers in Public Health 2019; 7: 64.

Government of Zimbabwe. Zimbabwe School Health Policy. Harare, Zimbabwe, 2018.

Government of Tanzania. Health Sector Strategic Plan July 2021 – June 2026 (HSSP V): Leaving No One Behind. Ministry of Health, Community Development, Gender, Elderly and Children, Dar es Salaam, 2021.

Green, L., & Kreuter, M. (1999). The precede–proceed model. Health promotion planning: an educational approach. 3rd ed. Mountain View (CA): Mayfield Publishing Company, 32-43.

For peer review only

Hoffmann, T. C., Glasziou, P. P., Boutron, I., Milne, R., Perera, R., Moher, D., ... & Michie, S. (2014). Better reporting of interventions: template for intervention description and replication (TIDieR) checklist and guide. Bmj, 348.

Ilemela Municipal Council. Ilemela Municipal Council Strategic Plan (2016/2021).; 2017. doi:10.5089/9781513547442.002

ITU (2020) Young people more connected than the rest of the population

Kruk, M.E., et al., *Improving health and social systems for all children in LMICs: structural innovations to deliver high-quality services.* Lancet, 2022. **399** (10337): p. 1830-1844.

Kuper H, Monteath-van Dok A, Wing K, et al. The impact of disability on the lives of children; cross-sectional data including 8,900 children with disabilities and 898,834 children without disabilities across 30 countries. PLoS One 2014; 9(9): e107300.

Levinson, J., Kohl, K., Baltag, V., & Ross, D. A. (2019). Investigating the effectiveness of school health services delivered by a health provider: a systematic review of systematic reviews. PLoS One, 14(6), e0212603.

McLeroy, K. R., Bibeau, D., Steckler, A., & Glanz, K. (1988). An ecological perspective on health promotion programs. Health education quarterly, 15(4), 351-377.

Montgomery P, Knerr W, Ross DA, Patterson J. The effectiveness and acceptability of comprehensive and multicomponent school health services: A systematic review. *J Adolescent Health* 2022;70:192-207.

Moore GF, Audrey S, Barker M, et al. Process evaluation of complex interventions: Medical Research Council guidance. BMJ 2015; 350: h1258.

Nagata JM, Hathi S, Ferguson BJ, Hindin MJ, Yoshida S, Ross DA. Research priorities for adolescent health in low- and middle-income countries: A mixed-methods synthesis of two separate exercises. *J Global Health* 2018;**8**(1): doi: 10.7189/jogh.08.010501

National Bureau of Statistics. Mwanza Region - Basic Demograhic and Socio-Economic Profile (2012 Census) - National Bureau of Statistics (NBS).; 2016.

Patton, G. C., Sawyer, S. M., Santelli, J. S., Ross, D. A., Afifi, R., Allen, N. B., ... & Viner, R. M. (2016). Our future: a Lancet commission on adolescent health and wellbeing. The Lancet, 387(10036), 2423-2478.

Royal Australasian College of General Practitioners. Guidelines for Preventive Activities in General Practice, Volume Spec No. 8th ed. East Melbourne: Royal Australasian College of General Practitioners, 2012.

Sanci L. Clinical preventive services for adolescents: facing the challenge of proving "an ounce of prevention is worth a pound of cure". J Adolesc Health 2011; 49(5): 450-2.

Sedekia Y, Mshana G, Nsanya MK, Kohl K, Wambura M, Grosskurth H, Ross DA, Kapiga S. Routine health check-ups for adolescents in Mwanza City, Tanzania: Stakeholders' recommendations on its content, venue, and mode of delivery. Submitted to the BMC Public Health on 7th Nov 2022.

Skivington K, Matthews L, Simpson SA, Craig P, Baird J, Blazeby JM, Boyd KA, Craig N, French DP, McIntosh E, Petticrew M, Rycroft-Malone J, White M, Moore L. A new framework for developing and

For peer review only

evaluating complex interventions: update of Medical Research Council guidance. BMJ. 2021 Sep 30;374:n2061. doi: 10.1136/bmj.n2061. PMID: 34593508; PMCID: PMC8482308.

Tomlinson M, Ross DA, Bahl R, et al. What will it take for children and adolescents to thrive? The Global Strategy for Women's, Children's, and Adolescents' Health. The Lancet Child & adolescent health 2019; 3(4): 208-9.

UNESCO (2023) Net enrollment statistics available at htttp://uis.unesco.org. Downloaded March 18 2023

Weobong B, Glozah FN, Abdulai HBT, Koka E, Addae N, Alor S, Kohl K, Banati P, Adongo PB, Ross DA. Reaching adolescents with health services: systematic development of an adolescent health check-ups programme in Ghana (Y-Check, Ghana). (in preparation).

WHO (2012) Making health services adolescent friendly: developing national quality standards for adolescent friendly health services, 2012.

WHO (2014) Health for the World's Adolescents: A second chance in the second decade. Geneva: World Health Organization 2014.

WHO (2015) Global standards for quality health-care services for adolescents: a guide to implement a standards-driven approach to improve the quality of health care services for adolescents. See more at https://apps.who.int/iris/handle/10665/183935

WHO (2017) Global accelerated action for the health of adolescents (AA-HA!): guidance to support country implementation, 2017.

WHO (2019) WHO COVID-19 Case definition. See more at: https://www.who.int/publications/i/item/WHO-2019-nCoV-Surveillance_Case_Definition-2020.2

WHO (2020) Report of Y check Phase 1 to Botnar Foundation.

WHO (2020b) Ethical standards for research during public health emergencies: Distilling existing guidance to support COVID-19 R&D : https://www.who.int/blueprint/priority-diseases/key-action/liverecovery-save-of-ethical-standards-for-research-during-public-health-emergencies.pdf

WHO (2021a). WHO Guideline on School Health Services. 2021.

WHO (2021b). Assessing and supporting adolescents' capacity for autonomous decision-making in health care settings: a tool for health-care providers. Geneva: World Health Organization

WHO (2023). Accelerated Action for the Health of Adolescents (AA-HA! 2.0) Forthcoming. Geneva: World Health Organization

WHO and UNESCO (2021). Making every school a health-promoting school: global standards and indicators for health-promoting schools and systems Geneva; World Health Organization and UNESCO.

World Bank (2019). World Development Report 2019: The Changing Nature of Work. Washington, DC: World Bank, 2019.

World Bank. Gross primary school enrollment (Tanzania). Published 2021. https://data.worldbank.org/indicator/SE.PRM.ENRR?locations=TZ

For peer review only

World Bank. Gross secondary school enrollment (Tanzania). Published 2021b. https://data.worldbank.org/indicator/SE.SEC.ENRR?locations=TZ

Zimbabwean Ministry of Health and Child Care (MOHCC Z. Implementation Plan for HIV Pre-exposure Prophylaxis in Zimbabwe, 2018-2020, 2019.

Zimbabwe Ministry of Primary and Secondary Education (MoPSE). 2021 Primary and Secondary Education Statistics Report. Published May 2022. http://mopse.co.zw/sites/default/files/public/downloads/EMIS%202021%20Annual%20Education%20Statistics%20Report.pdf

Zimbabwe National Statistics Agency (ZIMSTAT). Education Statistics Report 2018-2020. Published November 2021. https://www.zimstat.co.zw/wp-content/uploads/publications/Social/Education/Education_Report_2020.pdf

For peer review only

Figure 1: Theory of Change for Y-Check, an adolescent health and wellbeing check-up

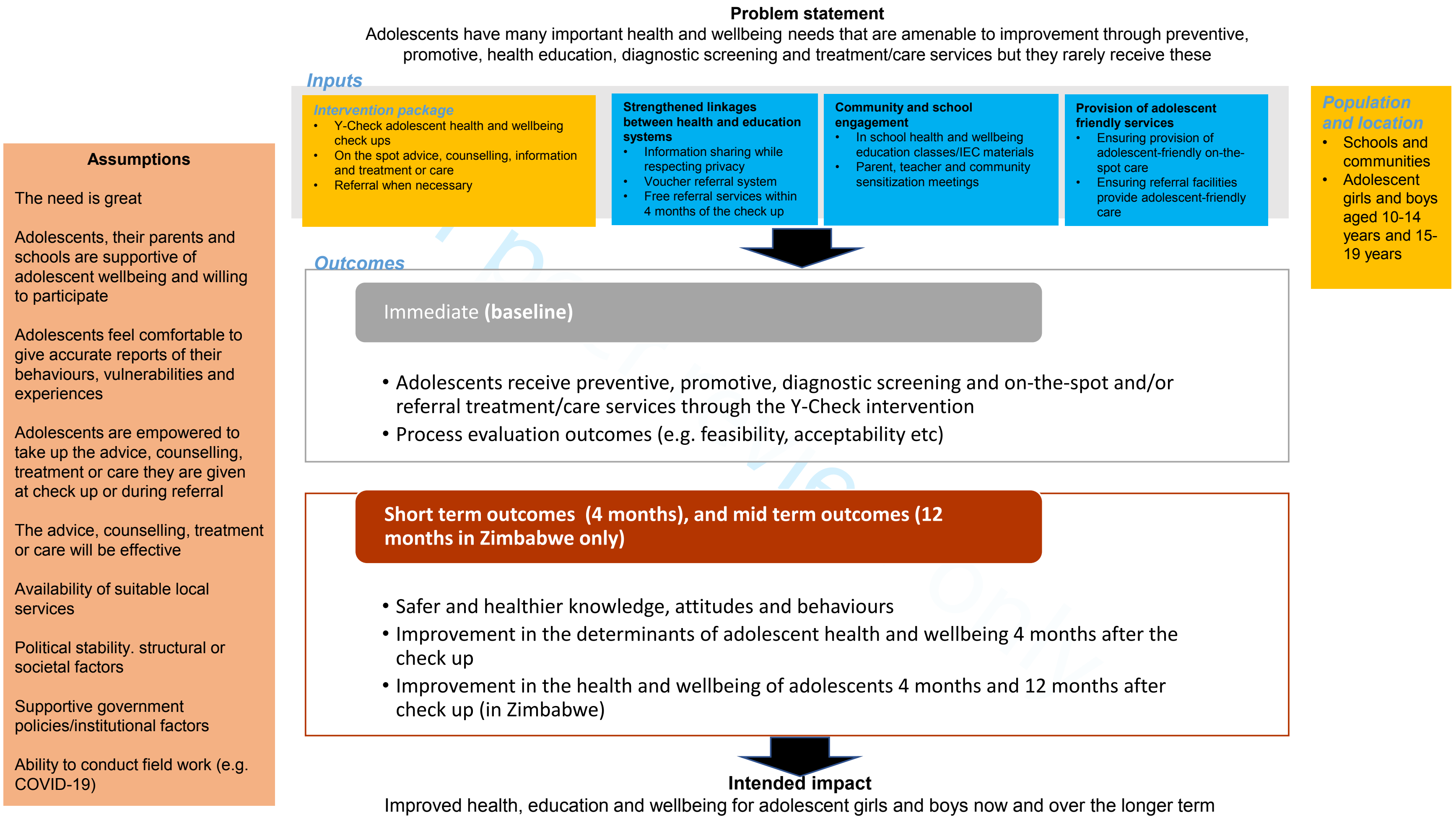

Figure 2: The Y-Check Intervention package[1]

Y-Check screening, on-the-spot care and referral services

Station 1
Registration and private pre-consultation screening questionnaire including tobacco and substance use, physical activity, diet, psychosocial and mental health, sexual activity (only in community settings) and other areas

Station 2
Physical examination including blood pressure, anthropometry, oral, vision and hearing exams and physical impairment

Station 3
Laboratory/ point of care tests including haemoglobin, HIVs and STIs (only in community settings), malaria, sickle cell and schistosomiasis (high prevalence cities only)

Station 4
Consultation review and intervention with clinician/nurse including on-the spot care for iron folic acid treatment, PrEP or STI treatment or contraception (sexual activity in community settings only) and further referral to services if indicated

Station 5
Health commodities Participants receive tooth cleaning kit, counselling, menstrual health kit (only girls), health promotion literature

For peer review only

[1] The intervention package may vary according to setting

For peer review only - http://bmjopen.bmj.com/site/about/guidelines.xhtml

Table 1: Template for Intervention Description and Replication (TIDieR) checklist describing the Y-Check intervention

Item	Item
**Brief name**	
1	Evaluating the effectiveness of adolescent health check-ups (Y-Check)
**Why?**	
2	Identifying adolescents with poor health, health-compromising behaviours or undiagnosed disability is important for their health and wellbeing, and also for communities and nations<br>Most adolescents only come into contact with health services when they are ill, and services are not always appropriate for their needs<br>Routine health and wellbeing check-up visits for adolescents that screen for multiple preventable and/or treatable conditions and risk behaviours could provide an entry point into services and be highly cost-effective
**What?**	
3	The intervention includes a comprehensive health check-up for priority conditions customized to national and local contexts.<br>Where indicated, Y-Check will provide on-the-spot care and cover all clinical costs associated with referrals to further care provided by the public health system or non-governmental organizations (NGOs).<br>During the check-up, adolescents will receive health promotion information and limited supplies of key health commodities.<br>Clinical costs of services are covered by the study if accessed within 4 months of the check-up.
4	Adolescent-friendly services will be provided, as defined by WHO (2018). Nationally-approved protocols will be applied. Adolescent privacy and confidentiality will be protected.
**Who provided?**	
5	Y-Check teams will be staffed with health professionals trained to provide quality adolescent-friendly health services in line with nationally-approved protocols. Y-Check teams will also be trained in the use of the digital application which will be used for data collection. Public and private not-for-profit care facilities providing referrals will meet national accreditation guidelines.
**How?**	
6	The Y-Check service will take place over a 60-90 minute period face-to-face. Any referrals will only be subsidized by the study if they take place within 4 months.
**Where?**	
7	The Y-Check service will be provided in schools and community venues, in outdoor tents where required.<br>Referrals will be to public or private not-for-profit providers as close as possible to the adolescent´s home. Providers will be vetted by the study team as being able to provide the necessary referral services to national and WHO-recommended standards.
**When and How Much?**	
8	Within the current phase of the study, each adolescent will receive Y-Check once. Within a routine programme the intention would be that the intervention will be delivered twice during adolescence, once when the adolescent is 10-14 years old, and a second time when they are 15-19 years old.
**Tailoring**	
9	The content of the intervention is tailored to local context. The exact set of conditions that will be assessed as part of Y-Check will be adapted based on burden of disease, and availability of local tests and referral services.
**Modifications**	

Item	Item
10	Any modifications will be reported in the article reporting the results of the study.
**How well?**	
11	Intervention fidelity (adherence, integrity, quality) will be evaluated through a process evaluation including youth-friendly health services quality.
12	Intervention fidelity will be reported in the article reporting the results of the study.

For peer review only

BMJ Open

An adolescent health and wellbeing check-up programme in three African cities (Y-Check): protocol for a multimethod, prospective, hybrid implementation-effectiveness study

Journal:	*BMJ Open*
Manuscript ID	bmjopen-2023-077533.R1
Article Type:	Protocol
Date Submitted by the Author:	08-Apr-2024
Complete List of Authors:	Banati, Prerna; World Health Organization,<br>Ross, David Anthony; Stellenbosch University<br>Weobong, Benedict; University of Ghana<br>Kapiga, Saidi ; National Institute for Medical Research Mwanza Research Centre; London School of Hygiene & Tropical Medicine<br>Weiss, Helen; London School of Hygiene & Tropical Medicine<br>Baltag , Valentina; World Health Organization<br>Nzvere, Farirai; London School of Hygiene & Tropical Medicine; Biomedical Research and Training Institute<br>Glozah, Franklin; University of Ghana<br>Nsanya, Mussa; National Institute for Medical Research Mwanza Research Centre<br>Greco, Giulia; London School of Hygiene & Tropical Medicine<br>Ferrand, Rashida; London School of Hygiene & Tropical Medicine, Department of Infectious Disease Epidemiology; Biomedical Research and Training Institute<br>Doyle, Aoife; London School of Hygiene & Tropical Medicine; Biomedical Research and Training Institute
**Primary Subject Heading**:	Public health
Secondary Subject Heading:	Evidence based practice
Keywords:	Adolescent, PUBLIC HEALTH, Mass Screening, Schools

SCHOLARONE™
Manuscripts

An adolescent health and wellbeing check-up programme in three African cities (Y-Check): protocol for a multimethod, prospective, hybrid implementation-effectiveness study

Banati P, PhD MPH[1*], Ross DA, MA, BMBCh, MSc, PhD[2] Weobong B, MSc, PhD[3], Kapiga S, MD, MPH, ScD[4,5], Weiss HA PhD MSc[6], Baltag V MD PhD[1], Nzvere F[5,7], Glozah F, MPhil, PhD[3], Nsanya MK, MD, MSc[4], Greco G, PhD MSc[5], Ferrand RA, PhD MBBS[5,7], and Doyle AM, PhD[6,7] for the Y-Check Research Programme Team[8]

[1] Adolescent and Young Adult Health (AYH) Unit, Maternal, Newborn, Child and Adolescent Health and Ageing (MCA) Department, World Health Organization (WHO), Ave Appia 20, 1211 Geneva 27, Switzerland
[2] Institute for Life Course Health Research, Stellenbosch University, Tygerberg, South Africa
[3] University of Ghana School of Public Health, College of Health Sciences (UGSPH), Ghana
[4] Mwanza Intervention Trials Unit, National Institute for Medical Research Mwanza Research Centre (MITU/NIMR), Isamilo, Mwanza, United Republic of Tanzania
[5] Department of Infectious Disease Epidemiology, London School of Hygiene and Tropical Medicine, Keppel St, London WC1E 7HT, UK
[6] MRC International Statistics and Epidemiology Group, London School of Hygiene & Tropical Medicine, Keppel St, London WC1E 7HT, UK
[7] Biomedical Research and Training Institute (BRTI), 10 Seagrave Road, Avondale, Harare, Zimbabwe
[8] The Y-Check Research Programme Team in alphabetical order includes Prof. Philip B. Adongo, MA, PhD, Department of Social and Behavioural Sciences, School of Public Health, University of Ghana; Mr. Kenneth. S. Adde, MPhil, Department of Population and Health, University of Cape Coast; Prof Evans Agbeno, FGCS, MPH, MBCHB, Department of Obstetrics & Gynaecology, University of Cape Coast; Prof. Patricia Akweongo, MA, PhD, Department of Health Policy Planning and Management, School of Public Health, University of Ghana; Ms Tsitsi Bandason MSc, The Health Research Unit Zimbabwe/ Biomedical Research and Training Institute; Dr. Sarah Bernays, PhD MA, Global Health and Development, London School of Hygiene and Tropical Medicine and School of Public Health, University of Sydney; Dr Rudo Chingono PhD, The Health Research Unit Zimbabwe/ Biomedical Research and Training Institute Dr Chido Dziva Chikwari PhD, MSc, BSc, The Health Research Unit Zimbabwe/ Biomedical Research and Training Institute and MRC International Statistics & Epidemiology Group, Department of Infectious Disease Epidemiology, London School of Hygiene & Tropical Medicine, London, UK; Ms Ethel Dauya MPH, The Health Research Unit Zimbabwe/ Biomedical Research and Training Institute; Dr. S. Derry, PhD, Department of Bio-Statistics, School of Public Health, University of Ghana; Dr. Eric Koka, MPhil, PhD, Department of Sociology and Anthropology, University of Cape Coast; Dr. Constance Mackworth-Young, PhD MSc, Global Health and Development, London School of Hygiene and Tropical Medicine, Ms. Salome Manyau MSc, The Health Research Unit Zimbabwe/ Biomedical Research and Training Institute; Mr. Bernard A. Owusu, MPhil, Department of Population and Health, University of Cape Coast; Dr. Yovitha Sedekia, MPHDC, PhD, Mwanza Intervention Trials Unit (MITU)/National Institute for Medical Research (NIMR); Dr. Hannah Taylor-Abdulai, MPH, MPhil, PhD, Department of Physician Assistant Studies, University of Cape Coast; ; Dr. Mandi Tembo, PhD, The Health Research Unit Zimbabwe/ Biomedical Research and Training Institute and Department of Global Health and Development, Faculty of Public Health and Policy, London School of Hygiene and Tropical Medicine, London, United Kingdom.

* Corresponding Author

Email addresses

Banati P (banatip@who.int); Ross DA (dross.rbridge@gmail.com); Weobong B (bweobong@ug.edu.gh); Kapiga S (Saidi.Kapiga@lshtm.ac.uk); Weiss HA (Helen.Weiss@lshtm.ac.uk); Baltag V (baltagv@who.int); Nzvere F (Farrie.Nzvere@lshtm.ac.uk); Glozah F (fglozah@ug.edu.gh); Nsanya MK (mussa.nsanya@mitu.or.tz); Greco G (Giulia.Greco@lshtm.ac.uk); Ferrand RA (rashida.ferrand@lshtm.ac.uk); Doyle AM (aoife.doyle@lshtm.ac.uk)

For peer review only

For peer review only - http://bmjopen.bmj.com/site/about/guidelines.xhtml

48 **Article Summary**

49 **Abstract**

50 Background: During adolescence, behaviours are initiated that will have substantial impacts on the individual's short- and long-term health and wellbeing. However, adolescents rarely have regular contact with health services, and available services are not always appropriate for their needs. We co-developed with adolescents a health and wellbeing check-up programme (Y-Check). This paper describes the methods to evaluate the feasibility, acceptability, short-term effects, and cost-effectiveness of Y-Check in three African cities.

56 Method: This is a multi-country prospective intervention study, with a mixed-method process evaluation. The intervention involves screening, on-the-spot care and referral of adolescents through health and wellbeing check-up visits. In each city, 2000 adolescents will be recruited in schools or community venues. Adolescents will be followed-up at 4 months. The study will assess the effects of Y-Check on knowledge and behaviours, as well as clinical outcomes and costs. Process and economic evaluations will investigate acceptability, feasibility, uptake, fidelity and cost effectiveness.

62 Ethics and Dissemination: Approval has been received from the WHO (WHO/ERC Protocol ID Number ERC.0003778); Ghana Health Service (Protocol ID number GHS-ERC: 027/07/22), the United Republic of Tanzania National Institute for Medical Research (Clearance No. NIMR/HQ/R.8a/Vol.IX/4199), the Medical Research Council of Zimbabwe (Approval number MRCZ/A/2766), and the LSHTM (Approval numbers 26395 and 28312). The trial registration number is NCT06090006. Consent and disclosure are addressed in the paper. Results will be published in 3 country-specific peer reviewed journal publications, and one multi-country publication; and disseminated through videos, briefs, and webinars. Data will be placed into an open access repository. Data will be deidentified and anonymized.

For peer review only - http://bmjopen.bmj.com/site/about/guidelines.xhtml

Discussion: This study is innovative in evaluating a comprehensive adolescent health and wellbeing check-up intervention which addresses both health conditions that impact on wellbeing during adolescence, and risk factors for future health or wellbeing. Findings will support larger scale intervention implementation and longer-term evaluation.

Keywords: Adolescent, health, wellbeing, check-ups, screening, implementation research, effectiveness, cost-effectiveness

For peer review only

For peer review only - http://bmjopen.bmj.com/site/about/guidelines.xhtml

Additional information

Strengths and limitations of the methodology:

- **Strength**: This study will utilize existing health care infrastructure in low- and middle-income country settings, assessing real world implementation situations and therefore it will be relatively straightforward to directly apply the findings to programs.
- **Strength**: This is a relatively large study of 6000 adolescents in 3 countries. The study takes the views of young people centrally into the design of the intervention.
- **Limitation**: Although the primary outcome is an implementation science / programmatic outcome, the effectiveness data is based on pre-post comparison.
- **Limitation**: This study will have limited ability to assess sustainability of effects over the longer term as the follow up period is 4 months
- **Limitation**: This study is operating in three African cities which may limit generalizability to rural areas.

Manuscript Wordcount: 5023

For peer review only

Background

To unlock human potential and accelerate progress towards achieving the Sustainable Development Goals (SDGs), it is essential to improve the health and wellbeing of adolescents (10-19 years) (1). Health is an essential component of human capital (2), yet adolescent investments have focused primarily on either health or education services with little attention to synergies between these (3). Research investments in the first 1000 days of life have dramatically outweighed investments in the subsequent 7000 days, leaving an evidence gap on how to develop and sustain human potential through adolescence and early adulthood (4).

Among adolescents in low- and middle-income countries (LMICs), HIV/AIDS, road injury, diarrheal diseases, self-harm, iron-deficiency anemia and skin diseases are among the top causes of morbidity and mortality (5, 6, 7). Identifying adolescents with poor health, health-compromising behaviours or undiagnosed disability is important given (a) the growing number of adolescents and their low frequency of regular contacts with health services (8) (b) the high proportion of the total global burden of disease that occurs in adolescence and (c) the fact that many key health conditions (e.g. mental health disorders) and behaviours (e.g. tobacco and alcohol use, unhealthy diet, low physical activity, risky sexual behaviours) that predispose to preventable serious conditions in later life start in adolescence (d) the negative impact of poor health on educational attainment and employability and other transitions to healthy adulthood, and (e) gender-related vulnerabilities, including violence, abuse, unintentional injury, sexual and reproductive health (SRH) and gendered mental health outcomes which may emerge or be exacerbated during this period of life, setting negative trajectories to lifetime and intergenerational health and wellbeing (4).

Systematic reviews have identified individual interventions that are effective at improving various aspects of adolescent health and/or wellbeing (4) However, most adolescents only come into contact with health

For peer review only - http://bmjopen.bmj.com/site/about/guidelines.xhtml

services when they are ill, and services are not always appropriate for their needs (9). This represents a missed opportunity for early detection of health problems, for health promotion, and for the development of health-seeking behaviours. Early and sustained engagement with health and social services could reap a triple dividend for human development by improving the health and wellbeing of adolescents, their health and wellbeing in adulthood and the health and wellbeing of their future offspring (2, 4, 10)

Routine health and wellbeing check-up visits for adolescents that screen for multiple conditions and risk behaviours could provide an entry point into services and be highly cost-effective (11, 12). Obtaining evidence on the optimum content, delivery, effectiveness and cost of check-ups is a high priority for adolescent health research so that governments can be informed by the evidence on how to initiate or strengthen existing health and wellbeing check-ups during adolescence (13). Many high-income countries have national recommendations related to adolescent health check-ups, which have been largely based on expert opinion (14,15). In LMICs, if provided at all, preventive and promotive health services for adolescents are largely provided in schools and are usually limited to deworming and vaccination campaigns. They do not usually address other key conditions and risk factors such as nutrition, mental health, SRH or disability (16, 17). If a system-wide approach to check-ups exists in adolescence, in LMICs it is often limited to a screening activity without other components such as brief intervention or anticipatory guidance (17).

This paper describes the protocol for the Y-Check: Evaluating the effects of adolescent health check-ups study, a prospective hybrid implementation-effectiveness study evaluating the feasibility, acceptability, short-term effects, costs and cost-effectiveness of the Y-Check intervention in three African cities. This study has received approval from the World Health Organization (WHO/ERC Protocol ID Number ERC.0003778); Ghana Health Service (Protocol ID number GHS-ERC: 027/07/22), the United Republic of Tanzania National Institute for Medical Research (Clearance No. NIMR/HQ/R.8a/Vol.IX/4199), the

For peer review only - http://bmjopen.bmj.com/site/about/guidelines.xhtml

Medical Research Council of Zimbabwe (Approval number MRCZ/A/2766), and the London School of Hygiene and Tropical Medicine (Approval numbers 26395 and 28312) .

The Y-Check intervention

Y-Check is a novel intervention delivering a health and wellbeing check-up and where indicated will provide on-the-spot care and/or referral for common conditions on two occasions in adolescence (in young adolescents (10-14 year-olds) – soon after the onset of puberty - and in older adolescents (15-19 year-olds) – when many adolescents become, or are soon to become, sexually active). It will also provide health promotion information and materials to support positive behaviours and healthy lifestyles during adolescence and beyond. The intention is that in the context of a future routinely-delivered programme, every adolescent will have two guaranteed contacts with the health care system. Adolescents will only be screened for conditions that have an accurate, low-cost, acceptable screening test and a locally accessible, effective intervention. The conditions selected for screening will be chosen to reflect the local epidemiological contexts (e.g. screening for malaria will only take place in malaria endemic areas). Respecting specific requests from the Ministries of Education in all three cities, the study will only include sexual and reproductive health (SRH) screening and services at the community sites (which only include older adolescents).

Figures 1 and 2 present the Theory of Change and description of the intervention. Table 1 applies the TIDieR checklist (18) to describe details of the intervention.

Locally accessible services will be identified and assessed in terms of their ability to provide the services recommended by local and WHO guidelines, willingness to accept referred adolescents, and the fees charged to the project will be negotiated by the research team for services provided to referred

For peer review only - http://bmjopen.bmj.com/site/about/guidelines.xhtml

adolescents (where adequate services are not covered by national health insurance schemes, free NGO services or free public health care).

Insert Figures 1 and 2

For peer review only - http://bmjopen.bmj.com/site/about/guidelines.xhtml
For peer review only

Table 1: Template for Intervention Description and Replication (TIDieR) checklist describing the Y-Check intervention

Item	Item
**Brief name**	
1	Evaluating the effectiveness of adolescent health check-ups (Y-Check)
**Why?**	
2	Identifying adolescents with poor health, health-compromising behaviours or undiagnosed disability is important for their health and wellbeing, and also for communities and nations<br>Most adolescents only come into contact with health services when they are ill, and services are not always appropriate for their needs<br>Routine health and wellbeing check-up visits for adolescents that screen for multiple preventable and/or treatable conditions and risk behaviours could provide an entry point into services and be highly cost-effective
**What?**	
3	The intervention includes a comprehensive health check-up for priority conditions customized to national and local contexts.<br>Where indicated, Y-Check will provide on-the-spot care and cover all clinical costs associated with referrals to further care provided by the public health system or non-governmental organizations (NGOs).<br>During the check-up, adolescents will receive health promotion information and limited supplies of key health commodities.<br>Clinical costs of services are covered by the study if accessed within 4 months of the check-up.
4	Adolescent-friendly services will be provided, as defined by WHO (2018). Nationally-approved protocols will be applied. Adolescent privacy and confidentiality will be protected.
**Who provided?**	
5	Y-Check teams will be staffed with health professionals trained to provide quality adolescent-friendly health services in line with nationally-approved protocols. Y-Check teams will also be trained in the use of the digital application which will be used for data collection. Public and private not-for-profit care facilities providing referrals will meet national accreditation guidelines.
**How?**	
6	The Y-Check service will take place over a 60-90 minute period face-to-face. Any referrals will only be subsidized by the study if they take place within 4 months.
**Where?**	
7	The Y-Check service will be provided in schools and community venues, in outdoor tents where required.<br>Referrals will be to public or private not-for-profit providers as close as possible to the adolescent´s home. Providers will be vetted by the study team as being able to provide the necessary referral services to national and WHO-recommended standards.
**When and How Much?**	
8	Within the current phase of the study, each adolescent will receive Y-Check once. Within a routine programme the intention would be that the intervention will be delivered twice during adolescence, once when the adolescent is 10-14 years old, and a second time when they are 15-19 years old.
**Tailoring**	
9	The content of the intervention is tailored to local context. The exact set of conditions that will be assessed as part of Y-Check will be adapted based on burden of disease, and availability of local tests and referral services.
**Modifications**	

Item	Item
10	Any modifications will be reported in the article reporting the results of the study.
**How well?**	
11	Intervention fidelity (adherence, integrity, quality) will be evaluated through a process evaluation including youth-friendly health services quality.
12	Intervention fidelity will be reported in the article reporting the results of the study.

Methods/Design

Aims

The aim of the study is to develop and implement in three African cities a potentially sustainable adolescent health check-up programme, and evaluate the acceptability, feasibility, short-term effects, and cost-effectiveness of the programme to improve health and wellbeing. The study was launched in September 2021 and will run until June 2025.

Objectives

(1) To develop and pilot test a check-up programme for adolescents that screens for important preventable and treatable health conditions using accurate and acceptable screening tests and provides locally accessible effective interventions.

(2) Through a prospective intervention study in selected schools and communities to:

- Estimate short-term impacts on adolescent health and wellbeing outcomes: clinical outcomes, health-related knowledge and behaviours, intentions, agency, and perceived social support for behaviour change; engagement with health services.
- Understand, through process evaluation, the feasibility and fidelity of implementation, the acceptability and uptake, and the influence of context.

For peer review only - http://bmjopen.bmj.com/site/about/guidelines.xhtml

- Estimate the cost-effectiveness of the programme in reducing overall disease burden and improving adolescent wellbeing

(3) Obtain information on key parameters needed for the planning of an evaluation study: prevalence of health conditions and behaviours, acceptability of referral, feasibility of following-up programme participants and delivering quality follow-up care, initial estimates of the impact of the programme on longer-term health, educational and wellbeing outcomes based on the short-term implementation and effectiveness outcomes observed in this phase of the research programme, and factors related to the optimal implementation of the Y-Check intervention.

(4) To refine the programme and its theory of change, and finalise optimal methods for the measurement of the impact of the programme in future studies.

Patient and public involvement

The intervention was designed following formative research conducted in three African countries between 2019 and 2020 (19, 20, 21). This formative research revealed that the proposed adolescent health and wellbeing check-ups are likely to be feasible to implement and acceptable to stakeholders in Ghana, Tanzania and Zimbabwe, and are likely to meet the perceived needs of key stakeholders including adolescents, their parents, and key policy makers in the health and education sectors (22). Further, we showed that the programme is likely to produce a substantial yield of important, previously untreated, treatable conditions. Human-centered design techniques were used alongside desk review to define elements of objective and subjective importance to the health and wellbeing of adolescents, identify facilitators and barriers to adolescent health seeking, preferences for delivery of routine health check-ups, and potential effects of interventions to select the content and method of delivery of the Y-Check intervention. Interviews and participatory workshops with adolescents, parents of adolescents and key stakeholders from the ministries of health and education, non-governmental organizations, healthcare

For peer review only

For peer review only - http://bmjopen.bmj.com/site/about/guidelines.xhtml

workers and teachers found that there was overall support for the introduction of routine health check-ups (19, 20, 21). To navigate potential barriers, stakeholders suggested clear messaging, awareness building, and sensitization campaigns to overcome disinterest in preventative healthcare and, in some contexts, mitigate cultural or religious messaging against healthcare engagement (19).

Theory of Change

We hypothesise that a routine health and wellbeing check-up visit for adolescents that screens for multiple conditions and risk behaviours will have an immediate and long-term positive impact on health and wellbeing outcomes (Figure 1).

Health seeking and promotion behaviours among adolescents operate in complex environments and across ecological levels (10), with determinants at individual, interpersonal institutional/organizational, community and public policy levels. Drawing from the health promotion literature (23, 24), the Theory of Change for Y-Check (Figure 1) draws on thinking that recognizes pre-disposing, enabling and reinforcing factors as capacities to be strengthened in order to achieve adolescent wellbeing at the individual level; that responsive parenting can support adolescents to meet their own health and wellbeing goals; that systems-based approaches (including stronger linkages between health and education systems) can improve outcomes for adolescents, especially reaching the most vulnerable and those in need; and that an enabling environment (especially in schools and communities) can support adolescents to take action towards improving their health.

Study setting

Our study will be undertaken in three African cities: Cape Coast in Ghana, Mwanza in Tanzania and Chitungwiza in Zimbabwe. These cities are described in Table 1.

For peer review only - http://bmjopen.bmj.com/site/about/guidelines.xhtml

Table 1: The study cities, schools and communities

Cape Coast, Ghana	Mwanza, Tanzania	Chitungwiza, Zimbabwe
Cape Coast Metropolis is located on the coast of Ghana, 150kms west of the capital city, Accra. It has a population of 169,894 with three-quarters of the households residing in urban areas.<br><br>Literacy in 11-24 year-olds is about 97%. In 2016, 11,233 (68.8%) of 12-14 year-olds were enrolled in junior high schools while 8,407 (91.6%) of 15-17 year-olds were enrolled in senior high schools. For Ghana as a whole, primary and secondary net enrollment rates in 2019 were 86% and 57%, respectively (25)<br><br>There are 36 health facilities (26 public and 10 private) in the metropolitan area, including a regional hospital that serves as a secondary referral facility.<br><br>The study will be conducted in 8 schools and local community venues in four communities that include two relatively affluent communities with trading being the main source of livelihood and two relatively poorer communities where fishing and farming dominate, respectively.	Mwanza is located on the southern shores of Lake Victoria in North-Western Tanzania and is the second largest city in Tanzania with a population of over 900,000 and an annual growth rate of 3% (26). Economic activities in Mwanza include fishing and fish processing, subsistence agriculture and support services to nearby gold and diamond mines.<br><br>Adolescents make up 24.2% of the population of the city (Tanzania National Bureau of Statistics, 2016). As of 2020/21, the primary and secondary school net enrollment rates were 82% and 39%, respectively (26)<br><br>Available public health services include 26 dispensaries, 5 health centres, 2 district hospitals, 1 regional hospital and 1 tertiary/teaching hospital (26, 27).<br><br>The study will be conducted in 4–6 purposively-selected communities and in up to 8 primary schools and 8 secondary schools within the catchment area of health facilities serving the selected communities in the two districts within Mwanza city.	Chitungwiza is the third largest city in Zimbabwe, located approximately 25km south of the capital city, Harare. It has a population of about 456,000 (28). The houses are mostly high-density, single-story, detached units with small yards that are generally used for growing vegetables. Most of the people work in Harare, as there is little industry in Chitungwiza itself.<br><br>Zimbabwe has a school-going population (8-18 years) of approximately 4.3 million (29). Net primary enrollment rate across Zimbabwe is 94%; net secondary enrollment rate is 54% (28)<br><br>In Chitungwiza, there is one tertiary hospital, 4 public primary healthcare facilities, 20 private medical facilities, 30 government primary schools, and 13 government secondary schools (all mixed sex).<br><br>The study will be conducted in four distinct communities which are representative of the urban, peri-urban and rural populations of Chitungwiza. Eligible schools must have a student population of at least 200 learners in Grade 6 or at least 75 learners in Form 5; and be located in or close to one of the selected study communities.

Study design

In this prospective hybrid implementation-effectiveness study, 2000 adolescents per city who receive the Y-Check intervention will be followed up at 4-months, and at 12-months (Zimbabwe only).

Stakeholder engagement

In each city, the research study is undertaken in partnership with both the national and municipal Ministries of Health and Education. Each country has a policy framework that provides encouragement for the introduction of health and nutrition education and promotion among adolescents, including screening for communicable and non-communicable diseases, immunization, growth monitoring and assessments and nutritional services (30-32).

For peer review only

For peer review only - http://bmjopen.bmj.com/site/about/guidelines.xhtml

This study will build on stakeholder engagement, the process for which was established in each research setting during the formative phase. In each city, a Community Advisory Committee (CAC) comprising key community leaders and stakeholders will be reinforced or set up to facilitate input from, and feedback to, participating communities and a Youth Advisory Group (YAG) will provide a forum for adolescents to input into the programme. The YAG will meet with research staff at least 4 times per year, be active participants in programme design and dissemination workshops, and help to ensure that the programme meets the needs of adolescents. Community engagement will be an ongoing process through regular contacts with the CAC, the YAG and other stakeholders, such as teachers, health workers, Community Based Organizations (CBOs), Non-Governmental Organizations (NGOs), and religious leaders. In addition, a key aspect for building confidence within communities is the knowledge that the study has the support of the government.

Intervention development and pilot testing

Prior to implementation, preparatory activities will include community engagement, participatory co-design, negotiating referral arrangements and pre-testing of screening tools, procedures and referral protocols. Pilot studies in each setting will provide initial estimates of the frequency of health and behavioural outcomes, and help to refine the intervention model.

Pilot testing will involve the implementation of the screening tools and procedures with approximately 200 adolescents in each of the three cities with revisions and repeat pilot testing where required. Adolescents who participate in the pilot study will be excluded from the main study if the procedures change following the pilot. There will be an opportunity for young people and stakeholders to suggest additional client-centered outcomes that may reflect some of their priority concerns or intentions that should be captured.

For peer review only - http://bmjopen.bmj.com/site/about/guidelines.xhtml

Intervention implementation

The intervention will be delivered over a period of 2-6 months in each of the settings. The follow-up visits will take place at the same school or community setting as the initial check-up. In addition to covering all clinical costs, the equivalent of USD 5 will be given to each participant who attends the follow-up to cover any transport costs that they might have incurred. Additionally, health and hygiene related items will also be provided for adolescents to take home, including tooth cleaning kit (toothbrush and toothpaste), fruit, bottle of water, two pairs of underpants, pack of reusable sanitary pads (girls only)

Composition and training of Y-Check team

The Y-Check team will be trained to deliver adolescent-responsive and age-appropriate services according to national and WHO guidelines, recognizing also the needs for privacy and confidentiality (33). This includes providing services that are attractive to adolescents, meet their needs comfortably and responsively, and that are attentive to their privacy. These principles and approaches will be embedded into each part of the Y-Check intervention. Visual and auditory privacy will be prioritized, through the use of separate tents, rooms or screens. Health workers will employ standard gowning and draping for clinical procedures.

For infection prevention and control (IPC), all study procedures including interviews, physical examinations and blood tests will take place in well-aerated tents or outdoors, and will follow relevant nationally-approved protocols for all staff and participants.

The Y-Check team will be trained in good clinical practice, data protection and confidentiality, and clinical staff will be trained in counselling for participants testing positive for any of the conditions being screened for within Y-Check as well as in general counselling skills.

For peer review only

For peer review only - http://bmjopen.bmj.com/site/about/guidelines.xhtml

Inclusion and exclusion criteria

To be included in the study, adolescents aged 10-19 years must fall into one of the first three categories below and fulfil category 4.

1) Be attending selected classes of Year 5 of primary school in Mwanza (median age 11 years); Grade 5/6 of primary school in Chitungwiza (median age 11 years); or Year 1 of Junior Secondary School in Cape Coast (median age 12 years) OR

2) Be attending selected classes in Year 3 of Secondary School in Mwanza (median age 17 years), Form 3/4 in Chitungwiza (median age 17 years), or Year 2 of Senior Secondary School in Cape Coast (median age 16 years) OR

3) Be resident in a selected community during the time of the Y-Check intervention, and be aged 16-19 years

AND

4) Have a completed and signed Informed Consent form, or a signed Informed Assent Form and signed Parental/Guardian Informed Consent Form if the adolescent is seen in the community and is below the national age of consent or is seen in a school, irrespective of their age.

Consent and Assent procedures

Before the visit of the implementation team, information on the Y-Check programme will be distributed to parents/guardians through the schools and to community members through an active communication campaign in collaboration with the CAC and the YAG. School and community meetings will allow parents and community members to ask questions about the programme and give their feedback.

For peer review only - http://bmjopen.bmj.com/site/about/guidelines.xhtml

In schools, adolescents will have a short introductory meeting with a member of the Y-Check team typically in a class or group setting. Parents meetings will then be held in each of the schools, to which all the parents and guardians of eligible learners will be invited. During these sessions, information will be provided about the study, its objectives and procedures, possible risks and procedures that will be used to maintain confidentiality. These meetings will provide an opportunity for the adolescents, parents and guardians of eligible adolescents to learn more about the Y-Check intervention and the research linked to it and to have their questions answered.

No participants will be screened, receive care or be counselled or interviewed without their informed consent (community participants who are above the national age of consent), or, for minors, their assent and parental consent, unless they are determined to be emancipated minors (34). Following advice from Ministries of Education in all three countries, all adolescents seen in schools will be considered to be minors and require parental consent, irrespective of their age.

Minor adolescents' assent will be ascertained and documented in an assent form. Parents or guardians who would like their adolescent to receive the check-up will be asked to provide their written consent. On the day of the check-up visit, a verbal confirmation of their previous written assent will be requested from the adolescent. In Ghana and Tanzania, where the minimum age for providing consent to medical and health-related research is 18 years, clients of all ages under 18 will provide completed parental consent forms and provide written assent before proceeding through the check-up visit regardless of whether the check-up is in schools or communities. In Zimbabwe, a waiver of parental consent has been given by the Medical Research Council of Zimbabwe (MRC-Zimbabwe) so that participants aged 16 and 17 years who attend the check-ups in the community venues will be allowed to provide written consent for themselves.

For peer review only

For peer review only - http://bmjopen.bmj.com/site/about/guidelines.xhtml

The intervention will be conducted in private and not in the presence of the parent or guardian. Contact details of the study team will be shared with participants in case they have questions at a later stage. All participants will be reminded that participation is entirely voluntary and will be told that they can opt out of the research or services at any time.

Data collection

During the Y-Check intervention and follow up

Data collection during baseline and follow-up visits will include self-completed evaluation questionnaires, self-reported screening tool responses and screening visit consultations, measurements and specimen collection and an exit interview. Data on the implementation process and on adolescent outcomes will be collected in digital and paper-based formats. A user-friendly digital data collection app for the check-ups will be developed and housed on a tablet computer for direct use by the adolescent. Initial sections will include audio-assisted, user-friendly self-completion questions for adolescents to fill out. This will utilize engaging content and processes, tailored to adolescents´ interests. The option of a face-to-face interview will also be available if the adolescent is unable to use the tablet or has low literacy level. Health services registers and school registers will also be reviewed to determine the number of adolescents of the relevant age ranges, and school attendance by the classes involved in Y-Check. To help build the referral process, existing adolescent services will be mapped in the study communities.

Process evaluation

The process evaluation is guided by the UK MRC's Process Evaluation framework to understand intervention implementation (including feasibility and fidelity), mechanisms of impact (including acceptability and uptake), and the influence of context (35). Key implementation outcomes of interest are acceptability, adoption, appropriateness, feasibility, and fidelity. Data on contextual factors and barriers

For peer review only - http://bmjopen.bmj.com/site/about/guidelines.xhtml

and facilitators to programme implementation will be gathered using routinely-collected programme monitoring data. Qualitative data will be collected through 1) observations of the Y-Check intervention and referrals, as well as team meetings; 2) in-depth interviews with eligible adolescents who received, adolescents who were referred, and adolescents who did not receive Y-Check, as well as with school authorities and the Y-Check service providers; and 3) participatory workshops with teachers, adolescents, and parents. Quantitative programme monitoring data will be collected routinely within the Y-Check visit, including through a participant exit interview. Process evaluation data will be analysed iteratively and thematically, through regular analytical discussions and analytical memos to draw out the main themes emerging from the data. Across the pilot and intervention studies, data collection for the process evaluation will include real-time feedback to the implementation team.

Economic evaluation

A costing study will be conducted to estimate the total costs of developing, setting up, and running the Y-Check package, in school and community settings. A combination of top-down and ingredients-based costing approaches will be used to generate cost estimates for the whole package, and for each component/activity. All costs will be estimated from the perspectives of the adolescents, the schools/community and implementing partners/service providers. Financial and economic costs will be calculated for all inputs. These inputs will be identified and measured using process data, staff interviews and observations, document review, and accounting records.

Costs will be inputted and analysed in an Excel-based costing tool. The cost analysis will describe the distribution of costs across different forms of inputs, and will estimate the unit cost per adolescent reached, screened, and treated on the spot or referred; cost per unit of measure for selected process and effect outcomes such as cost per condition detected, cost per condition appropriately treated on-the-spot or with a completed referral within 4 months, cost for a unit improvement in reported quality of life and Disability Adjusted Life Years (DALYs) averted.

For peer review only

For peer review only - http://bmjopen.bmj.com/site/about/guidelines.xhtml

The cost and cost-effectiveness estimates will be compared to other programmes in the region (eg. human papillomavirus vaccination, deworming) and will inform programme replication, scalability, and financial sustainability.

Data protections

Data protection will be strictly observed. After study completion, data will be stored in the LSHTM-curated digital repository 'Data Compass' following General Data Protection Regulation (GDPR) guidelines. Data and code registered in LSHTM Data Compass will be made open access following deposit. A Data Safety and Monitoring Board (DSMB) has been constituted to assist in managing adverse events, though we expect these to be very rare since all treatment and care are standard with no novel treatments.

Study outcomes

Outcomes will be ascertained during the check-up screening visit and through collection of referral vouchers from the referral health facilities, and, for outcomes related to health and wellbeing impacts, through data from the 4-month and, in Zimbabwe only, 12-month follow-up visits. Outcomes related to completed referrals will be triangulated against participants' self-reports at the 4-month and. In Zimbabwe only, 12-month follow-up visits. Review of school and health service registers will be used to see whether attendance has increased during the period when Y-Check is being implemented.

The primary outcome will be the proportion of those screening positive for at least one condition who receive appropriate on-the-spot care or complete appropriate referral for all identified conditions within 4 months. This will be measured using data collected at the initial check-up visit and through recovery of referral vouchers given to participants to allow them to access referral services for free during the 4-months after the Y-Check screening. Completed referral is defined as attending at least the first referral appointment.

For peer review only

For peer review only - http://bmjopen.bmj.com/site/about/guidelines.xhtml

Secondary implementation outcomes will include the proportion of those screening positive for each condition who receive appropriate on-the-spot care or complete appropriate referral for that condition within 4 months, the yield of previously untreated conditions, clinical outcomes at 4 months among those who had originally screened positive for each condition, and intervention acceptability, adoption, appropriateness, feasibility, fidelity and cost. Secondary effectiveness outcomes will include knowledge about health services and health behaviours, self-reported agency and self-efficacy to make decisions about their health, self-reported health-related risk and protective behaviours, reported engagement with health services, wellbeing, self-esteem and quality of life, clinical outcomes, and educational outcomes, which will be collected within the Y-Check and follow-up visits. The short-term cost-effectiveness of the intervention will be estimated (calculated by a comparison of the costs of the intervention against the primary and secondary outcomes and including short-term changes in self-reported quality of life). All outcomes for the study are described in Table 2.

Sample size

In each city, the intervention will be implemented for 10-14 year-olds in up to 6 government primary schools (N=500 for young adolescent girls, and N=500 for young adolescent boys), and for 15-19 year-olds in up to 8 secondary schools and up to 3 community venues (N=500 for older adolescent girls, and N=500 for older adolescent boys), giving a total sample size of 2,000 adolescents (10-19y).

The sample size provides specified precision around the primary outcome. For example, for the primary outcome, within each age group and gender, if 150 (30%) of 500 participants screen positive for at least one condition, and 75% of those who screen positive are correctly managed (n=112), the 95% CI for correct management will be +/- 7%. The primary outcome used data from the initial check-up visit and referrals and did not require the 4-month follow-up data.

For peer review only

For peer review only - http://bmjopen.bmj.com/site/about/guidelines.xhtml

Table 2: Study outcomes and means of verification

Outcome	Sources of data
**Primary outcome**	
Proportion of those screening positive for at least one condition who receive appropriate on-the-spot care or complete appropriate referral for all identified conditions within 4 months (i.e. they attend a provider for referral care who has been accredited by the study team and has been shown to be capable of providing appropriate referral care).	• Programme monitoring data including records of attendance for referrals<br>• Screening tool (self-reported symptoms or conditions, measurements and clinical actions)
**Secondary outcomes**	
**Implementation outcomes**	
Proportion of those screening positive for each condition who receive appropriate on-the-spot care or complete appropriate referral for that condition within 4 months.	• Programme monitoring data including records of attendance for referrals<br>• Screening tool (self-reported symptoms or conditions, measurements, and clinical actions)
The yield of previously untreated conditions.	• Programme monitoring data including records of attendance for referrals<br>• Screening tool (self-reported symptoms or conditions, measurements, and clinical actions)
Intervention acceptability (satisfaction): acceptability to adolescents and to other stakeholders (eg. schools, parents, health workers).	• Programme monitoring data including records of attendance for referrals<br>• Screening tool (self-reported symptoms or conditions, measurements, and clinical actions)<br>• Self-completed evaluation questionnaire<br>• Exit interviews<br>• Observations of the Y-Check visits and of selected referrals<br>• Interviews and workshops with adolescents, healthcare providers, community members, teachers, parents and key stakeholders
Intervention adoption (uptake, utilization): Y-Check uptake, referrals completed.	
Intervention appropriateness (perceived fit, perceived relevance, perceived usefulness): perceived value of the intervention to adolescents and to other stakeholders.	
Intervention feasibility (actual fit, practicability): Y-Check visits completed, referrals completed, stakeholder support (including community).	
Intervention fidelity (adherence, integrity, quality): completeness of training for and delivery of intervention components; diagnostic accuracy; youth-friendly health services quality assessment.	• Interviews and workshops with adolescents, healthcare providers, community members, teachers, parents and key stakeholders<br>• Observations of the Y-Check visits and of selected referrals, including youth friendly services<br>• Self-reported screening tool
**Economic outcomes**	
Cost of setting up and running the intervention.	• Y-Check documentation and financial records<br>• Interviews with Y-Check staff and staff of the referral facilities.<br>• Programme monitoring data including records of attendance for referrals
Cost per adolescent with a newly diagnosed condition (overall and by condition).	
Cost per adolescent with a newly diagnosed condition who received appropriate on-the-spot care or who completed an appropriate referral within 4 months (overall and by condition).	

For peer review only - http://bmjopen.bmj.com/site/about/guidelines.xhtml

Short-term (4 months) cost-effectiveness: cost per improvement in health or wellbeing (e.g. cost per case addressed or cured), cost per unit improvement in QALYs and per DALY averted.	• Screening tool (self-reported symptoms or conditions, measurements and clinical actions)
**Client outcomes**	
Knowledge about health services and health behaviours.	• Programme monitoring data including records of attendance for referrals<br>• Screening tool (self-reported symptoms or conditions, measurements and clinical actions)<br>• Self-completed evaluation questionnaire
Intentions to adopt healthy behaviours.	
Agency to make decisions about health and wellbeing.	
Perceived social support for behaviour change.	
Health-related risk and protective behaviours.	
Improvement in previously diagnosed health and wellbeing conditions.	
Engagement with health and other services within the past 4 months.	
Self-esteem.	
Self-perceived wellbeing.	
Quality of life.	
Clinical outcomes.	
Educational outcomes (e.g. school attendance).	• Self-completed evaluation questionnaire<br>• School register review
Client-defined outcomes (to be determined).	• Self-completed evaluation questionnaire<br>• Exit interviews

Statistical analysis

All primary analyses will be conducted separately by study city; Cape Coast, Chitungwiza and Mwanza. Where comparable, secondary analyses will be conducted with the data from all three cities combined.

In our study sites, a contemporaneous comparison group is not required since no routine screening is currently taking place, and as a result, assessments at baseline will serve as the counterfactual for internal comparisons. Similarly, since there is no routine screening and treatment provided to adolescents of the target ages in the study population, a before-after comparison is appropriate since it is plausible to assume that reductions in the prevalence of the chronic conditions between the original Y-Check visit and the follow-up at four months will be due to the interventions provided through Y-Check.

We will follow STROBE guidelines for the reporting of cohort studies. Descriptive analyses will be used to compare the community-level and school-level characteristics of the study communities and schools.

For peer review only

For peer review only - http://bmjopen.bmj.com/site/about/guidelines.xhtml

Quantitative programmatic data, including screening test results, services delivered, and referrals made and completed, will be reported by age, sex, and city. The primary outcome is a single proportion which will be presented with a 95% confidence interval for each of the 4 target groups: 10–14-year-old males, 10-14 year-old females, 15-19 year-old males, 15-19 year-old females.

Secondary outcomes which are measured at a single time point will be presented in a similar way to the primary outcome. For outcomes which are measured at two or more time points, a before-after analysis will be conducted comparing differences in measures between the time points. The unit of analysis will be the individual. For clinical outcomes which are measured at two or more time-points, the initial check-up visit (baseline) will give the prevalence of untreated conditions which will represent the counterfactual. The prevalence of conditions at the 4-month follow-up visit will be formally compared to this counterfactual to estimate the short-term effects of the intervention in improving these clinical outcomes. For analysis of outcomes measured at two timepoints we will use mixed effects logistic regression (binary outcomes) or linear regression (continuous outcomes) adjusting for individual-level clustering as a random effect and school/community as a fixed effect. Health service and client determinants of correct management of conditions at 4 months will be analyzed using multivariable regression.

Ethics and Dissemination

Ethics clearance has been received from WHO (WHO/ERC.0003778) and from all country national ethics bodies. Protocol modifications will be shared with the WHO Ethics Review Committee and relevant national ethics boards. Results will be published in at least 3 country-specific peer reviewed journal publications and one multi-country publication. There will also be videos, briefs, webinars and meetings to disseminate results. All data will be placed into an open access repository after deidentification and anonymisation to ensure confidentiality and participant privacy.

For peer review only - http://bmjopen.bmj.com/site/about/guidelines.xhtml

Discussion

Over the last decade, adolescent wellbeing has become a global priority (5). School health is also a growing area of policy interest (36). WHO guidelines on school health services note that along with health promotion, health education, preventive interventions (such as immunizations and mass drug administration), clinical assessment and health services management, health screenings within school learners are one of the key pillars in the delivery of comprehensive school health services (16). Screening programs such as Y-Check provide a unique opportunity to detect easily treatable, high-burden health conditions, refer those requiring medical attention, treatment and care, as well as to advise and encourage adolescents to engage in healthy behaviours.

In a 2015 review, school health services were found to exist in at least 102 countries though their content varied considerably across 16 areas including vaccinations, sexual and reproductive health education, vision screening, nutrition screening, and nutrition health education (37). If all types of screening were combined, they were the second most commonly reported intervention in school health services, second only to immunization. A later systematic review found evidence of routine health check-ups of school age children having been reported in 86 countries worldwide (17). Despite their widespread existence, little quality evidence exists on how to promote good health for adolescents in educational settings (37), and even less for multi-component school health services (38) especially in low- and middle-income countries (39).

Good practices in conducting adolescent health or wellbeing screenings are rarely reported. In 2023, WHO will release new guidance on well-child and well-adolescent visits, which will recommend expanding routine screening tests to also integrate other wellbeing dimensions through a broader evaluation of social risks, emotional state, and individual and family resources delivered with context-specific

For peer review only - http://bmjopen.bmj.com/site/about/guidelines.xhtml

recommendations at key moments during the first two decades of life. The successful implementation of such guidance requires robust measurement of the effectiveness of preventive interventions in adolescence (40).

Evaluation of the Y-Check intervention will incorporate implementation science and effectiveness research. Such hybrid designs have important advantages over conducting separate studies. These include the potential for quicker translation of intervention research findings into programmes, the development and selection of more effective implementation strategies, and more useful information for decision makers (41).

The process evaluation findings will provide guidance for the next stage of the programme and for potential future sustainable and scalable implementation by local health authorities should it prove successful. Data on the short-term changes in clinical and behavioural outcomes will be used as inputs to model both short-term and long-term health and social impacts and as inputs to sample size and power calculations for a third phase of the Y-Check research programme, which plans to undertake a rigorous population level evaluation of the impact of routine check-ups on adolescent health and wellbeing.

Through WHO's advice to member states, findings from the Y-Check study have the potential to shape the delivery of adolescent health check-ups globally including identifying the optimal number, content and delivery for these services. Y-Check will advance the field by providing some of the first rigorous information on the effects of a health screening programme in three African cities, assessing implementation, effectiveness, cost and cost-effectiveness outcomes.

Acknowledgements: The authors thank our Programme Advisors: Prof Simon Gregson, Dr. Sachin Shinde, Prof Fred Binka, Dr. Suzanne Petroni, Prof. Audrey Pettifor, Prof Shane Norris, Prof. James Hargreaves, Ms. Patience Manhibi, Mr Aveneni Mangome. DSMB members are Prof. David Mabey, Dr Andrew Abassa,

For peer review only

For peer review only - http://bmjopen.bmj.com/site/about/guidelines.xhtml

Prof Fred Binka and Dr Nothando Ngwenya. The authors thank adolescent participants in three countries. The support of participating Ministry officials in Ghana, Tanzania and Zimbabwe is gratefully acknowledged.

Authors' contributions: PB, AD, DR conceived and drafted the paper. BW, SK, HAW, VB, FN, FG, MKN, GG, and RAF contributed to writing.

Competing interests*:* PB ad VB are WHO staff members. The opinions expressed are theirs and do not necessarily represent the policies and positions of the World Health Organization. The other authors declared no competing interests.

Funding statement: This work was supported by the Botnar Foundation grant number (RG21-001) and UKRI (MR/T043156/1)

References

1. Bundy DAP, de Silva N, Horton S, Patton GC, Schultz L, Jamison DT. Investment in child and adolescent health and development: key messages from Disease Control Priorities, 3rd Edition. Lancet. 2018;391(10121):687-99.
2. World Bank. World Development Report 2019: The Changing Nature of Work. Washington, DC: World Bank; 2019.
3. Tomlinson M, Ross DA, Bahl R, et al. What will it take for children and adolescents to thrive? The Global Strategy for Women's, Children's, and Adolescents' Health. Lancet Child Adolesc Health. 2019;3(4):208-9.
4. WHO. Global accelerated action for the health of adolescents (AA-HA!): guidance to support country implementation, 2017.
5. WHO. Accelerated Action for the Health of Adolescents (AA-HA! 2.0) Forthcoming. Geneva: World Health Organization; 2023.
6. Kuper H, Monteath-van Dok A, Wing K, et al. The impact of disability on the lives of children; cross-sectional data including 8,900 children with disabilities and 898,834 children without disabilities across 30 countries. PLoS One. 2014;9(9):e107300.
7. WHO. Health for the World's Adolescents: A second chance in the second decade. Geneva: World Health Organization; 2014.
8. Kruk ME, et al. Improving health and social systems for all children in LMICs: structural innovations to deliver high-quality services. Lancet. 2022;399(10337):1830-1844.
9. World Health Organization (WHO). Making health services adolescent friendly: developing national quality standards for adolescent friendly health services, 2012.
10. Patton GC, Sawyer SM, Santelli JS, et al. Our future: a Lancet commission on adolescent health and wellbeing. Lancet. 2016;387(10036):2423-2478.
11. Sanci L. Clinical preventive services for adolescents: facing the challenge of proving "an ounce of prevention is worth a pound of cure". J Adolesc Health. 2011;49(5):450-2.

For peer review only

For peer review only - http://bmjopen.bmj.com/site/about/guidelines.xhtml

12. Harris SK, Aalsma MC, Weitzman ER, Garcia-Huidobro D, Wong C, Hadland SE, Santelli J, Park MJ, Ozer EM. Research on clinical preventive services for adolescents and young adults: where are we and where do we need to go?. Journal of Adolescent Health. 2017 Mar 1;60(3):249-60.
13. Nagata JM, Hathi S, Ferguson BJ, Hindin MJ, Yoshida S, Ross DA. Research priorities for adolescent health in low- and middle-income countries: A mixed-methods synthesis of two separate exercises. J Glob Health. 2018;8(1):doi:10.7189/jogh.08.010501.
14. Bright Futures, American Academy of Pediatrics. Bright Futures. Guidelines for health supervision of infants, children, and adolescents. 2016;4.
15. Royal Australasian College of General Practitioners. Guidelines for Preventive Activities in General Practice, Volume Spec No. 8th ed. East Melbourne: Royal Australasian College of General Practitioners; 2012.
16. WHO. WHO Guideline on School Health Services. 2021.
17. Baltag V, Moran D. Are Routine Health Examinations Fulfilling their Promise for Prevention? Health Behav Policy Rev. 2018;5(6):22.
18. Hoffmann TC, Glasziou PP, Boutron I, et al. Better reporting of interventions: template for intervention description and replication (TIDieR) checklist and guide. BMJ. 2014;348.
19. Chingono RM, Mackworth-Young CRS, Ross DA, et al. Designing routine health check-ups for adolescents in Zimbabwe. J Adolesc Health. 2021;69:940-7.
20. Weobong B, Glozah FN, Abdulai HBT, Koka E, Addae N, Alor S, Kohl K, Banati P, Adongo PB, Ross DA. Reaching adolescents with health services: systematic development of an adolescent health check-ups programme in Ghana (Y-Check, Ghana). (in preparation).
21. Sedekia Y, Mshana G, Nsanya MK, Kohl K, Wambura M, Grosskurth H, Ross DA, Kapiga S. Routine health check-ups for adolescents in Mwanza City, Tanzania: stakeholders' recommendations on its content, venue, and mode of delivery. BMC public health. 2023 May 30;23(1):1015.
22. WHO. Report of Y check Phase 1 to Botnar Foundation, 2020.
23. Green L, Kreuter M. The precede–proceed model. Health promotion planning: an educational approach. 3rd ed. Mountain View (CA): Mayfield Publishing Company; 1999. p. 32-43.
24. McLeroy KR, Bibeau D, Steckler A, Glanz K. An ecological perspective on health promotion programs. Health Educ Q. 1988;15(4):351-377.
25. UNESCO. Net enrollment statistics available at htttp://uis.unesco.org. Downloaded March 18 2023.
26. National Bureau of Statistics. Mwanza Region - Basic Demograhic and Socio-Economic Profile (2012 Census) - National Bureau of Statistics (NBS).; 2016.
27. Ilemela Municipal Council. Ilemela Municipal Council Strategic Plan (2016/2021).; 2017. doi:10.5089/9781513547442.002
28. Zimbabwe National Statistics Agency (ZIMSTAT). Education Statistics Report 2018-2020. Published November 2021. Available from: https://www.zimstat.co.zw/wp-content/uploads/publications/Social/Education/Education_Report_2020.pdf.
29. Zimbabwe Ministry of Primary and Secondary Education (MoPSE). 2021 Primary and Secondary Education Statistics Report. Published May 2022. Available from: http://mopse.co.zw/sites/default/files/public/downloads/EMIS%202021%20Annual%20Education%20Statistics%20Report.pdf.
30. Government of Zimbabwe. Zimbabwe School Health Policy. Harare, Zimbabwe; 2018.
31. Ghana Health Service. Adolescent health service policy and strategy, 2016-2020. Accra, Ghana Health Service. 2016.

For peer review only - http://bmjopen.bmj.com/site/about/guidelines.xhtml

32. Government of Tanzania. Health Sector Strategic Plan July 2021 – June 2026 (HSSP V): Leaving No One Behind. Ministry of Health, Community Development, Gender, Elderly and Children, Dar es Salaam; 2021.
33. WHO. Global standards for quality health-care services for adolescents: a guide to implement a standards-driven approach to improve the quality of health care services for adolescents. 2015 Available from: https://apps.who.int/iris/handle/10665/183935.
34. WHO. Assessing and supporting adolescents' capacity for autonomous decision-making in health care settings: a tool for health-care providers. Geneva: World Health Organization; 2021.
35. Moore GF, Audrey S, Barker M, et al. Process evaluation of complex interventions: Medical Research Council guidance. BMJ. 2015;350:h1258.
36. WHO and UNESCO. Making every school a health-promoting school: global standards and indicators for health-promoting schools and systems. Geneva: World Health Organization and UNESCO; 2021.
37. Baltag V, Pachyna A, Hall J. Global overview of school health services: data from 102 countries. Health Behav Policy Rev. 2015;2(4):268-83.
38. Levinson J, Kohl K, Baltag V, Ross DA. Investigating the effectiveness of school health services delivered by a health provider: a systematic review of systematic reviews. PLoS One. 2019;14(6):e0212603.
39. Montgomery P, Knerr W, Ross DA, Patterson J. The effectiveness and acceptability of comprehensive and multicomponent school health services: A systematic review. J Adolesc Health. 2022;70:192-207.
40. Banati P, Banerjee A, Daelmans B, Baltag V. The need for new models to measure the impact of prevention. J Adolesc Health. 2023;72(1):9-11.
41. Curran GM, Bauer M, Mittman B, Pyne JM, Stetler C. Effectiveness-implementation hybrid designs: combining elements of clinical effectiveness and implementation research to enhance public health impact. Med Care. 2012;50(3):217-26.

Figure 1: Theory of Change for Y-Check, an adolescent health and wellbeing check-up

Figure 2: The Y-Check Intervention package[1]

For peer review only - http://bmjopen.bmj.com/site/about/guidelines.xhtml

Figure 1: Theory of Change for Y-Check, an adolescent health and wellbeing check-up

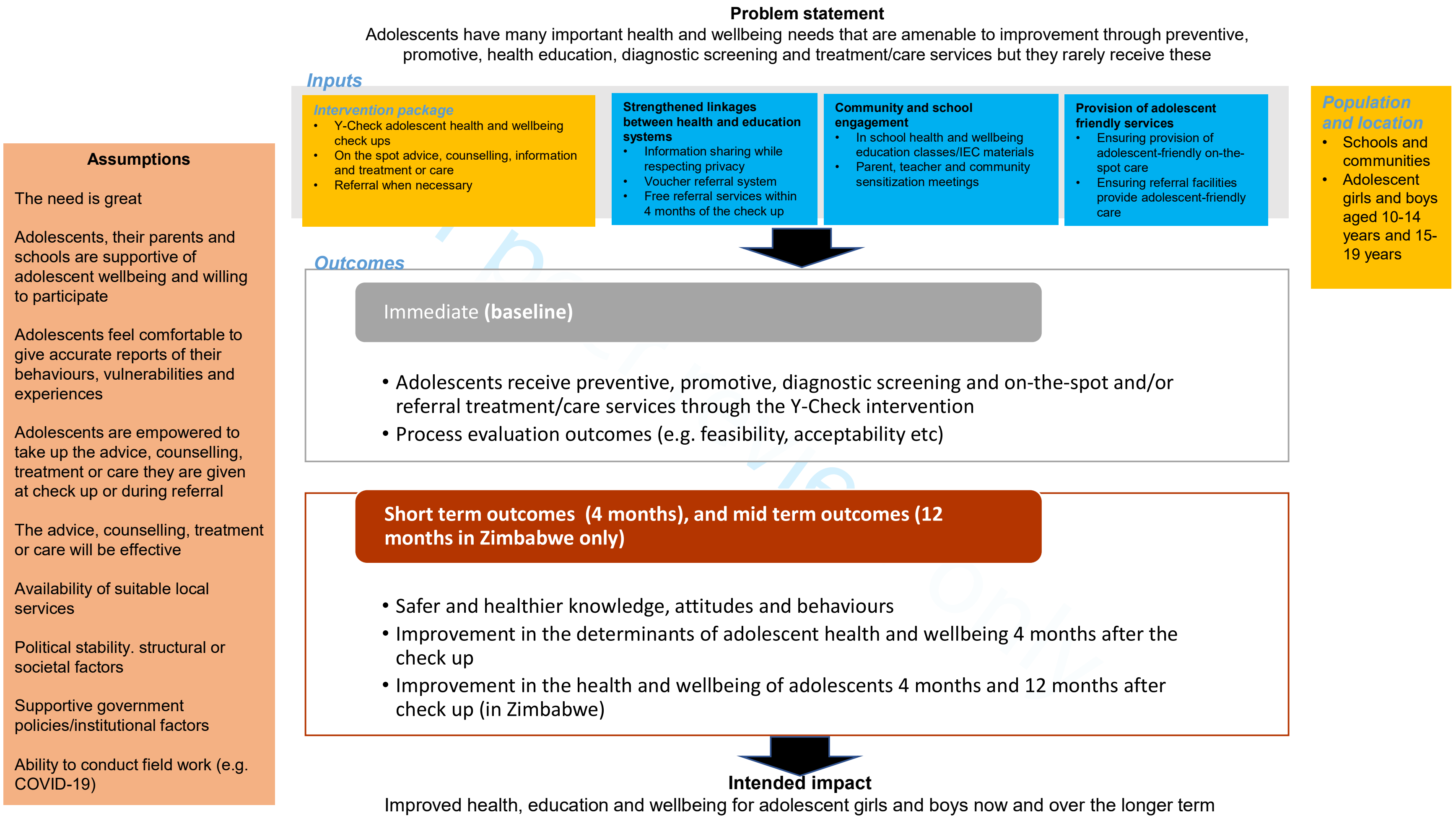

Figure 2: The Y-Check Intervention package[1]

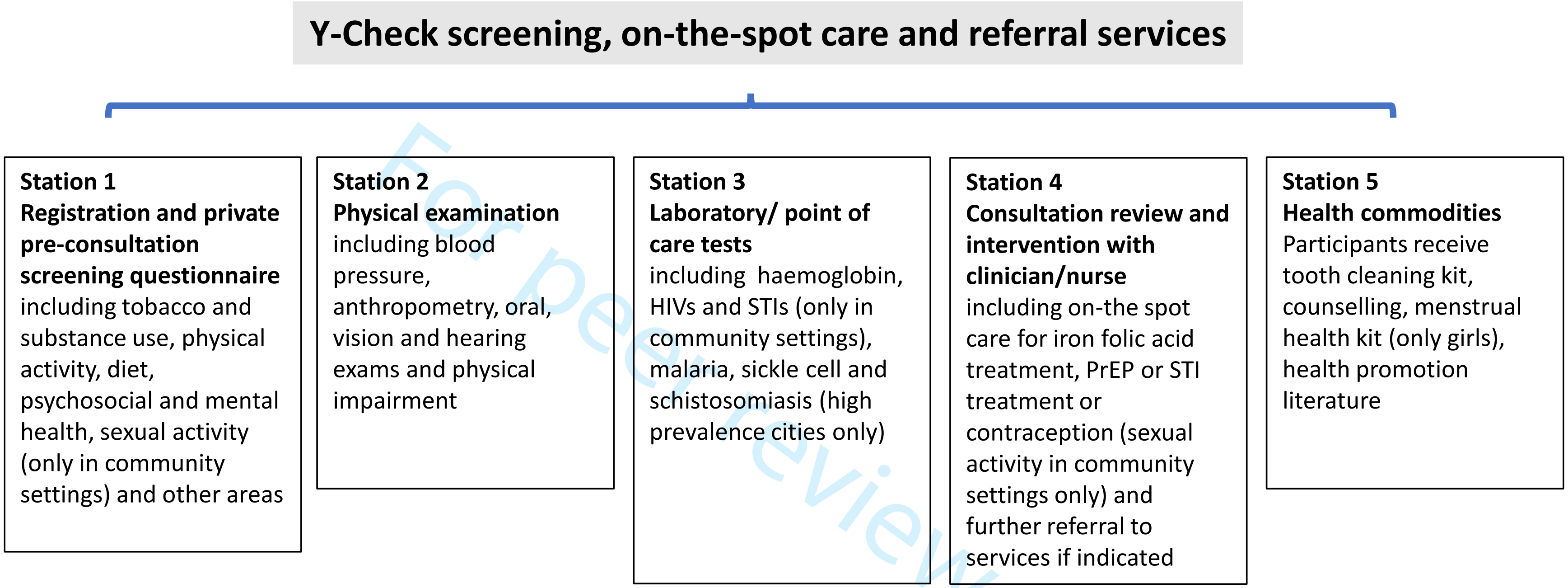

For peer review only

[1] The intervention package may vary according to setting

Table 1: Template for Intervention Description and Replication (TIDieR) checklist describing the Y-Check intervention

Item	Item
**Brief name**	
1	Evaluating the effectiveness of adolescent health check-ups (Y-Check)
**Why?**	
2	Identifying adolescents with poor health, health-compromising behaviours or undiagnosed disability is important for their health and wellbeing, and also for communities and nations<br>Most adolescents only come into contact with health services when they are ill, and services are not always appropriate for their needs<br>Routine health and wellbeing check-up visits for adolescents that screen for multiple preventable and/or treatable conditions and risk behaviours could provide an entry point into services and be highly cost-effective
**What?**	
3	The intervention includes a comprehensive health check-up for priority conditions customized to national and local contexts.<br>Where indicated, Y-Check will provide on-the-spot care and cover all clinical costs associated with referrals to further care provided by the public health system or non-governmental organizations (NGOs).<br>During the check-up, adolescents will receive health promotion information and limited supplies of key health commodities.<br>Clinical costs of services are covered by the study if accessed within 4 months of the check-up.
4	Adolescent-friendly services will be provided, as defined by WHO (2018). Nationally-approved protocols will be applied. Adolescent privacy and confidentiality will be protected.
**Who provided?**	
5	Y-Check teams will be staffed with health professionals trained to provide quality adolescent-friendly health services in line with nationally-approved protocols. Y-Check teams will also be trained in the use of the digital application which will be used for data collection. Public and private not-for-profit care facilities providing referrals will meet national accreditation guidelines.
**How?**	
6	The Y-Check service will take place over a 60-90 minute period face-to-face. Any referrals will only be subsidized by the study if they take place within 4 months.
**Where?**	
7	The Y-Check service will be provided in schools and community venues, in outdoor tents where required.<br>Referrals will be to public or private not-for-profit providers as close as possible to the adolescent´s home. Providers will be vetted by the study team as being able to provide the necessary referral services to national and WHO-recommended standards.
**When and How Much?**	
8	Within the current phase of the study, each adolescent will receive Y-Check once. Within a routine programme the intention would be that the intervention will be delivered twice during adolescence, once when the adolescent is 10-14 years old, and a second time when they are 15-19 years old.
**Tailoring**	
9	The content of the intervention is tailored to local context. The exact set of conditions that will be assessed as part of Y-Check will be adapted based on burden of disease, and availability of local tests and referral services.
**Modifications**	

Item	Item
10	Any modifications will be reported in the article reporting the results of the study.
**How well?**	
11	Intervention fidelity (adherence, integrity, quality) will be evaluated through a process evaluation including youth-friendly health services quality.
12	Intervention fidelity will be reported in the article reporting the results of the study.

For peer review only

STANDARD PROTOCOL ITEMS: RECOMMENDATIONS FOR INTERVENTIONAL TRIALS

SPIRIT 2013 Checklist: Recommended items to address in a clinical trial protocol and related documents*

Section/item	Item No	Description
**Administrative information**		
Title	1	A hybrid evaluation of implementation and short-term cost-effectiveness of Y-Check, an adolescent health and wellbeing check-up programme in three African cities
Trial registration	2a	Registration Protocol ID WHO/ERC.0003778 28/08/2023
	2b	ClinicalTrials.gov Identifier: NCT06090006
Protocol version	3	January 10 2023, Version 4
Funding	4	World Health Organization, Botnar Foundation, UKRI, University of Ghana, Biomedical Research Training Institute Zimbabwe, Mwanza Intervention Trials Unit, Tanzania, London School of Hygiene and Tropical Medicine

For peer review only

Roles and responsibilities 5a

Name of collaborator	Institute and contact details
Prerna Banati (Chief investigator)	Research Scientist<br>World Health Organization<br>Ave Appia 20,<br>1211 Geneva, Switzerland
Valentina Baltag (co-PI)	Team lead<br>AYH, MCA<br>World Health Organization<br>Ave Appia 20,<br>1211 Geneva, Switzerland
Aoife Doyle (PI Zimbabwe)	Associate Professor<br>Biomedical Research and Training Institute, Harare, Zimbabwe<br>and<br>MRC International Statistics & Epidemiology Group<br>Department of Infectious Disease Epidemiology and International Health<br>London School of Hygiene & Tropical Medicine<br>Keppel Street
Rashida Ferrand (co-PI)	Professor<br>Biomedical Research and Training Institute, Harare, Zimbabwe<br>and<br>Department of Clinical Research<br>London School of Hygiene & Tropical Medicine<br>Keppel Street
Saidi Kapiga (PI Tanzania)	Professor<br>Mwanza Intervention Trials Unit,<br>National Institute for Medical Research,<br>Isamilo Road, Mwanza, Tanzania
Helen Weiss (co-PI)	Professor<br>MRC International Statistics & Epidemiology Group<br>Department of Infectious Disease Epidemiology and International Health<br>London School of Hygiene & Tropical Medicine<br>Keppel Street
Benedict Weobong (PI Ghana)	Lecturer<br>School of Public Health, University of Ghana
David Ross (Co-I)	Honorary Professor<br>Institute of Life course studies<br>Stellenbosch University<br>Stellenbosch South Africa
Mussa Nsanya (co-I)	*Research Scientist*,<br>Mwanza Intervention Trials Unit,<br>National Institute for Medical Research,<br>Isamilo Road, Mwanza, Tanzania
Gerry Mshana (co-I)	Principal Research Scientist,<br>Mwanza Intervention Trials Unit, National Institute for Medical Research, Isamilo Road, Mwanza, Tanzania
Yovitha Sedekia (co-I)	Post-doctoral Fellow,<br>Mwanza Intervention Trials Unit, National Institute for Medical Research, Isamilo Road, Mwanza, Tanzania
Chido Dziva Chikwari (co-I)	Biomedical Research and Training Institute, Harare, Zimbabwe<br>and<br>Department of Infectious Disease Epidemiology and International Health<br>London School of Hygiene & Tropical Medicine<br>Keppel Street
Salome Manyau (co-I)	Biomedical Research and Training Institute, Harare, Zimbabwe
Farirai Nzvere (co-I)	Research Manager<br>Biomedical Research and Training Institute, Harare, Zimbabwe
Franklin Glozah (co-I)	Research Scientist<br>School of Public Health, University of Ghana
Philip Adongo (co-I)	Professor<br>School of Public Health, University of Ghana
Eric Koka (co-I)	Research Scientist<br>University of Cape Coast<br>Ghana
Evans Agbeno (co-I)	Research Scientist<br>University of Cape Coast<br>Ghana
Kenneth Addo (Program manager)	Research manager<br>School of Public Health, University of Ghana
Hannah Taylor-Abdulai (co-I)	University of Cape Coast<br>Ghana
Giulia Greco (co-I)	*Assistant Professor in Economics*<br>Department of Global Health and Development<br>London School of Hygiene & Tropical Medicine<br>Keppel Street<br>London WC1E 7HT, UK
Constance Mackworth-Young (co-I)	*Assistant Professor*<br>Biomedical Research and Training Institute, Harare, Zimbabwe<br>and<br>Department of Global Health and Development<br>Faculty of Public Health and Policy<br>London School of Hygiene & Tropical Medicine<br>Keppel Street<br>London WC1E 7HT, UK
Victoria Simms (co-I)	Biomedical Research and Training Institute, Harare, Zimbabwe<br>and<br>MRC International Statistics & Epidemiology Group<br>Department of Infectious Disease Epidemiology and International Health<br>London School of Hygiene & Tropical Medicine<br>Keppel Street<br>London WC1E 7HT, UK

For peer review only

5b	World Health Organization (Study sponsor), Ave Appia 20, 1211 Geneva Switzerland
5c	Study sponsor provides country coordination, oversight and quality control of study design, data collection, management, analysis, and interpretation; writing of the report
5d	**Coordinating center** (WHO) provides country coordination, oversight and quality control of study design, data collection, management, analysis, and interpretation, writing of the report. **Implementing centers** (BRTI, MITU, UGSPH) are responsible for identification, recruitment, data collection and completion of national ethical protocols, along with follow up of study participants and adherence to study protocol. **Programme Advisory Committee (independent)** provides research advise and review of technical and scientific aspects to the research, review and comment on papers; provide recommendations for uptake of results. **Data Safety Monitoring Board (DSMB) (independent)** monitors evidence for harm, assess the impact and relevance of external evidence, asesss whether study follow up should be stopped earlier, assess data quality, monitor recruitment figures and sample size, consider ethical implications, advise on modifications as needed.

For peer review only

Background and rationale	6a	To develop and implement a potentially sustainable adolescent health check-up programme in three African cities (Cape Coast, Ghana; Mwanza, Tanzania; Chitungwiza, Zimbabwe) and evaluate the acceptability, feasibility, short-term effects, and cost-effectiveness of the programme to improve adolescents´ health and well-being. Systematic reviews have identified individual interventions that are effective at improving various aspects of adolescent health and/or well-being. However, most adolescents only come in contact with health services when they are ill, and services are not always appropriate for their needs. This represents a missed opportunity for early detection of health problems and for health promotion, and for the development of beneficial health-seeking behaviours. Early and sustained engagement with health and social services could reap a triple dividend for human development by improving the health and well-being of adolescents, their health and well-being in adulthood and the health and well-being of their future offspring.<br><br>Routine health and well-being check-up visits for adolescents which screen for multiple conditions and risk behaviours, could provide an entry point into services and be highly cost-effective but there is little empirical evidence for their feasibility, acceptability and effects. Many high-income countries have national recommendations related to adolescent health check-ups (largely based on expert opinion). In low- and middle-income settings, preventive health services for adolescents are largely provided in schools, are usually limited to deworming and vaccination campaigns, and do not address other important conditions and risk factors such as nutrition, mental health, or disability. Obtaining evidence on check-ups is a high World Health Organization (WHO) priority for adolescent health research so that they can advise governments on whether or not to start, or to strengthen existing health and well-being check-ups during adolescence and, if so, to develop recommendations for the content and method of delivery of these preventive and promotive contacts.
	6b	In our study sites, a contemporaneous comparison group is not required since no routine screening is currently taking place, and as a result, assessments at baseline will serve as the counterfactual for internal comparisons. Similarly, since there is no routine screening and treatment provided to adolescents of the target ages in the study population, a before-after comparison is appropriate since it is plausible to assume that reductions in the prevalence of the chronic conditions between the original Y-Check visit and the follow-up at four months will be due to the interventions provided through Y-Check.

For peer review only - http://bmjopen.bmj.com/site/about/guidelines.xhtml

Objectives	7	(1) To develop and pilot test a check-up programme for adolescents that screens for important preventable and treatable health conditions using accurate and acceptable screening tests and provides locally accessible effective interventions.<br><br>(2) Through a prospective intervention study in selected schools and communities to: Estimate short-term impacts on adolescent health and wellbeing outcomes: clinical outcomes, health-related knowledge and behaviours, intentions, agency, and perceived social support for behaviour change; engagement with health services, Understand, through process evaluation, the feasibility and fidelity of implementation, the acceptability and uptake, and the influence of context. Estimate the cost-effectiveness of the programme in reducing overall disease burden and improving adolescent wellbeing.<br><br>(3) Obtain information on key parameters needed for the planning of an evaluation study: prevalence of health conditions and behaviours, acceptability of referral, feasibility of following-up programme participants and delivering quality follow-up care, initial estimates of the impact of the programme on longer-term health, educational and wellbeing outcomes based on the short-term implementation and effectiveness outcomes observed in this phase of the research programme, and factors related to the optimal implementation of the Y-Check intervention.<br><br>(4) To refine the programme and its theory of change, and finalise optimal methods for the measurement of the impact of the programme in future studies.
Trial design	8	In this study we propose to conduct implementation science studies to rigorously evaluate the check-ups in real life. We will not conduct a randomized controlled trial (RCT) because the logical next step is to check that it is really feasible and acceptable to deliver the intervention in real life before embarking on a large-scale RCT. As a result, no control group is proposed in this protocol. However, we will include a pilot implementation research study of the intervention that could be tested in the future that will establish the frequency of key health and behavioural outcomes and their short-term impact after 4 months on the health and well-being of the adolescents receiving the intervention through a before-after comparison. We will also use the opportunity to design and pilot test the creation of a Digital Adolescent Health and Well-being Club by recruiting adolescents into the club during the Y-Check screenings.

Methods: Participants, interventions, and outcomes

For peer review only - http://bmjopen.bmj.com/site/about/guidelines.xhtml

Study setting	9	**Ghana**: Cape Coast Metropolis has a total population of 169,894 with three-quarters of the households residing in urban areas. The population in the age group 11-24 years has a literacy rate of about 97%. In 2016 in Cape Coast, 11,233 (68.8%) of 12-14 year-olds were enrolled in junior high schools while 8,407 (91.6%) of 15-17 year-olds were enrolled in senior high schools. Primary and secondary net enrolment rates in 2018 were 84% and 58%, respectively. There are 36 health facilities (26 public and 10 private), including a regional hospital that serves as a secondary referral facility. In the formative phase of Y-Check four communities (Abura, Efutu, Akon, and Kwaprow) within the Cape Coast metropolitan area were involved. Abura and Kwaprow are relatively affluent communities with trading being the main source of livelihood. Akon and Effutu are relatively poorer communities where fishing and farming dominate economic activity, respectively. A total of 172 participants were involved in the study: 16 Key Informants (10 male); 41 younger adolescents (in one school, 11 students were selected (one additional girl in 8th year participated) (mean age: 12 years; 21 female) and their parents; and 37 older adolescents (mean age: 16 years; 22 female) and their parents. For this phase of Y-Check, the study will be conducted in 8 schools within the catchment area of health facilities in all the four communities in the first phase. **Tanzania**: Mwanza is the second largest city in Tanzania after the commercial city of Dar es Salaam. It is located on the southern shores of Lake Victoria in North-western (NW) Tanzania. It has a population of over 900,000 with an annual growth rate of 3%. The primary traditional economic activities include fishing and industrial fish processing for export markets, subsistence agriculture and large and small-scale mining of gold and diamond. Adolescents aged 10 to 19 years make up 24.2% of the total population. As of 2020, gross primary and secondary school enrolment stood at 96.9% and 31.4% respectively. Available public health services include 26 dispensaries, 5 health centres, 2 district hospitals, 1 regional hospital and 1 tertiary/teaching hospital. This study will be conducted in 4 – 6 purposive selected communities and in up to 8 primary schools and 8 secondary schools within the catchment area of health facilities serving the selected communities in the two districts (Nyamagana and Ilemela) within Mwanza city. **Zimbabwe**: Chitungwiza is the third largest city in Zimbabwe, located approximately 25km south of the capital city, Harare, and has a population of about 456 000. The houses are mostly high-density, single story, detached units with small yards that are generally used for growing vegetables. Most of the people work in Harare, as there is little industry in Chitungwiza. There is one tertiary hospital, 4 public primary healthcare facilities, 20 private medical facilities, and 34 government primary schools (all mixed sex). Four communities and four schools were chosen by Chitungwiza stakeholders to take part in the Y-Check Phase 1 formative work in 2019/20 (High schools: Seke High 6, Zengeza High 1; Primary schools: Dungwiza Primary, Chinembiri Primary). Communities and schools were selected to represent the diversity of wards in the town and took into account economic disparities. The selection of the schools and communities for this study will be conducted in collaboration with stakeholders including MoPSE, MoHCC, and the study Youth Advisory Group (YAG) taking into consideration previous participation in the formative work and the location of other ongoing projects. We will aim to work in four distinct communities which are representative of the urban, peri-urban and rural populations of Chitungwiza. Potentially eligible schools must meet the following criteria: Student population of at least 200 learners in Grade 6 or at least 75 learners in Form 5, and located in or close to one of the selected study communities.

For peer review only - http://bmjopen.bmj.com/site/about/guidelines.xhtml

Eligibility criteria	10	To be included in the study, adolescents aged 10-19 must fall into one of the following categories:<br><br>1) Be attending selected classes of Year 5 of primary school in Mwanza (median age 11 years); Grade 5/6 of primary school in Chitungwiza (median age 11 years); or Year 1 of Junior Secondary School in Cape Coast (median age 12 years) OR<br><br>2) Be attending selected classes in Year 3 of Secondary School in Mwanza (median age 17 years), Form 3/4 in Chitungwiza (median age 17 years), or Year 2 of Senior Secondary School in Cape Coast (median age 16 years) OR<br><br>3) Be resident in a selected community during the time of the Y-Check intervention, and be aged 16-19 years<br><br>AND<br><br>4) Have a completed and signed Informed Consent form, or a signed Informed Assent Form and signed Parental/Guardian Informed Consent Form if the adolescent is seen in the community and is below the national age of consent or is seen in a school, irrespective of their age.
Interventions	11a	Y-Check is a novel intervention delivering an adolescent friendly health and wellbeing check-up and where indicated will provide on-the-spot care and/or referral for common conditions on two occasions in adolescence (in young adolescents (10-14 year-olds) – soon after the onset of puberty - and in older adolescents (15-19 year-olds) – when many adolescents become, or are soon to become, sexually active). The intervention will be customised to national and local context. Adolescents will only be screened for conditions that have an accurate, low-cost, acceptable screening test and a locally accessible, effective intervention. The conditions selected for screening will be chosen to reflect the local epidemiological contexts (e.g. screening for malaria will only take place in malaria endemic areas). It will also provide health promotion information and materials to support positive behaviours and healthy lifestyles during adolescence and beyond. Respecting specific requests from the Ministries of Education in all three cities, the study will only include sexual and reproductive health screening and services at the community sites (which only include older adolescents). Locally accessible services will be identified and assessed in terms of their ability to provide the services recommended by local and WHO guidelines, willingness to accept referred adolescents, and the fees charged to the project will be negotiated by the research team for services provided to referred adolescents (where adequate services are not covered by national health insurance schemes, free NGO services or free public health care).

	11b	The consent form identifies the process for withdrawing from the study. If a participant withdraws from the research study and does not consent to further use of their data, we will remove their records from future use to the fullest extent this is possible. As all tests and procedures follow WHO or accredited national guidelines, these will be used as the basis for adaptation, discontinuing or modifying the diagnosis, treatment or care protocols for specific conditions. If urgent care is required during the course of the Y-Check up, they will be supported to attend a local health facility.
	11c	As for any health care, the tests and treatment provided can have side-effects that can be serious or minor. The tests could cause anxiety. The blood test could cause discomfort or a small bruise, as with any other blood test. While the possibility of this happening is low, the informed consent and assent forms will specify these risks clearly to make sure that participants are aware of the possibility. In the unlikely case of an adverse event, the team will be trained to provide care and support, as well as notify the relevant school authorities (for those seen in schools). If urgent care is required, they will be supported to attend a local health facility. Risks will be minimized by explaining the procedures in detail to adolescents during the school sessions, as well as during the process of obtaining informed consent in schools and community venues. Staff will be trained to detect adverse events and a protocol will be in place to ensure action in the rare case that such an event occurs. Table 3 defines the reporting schedule of adverse events. The use of a digital questionnaire is convenient and has the advantage of providing anonymity; however, adolescents may have fears over unauthorised access and trust. There is also a risk to participants of a breach of confidentiality and possible rejection and discrimination by friends and family if they test positive for any of these conditions. The study team will put in place procedures to minimize these risks. The Y-Check team will be trained in good clinical practice, data protection and confidentiality, and counselling for participants testing positive for any previously mentioned conditions.
	11d	There are no prohibitions during the trial period.
Outcomes	12	The primary outcome will be the proportion of those screening positive for at least one condition who receive appropriate on-the-spot care or complete appropriate referral for all identified conditions within 4 months. This will be measured using data collected at the initial check-up visit and through recovery of referral vouchers given to participants to allow them to access referral services for free during the 4-months after the Y-Check screening. Completed referral is defined as attending at least the first referral appointment.<br><br>Secondary implementation outcomes will include the proportion of those screening positive for each condition who receive appropriate on-the-spot care or complete appropriate referral for that condition within 4 months, the yield of previously untreated conditions, clinical outcomes at 4 months among those who had originally screened positive for each condition, and intervention acceptability, adoption, appropriateness, feasibility, fidelity and cost. Secondary effectiveness outcomes will include knowledge about health services and health behaviours, self-reported agency and self-efficacy to make decisions about their health, self-reported health-related risk and protective behaviours, reported engagement with health services, wellbeing, self-esteem and quality of life, clinical outcomes, and educational outcomes, which will be collected within the Y-Check and follow-up visits. The short-term cost-effectiveness of the intervention will be estimated (calculated by a comparison of the costs of the intervention against the primary and secondary outcomes and including short-term changes in self-reported quality of life).

Participant timeline | 13

Activity	2021 Q3	2021 Q4	2022 Q3	2022 Q4	2023 Q1	2023 Q2	2023 Q3	2023 Q4	2024 Q1	2024 Q2	2024 Q3	2024 Q4	2025 Q1	2025 Q2
Inception	*	*												
Intervention development and pilot testing			*	*	*									
Implementation of Y-Check					*	*								
Research cohort recruitment					*	*								
Follow-up at 4 months							*							
Process and economic evaluation									*	*				
Analysis, reporting and dissemination										*	*	*	*	*

*Study timeline for Zimbabwe.

Sample size | 14

The package will be delivered to 2000 adolescents (500 per gender in each age group), however, the primary outcome is only measured among those who screen positive for at least one condition. Within one age group and gender, if 150 (30%) of 500 participants screen positive for at least one condition, and 75% of those who screen positive are correctly managed (n=112; primary outcome), the 95%CI for correct management will be +/-7%.

The sample size also allows us to describe prevalence of individual conditions, and proportion with corrective action taken (secondary outcomes). For example, if 50 of 1000 participants in one age group (5%) screen positive for a given condition, and 75% of these have complete referral, the 95%CI will be 62%-87%, or a 95%CI of 35%-65% if 50% complete referral.

Recruitment | 15

Participants recruited in schools will be reached through whole school sessions, as well as parent sessions. Participants recruited in communities will be reached through door to door community outreach as well as community meetings.

Methods: Assignment of interventions (for controlled trials)

Allocation:

Sequence generation | 16a | NA

Allocation concealment mechanism	16b	NA
Implementation	16c	NA
Blinding (masking)	17a	NA
	17b	NA

Methods: Data collection, management, and analysis

For peer review only

For peer review only - http://bmjopen.bmj.com/site/about/guidelines.xhtml

Data collection methods	18a	The data collection and entry processes will be as detailed in Standard Operating Procedures. We will use Open Data Kit (ODK) and the bespoke Y-Check application for electronic capture of quantitative data into custom-designed forms with built-in range, consistency and discrepancy checks. Answers to sensitive questions will be entered by adolescents themselves to reduce social desirability bias. Field supervisors will scan at least 10% of all forms within 48 hours of data collection to check that there are no obvious problems. They will initiate appropriate actions if there are, such as discussing this at the weekly meetings with the field teams, meeting with individual team members, or arranging specific refresher training. In addition, the data management team will continuously monitor the quality of the data through running frequency distributions of the results for each variable in order to identify unlikely patterns and outliers, and these results will be discussed at the weekly field team meetings. Tablets will be password protected and personal identifiers will be stored in an encrypted format. Service use will also be captured in paper logbooks and registers designed for the specific care and prevention services provided. In each case a log will be kept of the number of people reached, products used, tests performed, etc. Logbook data will be entered on to computers on a weekly basis on pre-designed forms.<br><br>De-identified field notes, team debriefing summaries, and outputs from Participatory Action Research (i.e. pictures from mapping, scoring and ranking activities) will be stored electronically in password-protected files. Audio recordings of discussions and interviews will be transcribed verbatim or summarised in detail and then translated (if necessary) into English for analysis by the research team and stored electronically in password-protected files. Each transcript will also have an accompanying summary form capturing details of the data collection and basic demographic details of the interviewee, as well as any pertinent issues related to the data collection session. Verbatim quotations may be included in reports or publications, but will only report the category of participant, their sex and age. De-identified routine health facility data on the uptake of health services by adolescents before, during and after the Y-Check implementation period will be collected. One of the senior social scientists on each of the three country teams will sit in on an average of at least 5% of the interviews, workshops etc, with a higher proportion early in the data collection to ensure quality and to provide feedback to the field researchers. We will also aim that one of the senior social scientists on each of the three country teams will review all qualitative transcripts and summaries within a fortnight of them having been collected so that problems related to how the interviews, participatory workshops, etc have been conducted or recorded/summarized will be identified and the opportunity taken for mentorship to happen.<br><br>For both quantitative and qualitative data, a major method that we will use to ensure data quality is that the data will be reviewed in real time as they are collected and will not be allowed to accumulate un-reviewed. This should allow problems and inconsistencies to be detected and appropriate steps taken to correct errors early in the data collection process.

	18b	Children in schools will be followed up through continued engagement with the schools. In communities, follow-up will be via phone numbers and addresses provided at the check-up visit. Data on many secondary outcomes will be missing for those lost to follow-up. However, socio-demographic data and primary outcome data will be available for those who are lost to follow-up and can be used to assess potential biases in secondary outcomes due to lost to follow-up.
Data management	19	Data collected off-line on tablet computers will later be synchronised over a local research wi-fi network to the ODK server. Any data transfer over wireless or mobile networks will use Virtual Private Networks or router protected dedicated internet protocol addresses. Data will be fully encrypted to comply with general data protection regulation (GDPR) standards, using a public and private key for encryption and decryption, respectively. All electronic data will be stored in password-protected database systems, with access granted to authorised staff only. When necessary, subsets of the redacted database or other data files may be stored on the PI's or senior staff's laptop to permit analyses during visits or travel. Laptop storage will be encrypted and password-protected to protect data from unauthorised access. Data transferred to LSHTM and/or WHO will be held on Secure Servers utilizing storage systems that provides access controls, integrity verification, encryption, automated daily backup and other functionality to ensure data authenticity and security. While records will not be collected on paper, in some situations (loss of wifi) this might be necessary. Paper records will be stored within the PI or Senior staff's office under lock and key, with access granted only to authorized staff. All data will be stored in multiple secure locations to guard against data loss, and will be stored in date-stamped folders to allow reconstruction of datasets from earlier versions in the unlikely event of a later file becoming corrupted.

view only

For peer review only - http://bmjopen.bmj.com/site/about/guidelines.xhtml

Statistical methods	20a	All primary analyses will be conducted separately by study city; Cape Coast, Chitungwiza and Mwanza. Where comparable, secondary analyses will be conducted with the data from all three cities combined.<br><br>**Validation study**<br>Data will be analysed to calculate the following measures: sensitivity, specificity, positive predictive value, negative predictive value.<br><br>**Programmatic data**<br>Quantitative programmatic data including screening tests results, services delivered, and referrals made and completed will be described according to age, sex, and location.<br><br>**Prospective intervention study**<br>We will follow the STROBE guidelines for the reporting of cohort studies. We will create a flowchart showing the number of communities and schools and the number of participants per community and school at each contact point in the cohort study. We will use descriptive analysis to compare the community-level and school-level characteristics of the study communities and schools.<br><br>The primary outcome is a single proportion which will be presented with a 95% confidence interval for each of the 4 target populations: 10-14 year old male, 10-14 year old female, 15-19 year old male, 15-19 year old female. Secondary outcomes which are measured at a single time point will be presented in a similar way. For outcomes which are measured at two or more time-points, a before-after analysis will be conducted comparing differences in measures between the two time-points. The unit of analysis will be the individual. For clinical outcomes which are measured at two or more time-points, the initial check-up visit (baseline) will give the prevalence of undiagnosed and untreated chronic conditions which will represent the counterfactual. The proportion of undiagnosed and untreated chronic conditions at the 4-month follow-up visit will be formally compared to this counterfactual to estimate the effects of the intervention in improving these clinical outcomes. We will assess health service and client determinants of correct management of conditions at 4 months using multivariable regression. A statistical analysis plan is available.
	20b	All analyses will be disaggregated by age and gender.
	20c	NA

Methods: Monitoring

For peer review only - http://bmjopen.bmj.com/site/about/guidelines.xhtml

Data monitoring	21a	The Data Safety Monitoring Board (DSMB) members will receive and review information on the progress and accruing data of this study. The DSMB should inform the Chair of the PAC if, in their view the results are likely to convince a broad range of clinicians, including those supporting the study and the general clinical community, that, on balance, provision of the Y-Check service is contraindicated for all participants or a particular category of participants, and there was a reasonable expectation that this new evidence would materially influence patient management. The members of the DSMB for this study are:<br>1. Prof Fred Binka, Professor of Clinical Epidemiology, School of Public Health, University of Health and Allied Sciences, Ho, Ghana. Chair, Clinician<br>2. Dr Andrew Abassa, Head of Statistics, MRC/UVRI Uganda Research Unit, Entebbe, Uganda. Statistician<br>3. Prof David Mabey, Professor, London School of Hygiene and Tropical medicine, Clinician<br>4. Dr Nothando Ngwenya, Head of Social Science and Research Ethics, AHRI, South Africa
	21b	The DSMB will be notified in the event of any adverse events. Final decision to terminate the study will rest with the study sponsor.
Harms	22	The DSMB will be notified in the event of any adverse events and make recommendations to the study sponsors.
Auditing	23	NA

Ethics and dissemination

Research ethics approval	24	Ethical clearance has been received from WHO Registration Protocol ID WHO/ERC.0003778 28/08/2023, from London School of Hygiene and Tropical Medicine Approval numbers 26395 and 28312 and from all country national ethics bodies.
Protocol amendments	25	Protocol modifications have been submitted to WHO ethics review committee, LSHTM and national ethics boards and approved by all.

For peer review only

For peer review only - http://bmjopen.bmj.com/site/about/guidelines.xhtml

Consent or assent	26a	To respect the autonomy of adolescents the decision of the minor should prevail. As a result, prior to the visit, adolescents will be shared the assent forms. After adolescents have assented, parents/guardians who would like their adolescent to receive the check-up will be asked to provide written parent/guardian consent. On the day of the check-up visit, a verbal confirmation will be requested from the adolescent. This will be the case for all adolescents taking part in Y-Check in school settings. In community settings, we can expect older and possibly emancipated minors to be participants of the Y-Check service. In Ghana and Tanzania where the age of consent to medical and health-related research is 18 years, adolescents who are not deemed emancipated minors will provide completed parental consent forms and provide written assent before proceeding through the check-up visit. In Zimbabwe where the age of consent to medical and health-related research is 16 years, clients aged 16 years and above who attend the check-ups in the community venues will be allowed to provide written consent for themselves. Emancipated minors will be treated as though they were above the nationally-applicable age of consent. The risks and benefits of the Y-Check intervention will be described to participants and their parents/guardians during the consent/assent process. Adolescents receiving parental consent will be informed that their parents will be notified of test results. Y-Check participants will benefit from early detection of health problems, health promotion, and the promotion of beneficial health-seeking behaviours. However, some conditions such as mental health disorders, HIV and sexually transmitted infections (STIs) are associated with stigma and anxiety. The Y-Check team will be trained in good clinical practice (GCP), data protection and confidentiality, and will provide counselling for participants testing positive for any condition. Furthermore, the protocols and procedures for communicating with adolescents and their families will be carefully developed in collaboration with the three Youth Advisory Groups (YAGs)/Community Advisory Board (CAB) and community stakeholders.
	26b	NA

For peer review only

Confidentiality	27	The protocol for sharing the results of the screening tests will vary according to the perceived seriousness of the condition and/or the seriousness of any stigma associated with the condition, and the age of the participant. A final decision on the classification of conditions as being either more or less serious or seriously stigmatized will be taken in collaboration with adolescents and stakeholders during Year 1. More serious and/or seriously stigmatised conditions may include HIV, STIs, pregnancy, drug use, excessive alcohol use, experiencing violence, suspected epilepsy, severe depressive or severe anxiety symptoms and serious musculoskeletal disorders. Less serious and/or less seriously stigmatised conditions may include anaemia, overweight, underweight, pre-hypertension, hypertension, mild depressive or mild anxiety symptoms, myopia or a hearing disorder. Adolescents who are of majority age or deemed emancipated minors will be given their results directly at the time of the screening. Although they will be encouraged to disclose and discuss the results with their parents, unless they think this would put themselves at risk, the Y-Check team will not disclose their results to their parents unless the adolescent asks for a joint meeting with their parents to discuss them. For all other cases: 1) in the event that the adolescent has no positive test results nor clinically important findings – a letter will be sent home with the child disclosing this information and encouraging continued healthy behaviours. The adolescent will also receive a one-page summary telling them what has been checked for and that nothing serious has been found. They will be encouraged to continue good health-related behaviours. The letter will remind them about health and well-being services available at the school, local health facilities and in the community. 2) in the event that the adolescent is not diagnosed with any condition that requires follow-up or referral (see below) but is diagnosed with a condition that is relatively minor (such as being overweight but not obese) – a letter will be sent home with the child that discloses the results and provides information that promotes and enables self-management, and encourages assistance from their parents. 3) in the event that the adolescent is not diagnosed with any condition that requires referral (see below) but is diagnosed with a condition that is relatively minor but needs follow-up (such as moderate anaemia) – a letter will be sent home with the child that discloses the results, provides information to the parent, and offers the opportunity for the parent to make an appointment with a member of the Y-Check team for further advice regarding the follow-up that may be required (eg. reassessment of the adolescent´s haemoglobin concentration after the three months of iron and folate treatment provided by Y-Check has been completed). 4) in the event that the adolescent is not diagnosed with any condition that requires referral and is stigmatizing (see below) but is diagnosed with a condition that requires referral but is not stigmatizing (such as myopia) – a letter will be sent home with the child that discloses the results and provides information to the parent. The parent will be offered the opportunity to make an appointment to rmeet with a member of the Y-Check team for further advice regarding the condition and the referral. If the parent does not take up the offer of an appointment, the study team will consult with the adolescent, and – if the adolescent gives their permission - school, health or social care staff before deciding on next steps. Potential action would include contacting the parent by phone or through a home visit. 5) in the event that the adolescent is diagnosed with a condition that is considered to be more serious and/or more seriously stigmatizing (such as HIV or epilepsy) – a letter will be sent home with the child that does not disclose results. Parents will be invited and encouraged to make an appointment with the Y-Check nurse/clinician or counsellor to discuss the adolescent's health. The nurse/clinician or counsellor would then meet with the adolescent and the parent together and would explain the condition or suspected condition and what is being recommended. They will ask for the parent´s support to manage the condition and answer any questions and give advice/support as needed. If the parent does not take up the offer of an appointment, the study team will consult with the adolescent, and – if the adolescent gives their permission - school, health or social care staff before deciding on next steps. Potential action would include contacting the parent by phone or through a home visit, and, in emergency situations only, referring the young person to health/social services without the support of the parent.<br><br>For Y-Check in community settings, the adolescent will receive the letter directly and will be encouraged to share it with their parents/guardians. For Y-Check in school settings, the letter will be sent from Y-Check via the school to the adolescent´s parents. All such letters to parents/guardians will be in sealed envelopes addressed to the "Private and Confidential: to the Parent/Guardian of <name of adolescent. In this study, SRH services are only provided in community settings. In Zimbabwe, community services will be provided to 16-19 year-olds, and adolescents who are 16+ in Zimbabwe are able to consent themselves, and therefore no parental disclosure is required, though we will suggest that adolescents inform their parents if they think that they will be able to provide support. In Ghana and Tanzania, older adolescents (16y+) will be able to access Y-Check in community settings. Unless they are deemed emancipated minors (section 7.5), they will require parental consent. The consent and assent forms note that test results will be disclosed to the parent.<br><br>We feel that parents have an important role to play in supporting legal minors, especially after diagnosis of highly stigmatizing conditions, including supporting them emotionally, connecting them with services, treatment or follow-up care. As a result, rather than apply a universal rule for this group, we propose development of a process that respects the best interest of the adolescent, that will enable the clinician to determine the benefits and risks of parental disclosure on a case-by-case basis. We will work during the first part of this study to discuss this with national and international clinicians and researchers as well as global and local ethics boards to determine what is the right course of action.<br><br>**Victims of rape/sexual abuse**<br>In the event that the Y-Check team discovers a case of rape or sexual abuse amongst the participants, the matter shall be referred to the Social Services department. Participants will be told of this legal requirement during the consent procedure so they can decide whether or not they wish to report any such events.

For peer review only - http://bmjopen.bmj.com/site/about/guidelines.xhtml

Declaration of interests	28	The principal investigators have no completing interests.
Access to data	29	All PIs and co-investigators will have access to the data. Once the study is completed, all data will be placed into an open access repository. Data will be deidentified and anonymised to ensure confidentiality and participant privacy
Ancillary and post-trial care	30	NA
Dissemination policy	31a	A publications policy has been developed. Results will be published in at least 3 country specific peer reviewed journal publications and one multicountry publication. There will also be videos, briefs, and webinars to disseminate results.
	31b	Topics suggested for presentation or publication will be circulated to the PIs of the management team, with an abstract, proposed authorship and proposed journal. A writing committee will be formed as described in the publications policy. Disputes regarding authorship will be settled as per the publication policy, and ultimately by the Lead PI if required.
	31c	All data will be placed into an open access repository. Data will be deidentified and anonymised to ensure confidentiality and participant privacy.
**Appendices**		
Informed consent materials	32	Attached in the submission to Ethics Review Committee.
Biological specimens	33	The laboratory tests will be conducted by a trained laboratory technician or laboratory assistant and will include:<br>• Anaemia, using haemoglobin measurement<br>• HIV testing for older adolescents using a HIV oral mucosal transudate test with confirmatory blood testing using Rapid Diagnostic Tests<br>• STI testing for the older adolescents using GeneXpert for Chlamydia trachomatis (CT) and Neisseria gonorrhoeae (NG) and a lateral flow assay for trichomonas vaginalis (TV).<br>• In the two cities where the prevalence of malaria is expected to be high (Mwanza and Cape Coast), all participants will be tested for malaria parasites using the rapid diagnostic test that is recommended by the national malaria control programme.<br>• In the two cities where the prevalence of schistosomiasis is thought to be high (Mwanza and Cape Coast), all participants will be asked to provide both a urine specimen that be tested for *Schistosoma haemotobium* and *Schistosoma mansoni*.<br>No samples will be stored. All samples will be destroyed after testing is completed.

For peer review only - http://bmjopen.bmj.com/site/about/guidelines.xhtml

*It is strongly recommended that this checklist be read in conjunction with the SPIRIT 2013 Explanation & Elaboration for important clarification on the items. Amendments to the protocol should be tracked and dated. The SPIRIT checklist is copyrighted by the SPIRIT Group under the Creative Commons "Attribution-NonCommercial-NoDerivs 3.0 Unported" license.

For peer review only

BMJ Open

An adolescent health and wellbeing check-up programme in three African cities (Y-Check): protocol for a multimethod, prospective, hybrid implementation-effectiveness study

Journal:	*BMJ Open*
Manuscript ID	bmjopen-2023-077533.R2
Article Type:	Protocol
Date Submitted by the Author:	30-Apr-2024
Complete List of Authors:	Banati, Prerna; World Health Organization,<br>Ross, David Anthony; Stellenbosch University<br>Weobong, Benedict; University of Ghana<br>Kapiga, Saidi ; National Institute for Medical Research Mwanza Research Centre; London School of Hygiene & Tropical Medicine<br>Weiss, Helen; London School of Hygiene & Tropical Medicine<br>Baltag , Valentina; World Health Organization<br>Nzvere, Farirai; London School of Hygiene & Tropical Medicine; Biomedical Research and Training Institute<br>Glozah, Franklin; University of Ghana<br>Nsanya, Mussa; National Institute for Medical Research Mwanza Research Centre<br>Greco, Giulia; London School of Hygiene & Tropical Medicine<br>Ferrand, Rashida; London School of Hygiene & Tropical Medicine, Department of Infectious Disease Epidemiology; Biomedical Research and Training Institute<br>Doyle, Aoife; London School of Hygiene & Tropical Medicine; Biomedical Research and Training Institute
**Primary Subject Heading**:	Public health
Secondary Subject Heading:	Evidence based practice
Keywords:	Adolescent, PUBLIC HEALTH, Mass Screening, Schools

SCHOLARONE™
Manuscripts

An adolescent health and wellbeing check-up programme in three African cities (Y-Check): protocol for a multimethod, prospective, hybrid implementation-effectiveness study

Banati P, PhD MPH[1*], Ross DA, MA, BMBCh, MSc, PhD[2] Weobong B, MSc, PhD[3], Kapiga S, MD, MPH, ScD[4,5], Weiss HA PhD MSc[6], Baltag V MD PhD[1], Nzvere F[5,7], Glozah F, MPhil, PhD[3], Nsanya MK, MD, MSc[4], Greco G, PhD MSc[5], Ferrand RA, PhD MBBS[5,7], and Doyle AM, PhD[6,7] for the Y-Check Research Programme Team[8]

[1] Adolescent and Young Adult Health (AYH) Unit, Maternal, Newborn, Child and Adolescent Health and Ageing (MCA) Department, World Health Organization (WHO), Ave Appia 20, 1211 Geneva 27, Switzerland
[2] Institute for Life Course Health Research, Stellenbosch University, Tygerberg, South Africa
[3] University of Ghana School of Public Health, College of Health Sciences (UGSPH), Ghana
[4] Mwanza Intervention Trials Unit, National Institute for Medical Research Mwanza Research Centre (MITU/NIMR), Isamilo, Mwanza, United Republic of Tanzania
[5] Department of Infectious Disease Epidemiology, London School of Hygiene and Tropical Medicine, Keppel St, London WC1E 7HT, UK
[6] MRC International Statistics and Epidemiology Group, London School of Hygiene & Tropical Medicine, Keppel St, London WC1E 7HT, UK
[7] Biomedical Research and Training Institute (BRTI), 10 Seagrave Road, Avondale, Harare, Zimbabwe
[8] The Y-Check Research Programme Team in alphabetical order includes Prof. Philip B. Adongo, MA, PhD, Department of Social and Behavioural Sciences, School of Public Health, University of Ghana; Mr. Kenneth. S. Adde, MPhil, Department of Population and Health, University of Cape Coast; Prof Evans Agbeno, FGCS, MPH, MBCHB, Department of Obstetrics & Gynaecology, University of Cape Coast; Prof. Patricia Akweongo, MA, PhD, Department of Health Policy Planning and Management, School of Public Health, University of Ghana; Ms Tsitsi Bandason MSc, The Health Research Unit Zimbabwe/ Biomedical Research and Training Institute; Dr. Sarah Bernays, PhD MA, Global Health and Development, London School of Hygiene and Tropical Medicine and School of Public Health, University of Sydney; Dr Rudo Chingono PhD, The Health Research Unit Zimbabwe/ Biomedical Research and Training Institute Dr Chido Dziva Chikwari PhD, MSc, BSc, The Health Research Unit Zimbabwe/ Biomedical Research and Training Institute and MRC International Statistics & Epidemiology Group, Department of Infectious Disease Epidemiology, London School of Hygiene & Tropical Medicine, London, UK; Ms Ethel Dauya MPH, The Health Research Unit Zimbabwe/ Biomedical Research and Training Institute; Dr. S. Derry, PhD, Department of Bio-Statistics, School of Public Health, University of Ghana; Dr. Eric Koka, MPhil, PhD, Department of Sociology and Anthropology, University of Cape Coast; Dr. Constance Mackworth-Young, PhD MSc, Global Health and Development, London School of Hygiene and Tropical Medicine, Ms. Salome Manyau MSc, The Health Research Unit Zimbabwe/ Biomedical Research and Training Institute; Mr. Bernard A. Owusu, MPhil, Department of Population and Health, University of Cape Coast; Dr. Yovitha Sedekia, MPHDC, PhD, Mwanza Intervention Trials Unit (MITU)/National Institute for Medical Research (NIMR); Dr. Hannah Taylor-Abdulai, MPH, MPhil, PhD, Department of Physician Assistant Studies, University of Cape Coast; ; Dr. Mandi Tembo, PhD, The Health Research Unit Zimbabwe/ Biomedical Research and Training Institute and Department of Global Health and Development, Faculty of Public Health and Policy, London School of Hygiene and Tropical Medicine, London, United Kingdom.

* Corresponding Author

Email addresses

Banati P (banatip@who.int); Ross DA (dross.rbridge@gmail.com); Weobong B (bweobong@ug.edu.gh); Kapiga S (Saidi.Kapiga@lshtm.ac.uk); Weiss HA (Helen.Weiss@lshtm.ac.uk); Baltag V (baltagv@who.int); Nzvere F (Farrie.Nzvere@lshtm.ac.uk); Glozah F (fglozah@ug.edu.gh); Nsanya MK (mussa.nsanya@mitu.or.tz); Greco G (Giulia.Greco@lshtm.ac.uk); Ferrand RA (rashida.ferrand@lshtm.ac.uk); Doyle AM (aoife.doyle@lshtm.ac.uk)

For peer review only

Article Summary

Abstract

Background: During adolescence, behaviours are initiated that will have substantial impacts on the individual's short- and long-term health and wellbeing. However, adolescents rarely have regular contact with health services, and available services are not always appropriate for their needs. We co-developed with adolescents a health and wellbeing check-up programme (Y-Check). This paper describes the methods to evaluate the feasibility, acceptability, short-term effects, and cost-effectiveness of Y-Check in three African cities.

Method: This is a multi-country prospective intervention study, with a mixed-method process evaluation. The intervention involves screening, on-the-spot care and referral of adolescents through health and wellbeing check-up visits. In each city, 2000 adolescents will be recruited in schools or community venues. Adolescents will be followed-up at 4 months. The study will assess the effects of Y-Check on knowledge and behaviours, as well as clinical outcomes and costs. Process and economic evaluations will investigate acceptability, feasibility, uptake, fidelity and cost effectiveness.

Ethics and Dissemination: Approval has been received from the WHO (WHO/ERC Protocol ID Number ERC.0003778); Ghana Health Service (Protocol ID number GHS-ERC: 027/07/22), the United Republic of Tanzania National Institute for Medical Research (Clearance No. NIMR/HQ/R.8a/Vol.IX/4199), the Medical Research Council of Zimbabwe (Approval number MRCZ/A/2766), and the LSHTM (Approval numbers 26395 and 28312). The trial registration number is NCT06090006. Consent and disclosure are addressed in the paper. Results will be published in 3 country-specific peer reviewed journal publications, and one multi-country publication; and disseminated through videos, briefs, and webinars. Data will be placed into an open access repository. Data will be deidentified and anonymized.

For peer review only

For peer review only - http://bmjopen.bmj.com/site/about/guidelines.xhtml

71 **Keywords**: Adolescent, health, wellbeing, check-ups, screening, implementation research, effectiveness,
72 cost-effectiveness

For peer review only - http://bmjopen.bmj.com/site/about/guidelines.xhtml

Additional information

Strengths and limitations of the methodology:

- **Strength**: This study will utilize existing health care infrastructure in low- and middle-income country settings, assessing real world implementation situations and therefore it will be relatively straightforward to directly apply the findings to programs.
- **Strength**: This is a relatively large study of 6000 adolescents in 3 countries. The study takes the views of young people centrally into the design of the intervention.
- **Limitation**: Although the primary outcome is an implementation science / programmatic outcome, the effectiveness data is based on pre-post comparison.
- **Limitation**: This study will have limited ability to assess sustainability of effects over the longer term as the follow up period is 4 months
- **Limitation**: This study is operating in three African cities which may limit generalizability to rural areas.

Manuscript Wordcount: 5023

For peer review only - http://bmjopen.bmj.com/site/about/guidelines.xhtml

Background

To unlock human potential and accelerate progress towards achieving the Sustainable Development Goals (SDGs), it is essential to improve the health and wellbeing of adolescents (10-19 years) (1). Health is an essential component of human capital (2), yet adolescent investments have focused primarily on either health or education services with little attention to synergies between these (3). Research investments in the first 1000 days of life have dramatically outweighed investments in the subsequent 7000 days, leaving an evidence gap on how to develop and sustain human potential through adolescence and early adulthood (4).

Among adolescents in low- and middle-income countries (LMICs), HIV/AIDS, road injury, diarrheal diseases, self-harm, iron-deficiency anemia and skin diseases are among the top causes of morbidity and mortality (5, 6, 7). Identifying adolescents with poor health, health-compromising behaviours or undiagnosed disability is important given (a) the growing number of adolescents and their low frequency of regular contacts with health services (8) (b) the high proportion of the total global burden of disease that occurs in adolescence and (c) the fact that many key health conditions (e.g. mental health disorders) and behaviours (e.g. tobacco and alcohol use, unhealthy diet, low physical activity, risky sexual behaviours) that predispose to preventable serious conditions in later life start in adolescence (d) the negative impact of poor health on educational attainment and employability and other transitions to healthy adulthood, and (e) gender-related vulnerabilities, including violence, abuse, unintentional injury, sexual and reproductive health (SRH) and gendered mental health outcomes which may emerge or be exacerbated during this period of life, setting negative trajectories to lifetime and intergenerational health and wellbeing (4).

Systematic reviews have identified individual interventions that are effective at improving various aspects of adolescent health and/or wellbeing (4) However, most adolescents only come into contact with health

For peer review only - http://bmjopen.bmj.com/site/about/guidelines.xhtml

services when they are ill, and services are not always appropriate for their needs (9). This represents a missed opportunity for early detection of health problems, for health promotion, and for the development of health-seeking behaviours. Early and sustained engagement with health and social services could reap a triple dividend for human development by improving the health and wellbeing of adolescents, their health and wellbeing in adulthood and the health and wellbeing of their future offspring (2, 4, 10)

Routine health and wellbeing check-up visits for adolescents that screen for multiple conditions and risk behaviours could provide an entry point into services and be highly cost-effective (11, 12). Obtaining evidence on the optimum content, delivery, effectiveness and cost of check-ups is a high priority for adolescent health research so that governments can be informed by the evidence on how to initiate or strengthen existing health and wellbeing check-ups during adolescence (13). Many high-income countries have national recommendations related to adolescent health check-ups, which have been largely based on expert opinion (14,15). In LMICs, if provided at all, preventive and promotive health services for adolescents are largely provided in schools and are usually limited to deworming and vaccination campaigns. They do not usually address other key conditions and risk factors such as nutrition, mental health, SRH or disability (16, 17). If a system-wide approach to check-ups exists in adolescence, in LMICs it is often limited to a screening activity without other components such as brief intervention or anticipatory guidance (17).

This paper describes the protocol for the Y-Check: Evaluating the effects of adolescent health check-ups study, a prospective hybrid implementation-effectiveness study evaluating the feasibility, acceptability, short-term effects, costs and cost-effectiveness of the Y-Check intervention in three African cities. This study has received approval from the World Health Organization (WHO/ERC Protocol ID Number ERC.0003778); Ghana Health Service (Protocol ID number GHS-ERC: 027/07/22), the United Republic of Tanzania National Institute for Medical Research (Clearance No. NIMR/HQ/R.8a/Vol.IX/4199), the

For peer review only

For peer review only - http://bmjopen.bmj.com/site/about/guidelines.xhtml

Medical Research Council of Zimbabwe (Approval number MRCZ/A/2766), and the London School of Hygiene and Tropical Medicine (Approval numbers 26395 and 28312) .

The Y-Check intervention

Y-Check is a novel intervention delivering a health and wellbeing check-up and where indicated will provide on-the-spot care and/or referral for common conditions on two occasions in adolescence (in young adolescents (10-14 year-olds) – soon after the onset of puberty - and in older adolescents (15-19 year-olds) – when many adolescents become, or are soon to become, sexually active). It will also provide health promotion information and materials to support positive behaviours and healthy lifestyles during adolescence and beyond. The intention is that in the context of a future routinely-delivered programme, every adolescent will have two guaranteed contacts with the health care system. Adolescents will only be screened for conditions that have an accurate, low-cost, acceptable screening test and a locally accessible, effective intervention. The conditions selected for screening will be chosen to reflect the local epidemiological contexts (e.g. screening for malaria will only take place in malaria endemic areas). Respecting specific requests from the Ministries of Education in all three cities, the study will only include sexual and reproductive health (SRH) screening and services at the community sites (which only include older adolescents).

Figures 1 and 2 present the Theory of Change and description of the intervention. Table 1 applies the TIDieR checklist (18) to describe details of the intervention.

Locally accessible services will be identified and assessed in terms of their ability to provide the services recommended by local and WHO guidelines, willingness to accept referred adolescents, and the fees charged to the project will be negotiated by the research team for services provided to referred

For peer review only - http://bmjopen.bmj.com/site/about/guidelines.xhtml

159 adolescents (where adequate services are not covered by national health insurance schemes, free NGO

160 services or free public health care).

161 **Insert Figures 1 and 2**

For peer review only

For peer review only - http://bmjopen.bmj.com/site/about/guidelines.xhtml

162 **Table 1: Template for Intervention Description and Replication (TIDieR) checklist describing the Y-Check**
163 **intervention**

Item	Item
**Brief name**	
1	Evaluating the effectiveness of adolescent health check-ups (Y-Check)
**Why?**	
2	Identifying adolescents with poor health, health-compromising behaviours or undiagnosed disability is important for their health and wellbeing, and also for communities and nations<br>Most adolescents only come into contact with health services when they are ill, and services are not always appropriate for their needs<br>Routine health and wellbeing check-up visits for adolescents that screen for multiple preventable and/or treatable conditions and risk behaviours could provide an entry point into services and be highly cost-effective
**What?**	
3	The intervention includes a comprehensive health check-up for priority conditions customized to national and local contexts.<br>Where indicated, Y-Check will provide on-the-spot care and cover all clinical costs associated with referrals to further care provided by the public health system or non-governmental organizations (NGOs).<br>During the check-up, adolescents will receive health promotion information and limited supplies of key health commodities.<br>Clinical costs of services are covered by the study if accessed within 4 months of the check-up.
4	Adolescent-friendly services will be provided, as defined by WHO (2018). Nationally-approved protocols will be applied. Adolescent privacy and confidentiality will be protected.
**Who provided?**	
5	Y-Check teams will be staffed with health professionals trained to provide quality adolescent-friendly health services in line with nationally-approved protocols. Y-Check teams will also be trained in the use of the digital application which will be used for data collection. Public and private not-for-profit care facilities providing referrals will meet national accreditation guidelines.
**How?**	
6	The Y-Check service will take place over a 60-90 minute period face-to-face. Any referrals will only be subsidized by the study if they take place within 4 months.
**Where?**	
7	The Y-Check service will be provided in schools and community venues, in outdoor tents where required.<br>Referrals will be to public or private not-for-profit providers as close as possible to the adolescent´s home. Providers will be vetted by the study team as being able to provide the necessary referral services to national and WHO-recommended standards.
**When and How Much?**	
8	Within the current phase of the study, each adolescent will receive Y-Check once. Within a routine programme the intention would be that the intervention will be delivered twice during adolescence, once when the adolescent is 10-14 years old, and a second time when they are 15-19 years old.
**Tailoring**	
9	The content of the intervention is tailored to local context. The exact set of conditions that will be assessed as part of Y-Check will be adapted based on burden of disease, and availability of local tests and referral services.
**Modifications**	

For peer review only - http://bmjopen.bmj.com/site/about/guidelines.xhtml

Item	Item
10	Any modifications will be reported in the article reporting the results of the study.
**How well?**	
11	Intervention fidelity (adherence, integrity, quality) will be evaluated through a process evaluation including youth-friendly health services quality.
12	Intervention fidelity will be reported in the article reporting the results of the study.

Methods/Design

Aims

The aim of the study is to develop and implement in three African cities a potentially sustainable adolescent health check-up programme, and evaluate the acceptability, feasibility, short-term effects, and cost-effectiveness of the programme to improve health and wellbeing. The study was launched in September 2021 and will run until June 2025.

Objectives

(1) To develop and pilot test a check-up programme for adolescents that screens for important preventable and treatable health conditions using accurate and acceptable screening tests and provides locally accessible effective interventions.

(2) Through a prospective intervention study in selected schools and communities to:

- Estimate short-term impacts on adolescent health and wellbeing outcomes: clinical outcomes, health-related knowledge and behaviours, intentions, agency, and perceived social support for behaviour change; engagement with health services.
- Understand, through process evaluation, the feasibility and fidelity of implementation, the acceptability and uptake, and the influence of context.

For peer review only

- Estimate the cost-effectiveness of the programme in reducing overall disease burden and improving adolescent wellbeing

(3) Obtain information on key parameters needed for the planning of an evaluation study: prevalence of health conditions and behaviours, acceptability of referral, feasibility of following-up programme participants and delivering quality follow-up care, initial estimates of the impact of the programme on longer-term health, educational and wellbeing outcomes based on the short-term implementation and effectiveness outcomes observed in this phase of the research programme, and factors related to the optimal implementation of the Y-Check intervention.

(4) To refine the programme and its theory of change, and finalise optimal methods for the measurement of the impact of the programme in future studies.

Patient and public involvement

The intervention was designed following formative research conducted in three African countries between 2019 and 2020 (19, 20, 21). This formative research revealed that the proposed adolescent health and wellbeing check-ups are likely to be feasible to implement and acceptable to stakeholders in Ghana, Tanzania and Zimbabwe, and are likely to meet the perceived needs of key stakeholders including adolescents, their parents, and key policy makers in the health and education sectors (22). Further, we showed that the programme is likely to produce a substantial yield of important, previously untreated, treatable conditions. Human-centered design techniques were used alongside desk review to define elements of objective and subjective importance to the health and wellbeing of adolescents, identify facilitators and barriers to adolescent health seeking, preferences for delivery of routine health check-ups, and potential effects of interventions to select the content and method of delivery of the Y-Check intervention. Interviews and participatory workshops with adolescents, parents of adolescents and key stakeholders from the ministries of health and education, non-governmental organizations, healthcare

For peer review only

For peer review only - http://bmjopen.bmj.com/site/about/guidelines.xhtml

workers and teachers found that there was overall support for the introduction of routine health check-ups (19, 20, 21). To navigate potential barriers, stakeholders suggested clear messaging, awareness building, and sensitization campaigns to overcome disinterest in preventative healthcare and, in some contexts, mitigate cultural or religious messaging against healthcare engagement (19).

Theory of Change

We hypothesise that a routine health and wellbeing check-up visit for adolescents that screens for multiple conditions and risk behaviours will have an immediate and long-term positive impact on health and wellbeing outcomes (Figure 1).

Health seeking and promotion behaviours among adolescents operate in complex environments and across ecological levels (10), with determinants at individual, interpersonal institutional/organizational, community and public policy levels. Drawing from the health promotion literature (23, 24), the Theory of Change for Y-Check (Figure 1) draws on thinking that recognizes pre-disposing, enabling and reinforcing factors as capacities to be strengthened in order to achieve adolescent wellbeing at the individual level; that responsive parenting can support adolescents to meet their own health and wellbeing goals; that systems-based approaches (including stronger linkages between health and education systems) can improve outcomes for adolescents, especially reaching the most vulnerable and those in need; and that an enabling environment (especially in schools and communities) can support adolescents to take action towards improving their health.

Study setting

Our study will be undertaken in three African cities: Cape Coast in Ghana, Mwanza in Tanzania and Chitungwiza in Zimbabwe. These cities are described in Table 2.

For peer review only - http://bmjopen.bmj.com/site/about/guidelines.xhtml

Table 2: The study cities, schools and communities

**Cape Coast, Ghana**	**Mwanza, Tanzania**	**Chitungwiza, Zimbabwe**
Cape Coast Metropolis is located on the coast of Ghana, 150kms west of the capital city, Accra. It has a population of 169,894 with three-quarters of the households residing in urban areas.<br><br>Literacy in 11-24 year-olds is about 97%. In 2016, 11,233 (68.8%) of 12-14 year-olds were enrolled in junior high schools while 8,407 (91.6%) of 15-17 year-olds were enrolled in senior high schools. For Ghana as a whole, primary and secondary net enrollment rates in 2019 were 86% and 57%, respectively (25)<br><br>There are 36 health facilities (26 public and 10 private) in the metropolitan area, including a regional hospital that serves as a secondary referral facility.<br><br>The study will be conducted in 8 schools and local community venues in four communities that include two relatively affluent communities with trading being the main source of livelihood and two relatively poorer communities where fishing and farming dominate, respectively.	Mwanza is located on the southern shores of Lake Victoria in North-Western Tanzania and is the second largest city in Tanzania with a population of over 900,000 and an annual growth rate of 3% (26). Economic activities in Mwanza include fishing and fish processing, subsistence agriculture and support services to nearby gold and diamond mines.<br><br>Adolescents make up 24.2% of the population of the city (Tanzania National Bureau of Statistics, 2016). As of 2020/21, the primary and secondary school net enrollment rates were 82% and 39%, respectively (26)<br><br>Available public health services include 26 dispensaries, 5 health centres, 2 district hospitals, 1 regional hospital and 1 tertiary/teaching hospital (26, 27).<br><br>The study will be conducted in 4–6 purposively-selected communities and in up to 8 primary schools and 8 secondary schools within the catchment area of health facilities serving the selected communities in the two districts within Mwanza city.	Chitungwiza is the third largest city in Zimbabwe, located approximately 25km south of the capital city, Harare. It has a population of about 456,000 (28). The houses are mostly high-density, single-story, detached units with small yards that are generally used for growing vegetables. Most of the people work in Harare, as there is little industry in Chitungwiza itself.<br><br>Zimbabwe has a school-going population (8-18 years) of approximately 4.3 million (29). Net primary enrollment rate across Zimbabwe is 94%; net secondary enrollment rate is 54% (28)<br><br>In Chitungwiza, there is one tertiary hospital, 4 public primary healthcare facilities, 20 private medical facilities, 30 government primary schools, and 13 government secondary schools (all mixed sex).<br><br>The study will be conducted in four distinct communities which are representative of the urban, peri-urban and rural populations of Chitungwiza. Eligible schools must have a student population of at least 200 learners in Grade 6 or at least 75 learners in Form 5; and be located in or close to one of the selected study communities.

Study design

In this prospective hybrid implementation-effectiveness study, 2000 adolescents per city who receive the Y-Check intervention will be followed up at 4-months, and at 12-months (Zimbabwe only).

Stakeholder engagement

In each city, the research study is undertaken in partnership with both the national and municipal Ministries of Health and Education. Each country has a policy framework that provides encouragement for the introduction of health and nutrition education and promotion among adolescents, including screening for communicable and non-communicable diseases, immunization, growth monitoring and assessments and nutritional services (30-32).

For peer review only - http://bmjopen.bmj.com/site/about/guidelines.xhtml

This study will build on stakeholder engagement, the process for which was established in each research setting during the formative phase. In each city, a Community Advisory Committee (CAC) comprising key community leaders and stakeholders will be reinforced or set up to facilitate input from, and feedback to, participating communities and a Youth Advisory Group (YAG) will provide a forum for adolescents to input into the programme. The YAG will meet with research staff at least 4 times per year, be active participants in programme design and dissemination workshops, and help to ensure that the programme meets the needs of adolescents. Community engagement will be an ongoing process through regular contacts with the CAC, the YAG and other stakeholders, such as teachers, health workers, Community Based Organizations (CBOs), Non-Governmental Organizations (NGOs), and religious leaders. In addition, a key aspect for building confidence within communities is the knowledge that the study has the support of the government.

Intervention development and pilot testing

Prior to implementation, preparatory activities will include community engagement, participatory co-design, negotiating referral arrangements and pre-testing of screening tools, procedures and referral protocols. Pilot studies in each setting will provide initial estimates of the frequency of health and behavioural outcomes, and help to refine the intervention model.

Pilot testing will involve the implementation of the screening tools and procedures with approximately 200 adolescents in each of the three cities with revisions and repeat pilot testing where required. Adolescents who participate in the pilot study will be excluded from the main study if the procedures change following the pilot. There will be an opportunity for young people and stakeholders to suggest additional client-centered outcomes that may reflect some of their priority concerns or intentions that should be captured.

For peer review only - http://bmjopen.bmj.com/site/about/guidelines.xhtml

Intervention implementation

The intervention will be delivered over a period of 2-6 months in each of the settings. The follow-up visits will take place at the same school or community setting as the initial check-up. In addition to covering all clinical costs, the equivalent of USD 5 will be given to each participant who attends the follow-up to cover any transport costs that they might have incurred. Additionally, health and hygiene related items will also be provided for adolescents to take home, including tooth cleaning kit (toothbrush and toothpaste), fruit, bottle of water, two pairs of underpants, pack of reusable sanitary pads (girls only)

Composition and training of Y-Check team

The Y-Check team will be trained to deliver adolescent-responsive and age-appropriate services according to national and WHO guidelines, recognizing also the needs for privacy and confidentiality (33). This includes providing services that are attractive to adolescents, meet their needs comfortably and responsively, and that are attentive to their privacy. These principles and approaches will be embedded into each part of the Y-Check intervention. Visual and auditory privacy will be prioritized, through the use of separate tents, rooms or screens. Health workers will employ standard gowning and draping for clinical procedures.

For infection prevention and control (IPC), all study procedures including interviews, physical examinations and blood tests will take place in well-aerated tents or outdoors, and will follow relevant nationally-approved protocols for all staff and participants.

The Y-Check team will be trained in good clinical practice, data protection and confidentiality, and clinical staff will be trained in counselling for participants testing positive for any of the conditions being screened for within Y-Check as well as in general counselling skills.

For peer review only - http://bmjopen.bmj.com/site/about/guidelines.xhtml

Inclusion and exclusion criteria

To be included in the study, adolescents aged 10-19 years must fall into one of the first three categories below and fulfil category 4.

1) Be attending selected classes of Year 5 of primary school in Mwanza (median age 11 years); Grade 5/6 of primary school in Chitungwiza (median age 11 years); or Year 1 of Junior Secondary School in Cape Coast (median age 12 years) OR

2) Be attending selected classes in Year 3 of Secondary School in Mwanza (median age 17 years), Form 3/4 in Chitungwiza (median age 17 years), or Year 2 of Senior Secondary School in Cape Coast (median age 16 years) OR

3) Be resident in a selected community during the time of the Y-Check intervention, and be aged 16-19 years

AND

4) Have a completed and signed Informed Consent form, or a signed Informed Assent Form and signed Parental/Guardian Informed Consent Form if the adolescent is seen in the community and is below the national age of consent or is seen in a school, irrespective of their age.

Consent and Assent procedures

Before the visit of the implementation team, information on the Y-Check programme will be distributed to parents/guardians through the schools and to community members through an active communication campaign in collaboration with the CAC and the YAG. School and community meetings will allow parents and community members to ask questions about the programme and give their feedback.

For peer review only - http://bmjopen.bmj.com/site/about/guidelines.xhtml

In schools, adolescents will have a short introductory meeting with a member of the Y-Check team typically in a class or group setting. Parents meetings will then be held in each of the schools, to which all the parents and guardians of eligible learners will be invited. During these sessions, information will be provided about the study, its objectives and procedures, possible risks and procedures that will be used to maintain confidentiality. These meetings will provide an opportunity for the adolescents, parents and guardians of eligible adolescents to learn more about the Y-Check intervention and the research linked to it and to have their questions answered.

No participants will be screened, receive care or be counselled or interviewed without their informed consent (community participants who are above the national age of consent), or, for minors, their assent and parental consent, unless they are determined to be emancipated minors (34). Following advice from Ministries of Education in all three countries, all adolescents seen in schools will be considered to be minors and require parental consent, irrespective of their age.

Minor adolescents' assent will be ascertained and documented in an assent form. Parents or guardians who would like their adolescent to receive the check-up will be asked to provide their written consent. On the day of the check-up visit, a verbal confirmation of their previous written assent will be requested from the adolescent. In Ghana and Tanzania, where the minimum age for providing consent to medical and health-related research is 18 years, clients of all ages under 18 will provide completed parental consent forms and provide written assent before proceeding through the check-up visit regardless of whether the check-up is in schools or communities. In Zimbabwe, a waiver of parental consent has been given by the Medical Research Council of Zimbabwe (MRC-Zimbabwe) so that participants aged 16 and 17 years who attend the check-ups in the community venues will be allowed to provide written consent for themselves.

For peer review only

For peer review only - http://bmjopen.bmj.com/site/about/guidelines.xhtml

The intervention will be conducted in private and not in the presence of the parent or guardian. Contact details of the study team will be shared with participants in case they have questions at a later stage. All participants will be reminded that participation is entirely voluntary and will be told that they can opt out of the research or services at any time.

Data collection

During the Y-Check intervention and follow up

Data collection during baseline and follow-up visits will include self-completed evaluation questionnaires, self-reported screening tool responses and screening visit consultations, measurements and specimen collection and an exit interview. Data on the implementation process and on adolescent outcomes will be collected in digital and paper-based formats. A user-friendly digital data collection app for the check-ups will be developed and housed on a tablet computer for direct use by the adolescent. Initial sections will include audio-assisted, user-friendly self-completion questions for adolescents to fill out. This will utilize engaging content and processes, tailored to adolescents´ interests. The option of a face-to-face interview will also be available if the adolescent is unable to use the tablet or has low literacy level. Health services registers and school registers will also be reviewed to determine the number of adolescents of the relevant age ranges, and school attendance by the classes involved in Y-Check. To help build the referral process, existing adolescent services will be mapped in the study communities.

Process evaluation

The process evaluation is guided by the UK MRC's Process Evaluation framework to understand intervention implementation (including feasibility and fidelity), mechanisms of impact (including acceptability and uptake), and the influence of context (35). Key implementation outcomes of interest are acceptability, adoption, appropriateness, feasibility, and fidelity. Data on contextual factors and barriers

For peer review only

For peer review only - http://bmjopen.bmj.com/site/about/guidelines.xhtml

and facilitators to programme implementation will be gathered using routinely-collected programme monitoring data. Qualitative data will be collected through 1) observations of the Y-Check intervention and referrals, as well as team meetings; 2) in-depth interviews with eligible adolescents who received, adolescents who were referred, and adolescents who did not receive Y-Check, as well as with school authorities and the Y-Check service providers; and 3) participatory workshops with teachers, adolescents, and parents. Quantitative programme monitoring data will be collected routinely within the Y-Check visit, including through a participant exit interview. Process evaluation data will be analysed iteratively and thematically, through regular analytical discussions and analytical memos to draw out the main themes emerging from the data. Across the pilot and intervention studies, data collection for the process evaluation will include real-time feedback to the implementation team.

Economic evaluation

A costing study will be conducted to estimate the total costs of developing, setting up, and running the Y-Check package, in school and community settings. A combination of top-down and ingredients-based costing approaches will be used to generate cost estimates for the whole package, and for each component/activity. All costs will be estimated from the perspectives of the adolescents, the schools/community and implementing partners/service providers. Financial and economic costs will be calculated for all inputs. These inputs will be identified and measured using process data, staff interviews and observations, document review, and accounting records.

Costs will be inputted and analysed in an Excel-based costing tool. The cost analysis will describe the distribution of costs across different forms of inputs, and will estimate the unit cost per adolescent reached, screened, and treated on the spot or referred; cost per unit of measure for selected process and effect outcomes such as cost per condition detected, cost per condition appropriately treated on-the-spot or with a completed referral within 4 months, cost for a unit improvement in reported quality of life and Disability Adjusted Life Years (DALYs) averted.

For peer review only

For peer review only - http://bmjopen.bmj.com/site/about/guidelines.xhtml

The cost and cost-effectiveness estimates will be compared to other programmes in the region (eg. human papillomavirus vaccination, deworming) and will inform programme replication, scalability, and financial sustainability.

Data protections

Data protection will be strictly observed. After study completion, data will be stored in the LSHTM-curated digital repository ‘Data Compass’ following General Data Protection Regulation (GDPR) guidelines. Data and code registered in LSHTM Data Compass will be made open access following deposit. A Data Safety and Monitoring Board (DSMB) has been constituted to assist in managing adverse events, though we expect these to be very rare since all treatment and care are standard with no novel treatments.

Study outcomes

Outcomes will be ascertained during the check-up screening visit and through collection of referral vouchers from the referral health facilities, and, for outcomes related to health and wellbeing impacts, through data from the 4-month and, in Zimbabwe only, 12-month follow-up visits. Outcomes related to completed referrals will be triangulated against participants′ self-reports at the 4-month and. In Zimbabwe only, 12-month follow-up visits. Review of school and health service registers will be used to see whether attendance has increased during the period when Y-Check is being implemented.

The primary outcome will be the proportion of those screening positive for at least one condition who receive appropriate on-the-spot care or complete appropriate referral for all identified conditions within 4 months. This will be measured using data collected at the initial check-up visit and through recovery of referral vouchers given to participants to allow them to access referral services for free during the 4-months after the Y-Check screening. Completed referral is defined as attending at least the first referral appointment.

For peer review only

For peer review only - http://bmjopen.bmj.com/site/about/guidelines.xhtml

393 Secondary implementation outcomes will include the proportion of those screening positive for each
394 condition who receive appropriate on-the-spot care or complete appropriate referral for that condition
395 within 4 months, the yield of previously untreated conditions, clinical outcomes at 4 months among those
396 who had originally screened positive for each condition, and intervention acceptability, adoption,
397 appropriateness, feasibility, fidelity and cost. Secondary effectiveness outcomes will include knowledge
398 about health services and health behaviours, self-reported agency and self-efficacy to make decisions
399 about their health, self-reported health-related risk and protective behaviours, reported engagement
400 with health services, wellbeing, self-esteem and quality of life, clinical outcomes, and educational
401 outcomes, which will be collected within the Y-Check and follow-up visits. The short-term cost-
402 effectiveness of the intervention will be estimated (calculated by a comparison of the costs of the
403 intervention against the primary and secondary outcomes and including short-term changes in self-
404 reported quality of life). All outcomes for the study are described in Table 3.

405 **Sample size**

406 In each city, the intervention will be implemented for 10-14 year-olds in up to 6 government primary
407 schools (N=500 for young adolescent girls, and N=500 for young adolescent boys), and for 15-19 year-olds
408 in up to 8 secondary schools and up to 3 community venues (N=500 for older adolescent girls, and N=500
409 for older adolescent boys), giving a total sample size of 2,000 adolescents (10-19y).

410

411 The sample size provides specified precision around the primary outcome. For example, for the primary
412 outcome, within each age group and gender, if 150 (30%) of 500 participants screen positive for at least
413 one condition, and 75% of those who screen positive are correctly managed (n=112), the 95% CI for
414 correct management will be +/- 7%. The primary outcome used data from the initial check-up visit and
415 referrals and did not require the 4-month follow-up data.

For peer review only - http://bmjopen.bmj.com/site/about/guidelines.xhtml

Table 3: Study outcomes and means of verification

Outcome	Sources of data
**Primary outcome**	
Proportion of those screening positive for at least one condition who receive appropriate on-the-spot care or complete appropriate referral for all identified conditions within 4 months (i.e. they attend a provider for referral care who has been accredited by the study team and has been shown to be capable of providing appropriate referral care).	• Programme monitoring data including records of attendance for referrals<br>• Screening tool (self-reported symptoms or conditions, measurements and clinical actions)
**Secondary outcomes**	
**Implementation outcomes**	
Proportion of those screening positive for each condition who receive appropriate on-the-spot care or complete appropriate referral for that condition within 4 months.	• Programme monitoring data including records of attendance for referrals<br>• Screening tool (self-reported symptoms or conditions, measurements, and clinical actions)
The yield of previously untreated conditions.	• Programme monitoring data including records of attendance for referrals<br>• Screening tool (self-reported symptoms or conditions, measurements, and clinical actions)
Intervention acceptability (satisfaction): acceptability to adolescents and to other stakeholders (eg. schools, parents, health workers).	• Programme monitoring data including records of attendance for referrals<br>• Screening tool (self-reported symptoms or conditions, measurements, and clinical actions)<br>• Self-completed evaluation questionnaire<br>• Exit interviews<br>• Observations of the Y-Check visits and of selected referrals<br>• Interviews and workshops with adolescents, healthcare providers, community members, teachers, parents and key stakeholders
Intervention adoption (uptake, utilization): Y-Check uptake, referrals completed.	
Intervention appropriateness (perceived fit, perceived relevance, perceived usefulness): perceived value of the intervention to adolescents and to other stakeholders.	
Intervention feasibility (actual fit, practicability): Y-Check visits completed, referrals completed, stakeholder support (including community).	
Intervention fidelity (adherence, integrity, quality): completeness of training for and delivery of intervention components; diagnostic accuracy; youth-friendly health services quality assessment.	• Interviews and workshops with adolescents, healthcare providers, community members, teachers, parents and key stakeholders<br>• Observations of the Y-Check visits and of selected referrals, including youth friendly services<br>• Self-reported screening tool
**Economic outcomes**	
Cost of setting up and running the intervention.	• Y-Check documentation and financial records<br>• Interviews with Y-Check staff and staff of the referral facilities.<br>• Programme monitoring data including records of attendance for referrals
Cost per adolescent with a newly diagnosed condition (overall and by condition).	
Cost per adolescent with a newly diagnosed condition who received appropriate on-the-spot care or who completed an appropriate referral within 4 months (overall and by condition).	

For peer review only - http://bmjopen.bmj.com/site/about/guidelines.xhtml

Short-term (4 months) cost-effectiveness: cost per improvement in health or wellbeing (e.g. cost per case addressed or cured), cost per unit improvement in QALYs and per DALY averted.	• Screening tool (self-reported symptoms or conditions, measurements and clinical actions)
**Client outcomes**	
Knowledge about health services and health behaviours.	• Programme monitoring data including records of attendance for referrals<br>• Screening tool (self-reported symptoms or conditions, measurements and clinical actions)<br>• Self-completed evaluation questionnaire
Intentions to adopt healthy behaviours.	
Agency to make decisions about health and wellbeing.	
Perceived social support for behaviour change.	
Health-related risk and protective behaviours.	
Improvement in previously diagnosed health and wellbeing conditions.	
Engagement with health and other services within the past 4 months.	
Self-esteem.	
Self-perceived wellbeing.	
Quality of life.	
Clinical outcomes.	
Educational outcomes (e.g. school attendance).	• Self-completed evaluation questionnaire<br>• School register review
Client-defined outcomes (to be determined).	• Self-completed evaluation questionnaire<br>• Exit interviews

Statistical analysis

All primary analyses will be conducted separately by study city; Cape Coast, Chitungwiza and Mwanza. Where comparable, secondary analyses will be conducted with the data from all three cities combined.

In our study sites, a contemporaneous comparison group is not required since no routine screening is currently taking place, and as a result, assessments at baseline will serve as the counterfactual for internal comparisons. Similarly, since there is no routine screening and treatment provided to adolescents of the target ages in the study population, a before-after comparison is appropriate since it is plausible to assume that reductions in the prevalence of the chronic conditions between the original Y-Check visit and the follow-up at four months will be due to the interventions provided through Y-Check.

We will follow STROBE guidelines for the reporting of cohort studies. Descriptive analyses will be used to compare the community-level and school-level characteristics of the study communities and schools.

For peer review only - http://bmjopen.bmj.com/site/about/guidelines.xhtml

429 Quantitative programmatic data, including screening test results, services delivered, and referrals made and completed, will be reported by age, sex, and city. The primary outcome is a single proportion which will be presented with a 95% confidence interval for each of the 4 target groups: 10–14-year-old males, 10-14 year-old females, 15-19 year-old males, 15-19 year-old females.

433 Secondary outcomes which are measured at a single time point will be presented in a similar way to the primary outcome. For outcomes which are measured at two or more time points, a before-after analysis will be conducted comparing differences in measures between the time points. The unit of analysis will be the individual. For clinical outcomes which are measured at two or more time-points, the initial check-up visit (baseline) will give the prevalence of untreated conditions which will represent the counterfactual. The prevalence of conditions at the 4-month follow-up visit will be formally compared to this counterfactual to estimate the short-term effects of the intervention in improving these clinical outcomes. For analysis of outcomes measured at two timepoints we will use mixed effects logistic regression (binary outcomes) or linear regression (continuous outcomes) adjusting for individual-level clustering as a random effect and school/community as a fixed effect. Health service and client determinants of correct management of conditions at 4 months will be analyzed using multivariable regression.

444 Ethics and Dissemination

445 Ethics clearance has been received from WHO (WHO/ERC.0003778) and from all country national ethics bodies. Protocol modifications will be shared with the WHO Ethics Review Committee and relevant national ethics boards. Results will be published in at least 3 country-specific peer reviewed journal publications and one multi-country publication. There will also be videos, briefs, webinars and meetings to disseminate results. All data will be placed into an open access repository after deidentification and anonymisation to ensure confidentiality and participant privacy.

For peer review only

Discussion

Over the last decade, adolescent wellbeing has become a global priority (5). School health is also a growing area of policy interest (36). WHO guidelines on school health services note that along with health promotion, health education, preventive interventions (such as immunizations and mass drug administration), clinical assessment and health services management, health screenings within school learners are one of the key pillars in the delivery of comprehensive school health services (16). Screening programs such as Y-Check provide a unique opportunity to detect easily treatable, high-burden health conditions, refer those requiring medical attention, treatment and care, as well as to advise and encourage adolescents to engage in healthy behaviours.

In a 2015 review, school health services were found to exist in at least 102 countries though their content varied considerably across 16 areas including vaccinations, sexual and reproductive health education, vision screening, nutrition screening, and nutrition health education (37). If all types of screening were combined, they were the second most commonly reported intervention in school health services, second only to immunization. A later systematic review found evidence of routine health check-ups of school age children having been reported in 86 countries worldwide (17). Despite their widespread existence, little quality evidence exists on how to promote good health for adolescents in educational settings (37), and even less for multi-component school health services (38) especially in low- and middle-income countries (39).

Good practices in conducting adolescent health or wellbeing screenings are rarely reported. In 2023, WHO will release new guidance on well-child and well-adolescent visits, which will recommend expanding routine screening tests to also integrate other wellbeing dimensions through a broader evaluation of social risks, emotional state, and individual and family resources delivered with context-specific

For peer review only - http://bmjopen.bmj.com/site/about/guidelines.xhtml

recommendations at key moments during the first two decades of life. The successful implementation of such guidance requires robust measurement of the effectiveness of preventive interventions in adolescence (40).

Evaluation of the Y-Check intervention will incorporate implementation science and effectiveness research. Such hybrid designs have important advantages over conducting separate studies. These include the potential for quicker translation of intervention research findings into programmes, the development and selection of more effective implementation strategies, and more useful information for decision makers (41).

The process evaluation findings will provide guidance for the next stage of the programme and for potential future sustainable and scalable implementation by local health authorities should it prove successful. Data on the short-term changes in clinical and behavioural outcomes will be used as inputs to model both short-term and long-term health and social impacts and as inputs to sample size and power calculations for a third phase of the Y-Check research programme, which plans to undertake a rigorous population level evaluation of the impact of routine check-ups on adolescent health and wellbeing.

Through WHO's advice to member states, findings from the Y-Check study have the potential to shape the delivery of adolescent health check-ups globally including identifying the optimal number, content and delivery for these services. Y-Check will advance the field by providing some of the first rigorous information on the effects of a health screening programme in three African cities, assessing implementation, effectiveness, cost and cost-effectiveness outcomes.

Acknowledgements: The authors thank our Programme Advisors: Prof Simon Gregson, Dr. Sachin Shinde, Prof Fred Binka, Dr. Suzanne Petroni, Prof. Audrey Pettifor, Prof Shane Norris, Prof. James Hargreaves, Ms. Patience Manhibi, Mr Aveneni Mangome. DSMB members are Prof. David Mabey, Dr Andrew Abassa, Prof Fred Binka and Dr Nothando Ngwenya. The authors thank adolescent participants in three countries.

For peer review only - http://bmjopen.bmj.com/site/about/guidelines.xhtml

The support of participating Ministry officials in Ghana, Tanzania and Zimbabwe is gratefully acknowledged.

Authors' contributions: PB, AMD, DAR conceived and drafted the paper. All authors contributed to writing. BW, SK, AMD, FN, FG and MKN provided country specific inputs. GG contributed to the economic evaluation section. HAW contributed to the study outcomes and statistical analysis sections. VB and RAF contributed to the sections on consent, introduction and discussion.

Banati P, PhD MPH[1*], Ross DA, MA, BMBCh, MSc, PhD[2] Weobong B, MSc, PhD[3], Kapiga S, MD, MPH, ScD[4,5], Weiss HA PhD MSc[6], Baltag V MD PhD[1], Nzvere F[5,7], Glozah F, MPhil, PhD[3], Nsanya MK, MD, MSc[4], Greco G, PhD MSc[5], Ferrand RA, PhD MBBS[5,7], and Doyle AM, PhD[6]

Competing interests: PB and VB are WHO staff members. The opinions expressed are theirs and do not necessarily represent the policies and positions of the World Health Organization. The other authors declared no competing interests.

Funding statement: This work was supported by the Botnar Foundation grant number (RG21-001) and UKRI (MR/T043156/1)

For peer review only

References

1. Bundy DAP, de Silva N, Horton S, Patton GC, Schultz L, Jamison DT. Investment in child and adolescent health and development: key messages from Disease Control Priorities, 3rd Edition. Lancet. 2018;391(10121):687-99.
2. World Bank. World Development Report 2019: The Changing Nature of Work. Washington, DC: World Bank; 2019.
3. Tomlinson M, Ross DA, Bahl R, et al. What will it take for children and adolescents to thrive? The Global Strategy for Women's, Children's, and Adolescents' Health. Lancet Child Adolesc Health. 2019;3(4):208-9.
4. WHO. Global accelerated action for the health of adolescents (AA-HA!): guidance to support country implementation, 2017.
5. WHO. Accelerated Action for the Health of Adolescents (AA-HA! 2.0) Forthcoming. Geneva: World Health Organization; 2023.
6. Kuper H, Monteath-van Dok A, Wing K, et al. The impact of disability on the lives of children; cross-sectional data including 8,900 children with disabilities and 898,834 children without disabilities across 30 countries. PLoS One. 2014;9(9):e107300.
7. WHO. Health for the World's Adolescents: A second chance in the second decade. Geneva: World Health Organization; 2014.
8. Kruk ME, et al. Improving health and social systems for all children in LMICs: structural innovations to deliver high-quality services. Lancet. 2022;399(10337):1830-1844.

For peer review only - http://bmjopen.bmj.com/site/about/guidelines.xhtml

9. World Health Organization (WHO). Making health services adolescent friendly: developing national quality standards for adolescent friendly health services, 2012.
10. Patton GC, Sawyer SM, Santelli JS, et al. Our future: a Lancet commission on adolescent health and wellbeing. Lancet. 2016;387(10036):2423-2478.
11. Sanci L. Clinical preventive services for adolescents: facing the challenge of proving "an ounce of prevention is worth a pound of cure". J Adolesc Health. 2011;49(5):450-2.
12. Harris SK, Aalsma MC, Weitzman ER, Garcia-Huidobro D, Wong C, Hadland SE, Santelli J, Park MJ, Ozer EM. Research on clinical preventive services for adolescents and young adults: where are we and where do we need to go?. Journal of Adolescent Health. 2017 Mar 1;60(3):249-60.
13. Nagata JM, Hathi S, Ferguson BJ, Hindin MJ, Yoshida S, Ross DA. Research priorities for adolescent health in low- and middle-income countries: A mixed-methods synthesis of two separate exercises. J Glob Health. 2018;8(1):doi:10.7189/jogh.08.010501.
14. Bright Futures, American Academy of Pediatrics. Bright Futures. Guidelines for health supervision of infants, children, and adolescents. 2016;4.
15. Royal Australasian College of General Practitioners. Guidelines for Preventive Activities in General Practice, Volume Spec No. 8th ed. East Melbourne: Royal Australasian College of General Practitioners; 2012.
16. WHO. WHO Guideline on School Health Services. 2021.
17. Baltag V, Moran D. Are Routine Health Examinations Fulfilling their Promise for Prevention? Health Behav Policy Rev. 2018;5(6):22.
18. Hoffmann TC, Glasziou PP, Boutron I, et al. Better reporting of interventions: template for intervention description and replication (TIDieR) checklist and guide. BMJ. 2014;348.
19. Chingono RM, Mackworth-Young CRS, Ross DA, et al. Designing routine health check-ups for adolescents in Zimbabwe. J Adolesc Health. 2021;69:940-7.
20. Weobong B, Glozah FN, Abdulai HBT, Koka E, Addae N, Alor S, Kohl K, Banati P, Adongo PB, Ross DA. Reaching adolescents with health services: systematic development of an adolescent health check-ups programme in Ghana (Y-Check, Ghana). (in preparation).
21. Sedekia Y, Mshana G, Nsanya MK, Kohl K, Wambura M, Grosskurth H, Ross DA, Kapiga S. Routine health check-ups for adolescents in Mwanza City, Tanzania: stakeholders' recommendations on its content, venue, and mode of delivery. BMC public health. 2023 May 30;23(1):1015.
22. WHO. Report of Y check Phase 1 to Botnar Foundation, 2020.
23. Green L, Kreuter M. The precede–proceed model. Health promotion planning: an educational approach. 3rd ed. Mountain View (CA): Mayfield Publishing Company; 1999. p. 32-43.
24. McLeroy KR, Bibeau D, Steckler A, Glanz K. An ecological perspective on health promotion programs. Health Educ Q. 1988;15(4):351-377.
25. UNESCO. Net enrollment statistics available at htttp://uis.unesco.org. Downloaded March 18 2023.
26. National Bureau of Statistics. Mwanza Region - Basic Demograhic and Socio-Economic Profile (2012 Census) - National Bureau of Statistics (NBS).; 2016.
27. Ilemela Municipal Council. Ilemela Municipal Council Strategic Plan (2016/2021).; 2017. doi:10.5089/9781513547442.002
28. Zimbabwe National Statistics Agency (ZIMSTAT). Education Statistics Report 2018-2020. Published November 2021. Available from: https://www.zimstat.co.zw/wp-content/uploads/publications/Social/Education/Education_Report_2020.pdf.

For peer review only - http://bmjopen.bmj.com/site/about/guidelines.xhtml

29. Zimbabwe Ministry of Primary and Secondary Education (MoPSE). 2021 Primary and Secondary Education Statistics Report. Published May 2022. Available from: http://mopse.co.zw/sites/default/files/public/downloads/EMIS%202021%20Annual%20Education%20Statistics%20Report.pdf.
30. Government of Zimbabwe. Zimbabwe School Health Policy. Harare, Zimbabwe; 2018.
31. Ghana Health Service. Adolescent health service policy and strategy, 2016-2020. Accra, Ghana Health Service. 2016.
32. Government of Tanzania. Health Sector Strategic Plan July 2021 – June 2026 (HSSP V): Leaving No One Behind. Ministry of Health, Community Development, Gender, Elderly and Children, Dar es Salaam; 2021.
33. WHO. Global standards for quality health-care services for adolescents: a guide to implement a standards-driven approach to improve the quality of health care services for adolescents. 2015 Available from: https://apps.who.int/iris/handle/10665/183935.
34. WHO. Assessing and supporting adolescents' capacity for autonomous decision-making in health care settings: a tool for health-care providers. Geneva: World Health Organization; 2021.
35. Moore GF, Audrey S, Barker M, et al. Process evaluation of complex interventions: Medical Research Council guidance. BMJ. 2015;350:h1258.
36. WHO and UNESCO. Making every school a health-promoting school: global standards and indicators for health-promoting schools and systems. Geneva: World Health Organization and UNESCO; 2021.
37. Baltag V, Pachyna A, Hall J. Global overview of school health services: data from 102 countries. Health Behav Policy Rev. 2015;2(4):268-83.
38. Levinson J, Kohl K, Baltag V, Ross DA. Investigating the effectiveness of school health services delivered by a health provider: a systematic review of systematic reviews. PLoS One. 2019;14(6):e0212603.
39. Montgomery P, Knerr W, Ross DA, Patterson J. The effectiveness and acceptability of comprehensive and multicomponent school health services: A systematic review. J Adolesc Health. 2022;70:192-207.
40. Banati P, Banerjee A, Daelmans B, Baltag V. The need for new models to measure the impact of prevention. J Adolesc Health. 2023;72(1):9-11.
41. Curran GM, Bauer M, Mittman B, Pyne JM, Stetler C. Effectiveness-implementation hybrid designs: combining elements of clinical effectiveness and implementation research to enhance public health impact. Med Care. 2012;50(3):217-26.

Figure 1: Theory of Change for Y-Check, an adolescent health and wellbeing check-up

Figure 2: The Y-Check Intervention package[1]

For peer review only - http://bmjopen.bmj.com/site/about/guidelines.xhtml

Figure 1: Theory of Change for Y-Check, an adolescent health and wellbeing check-up

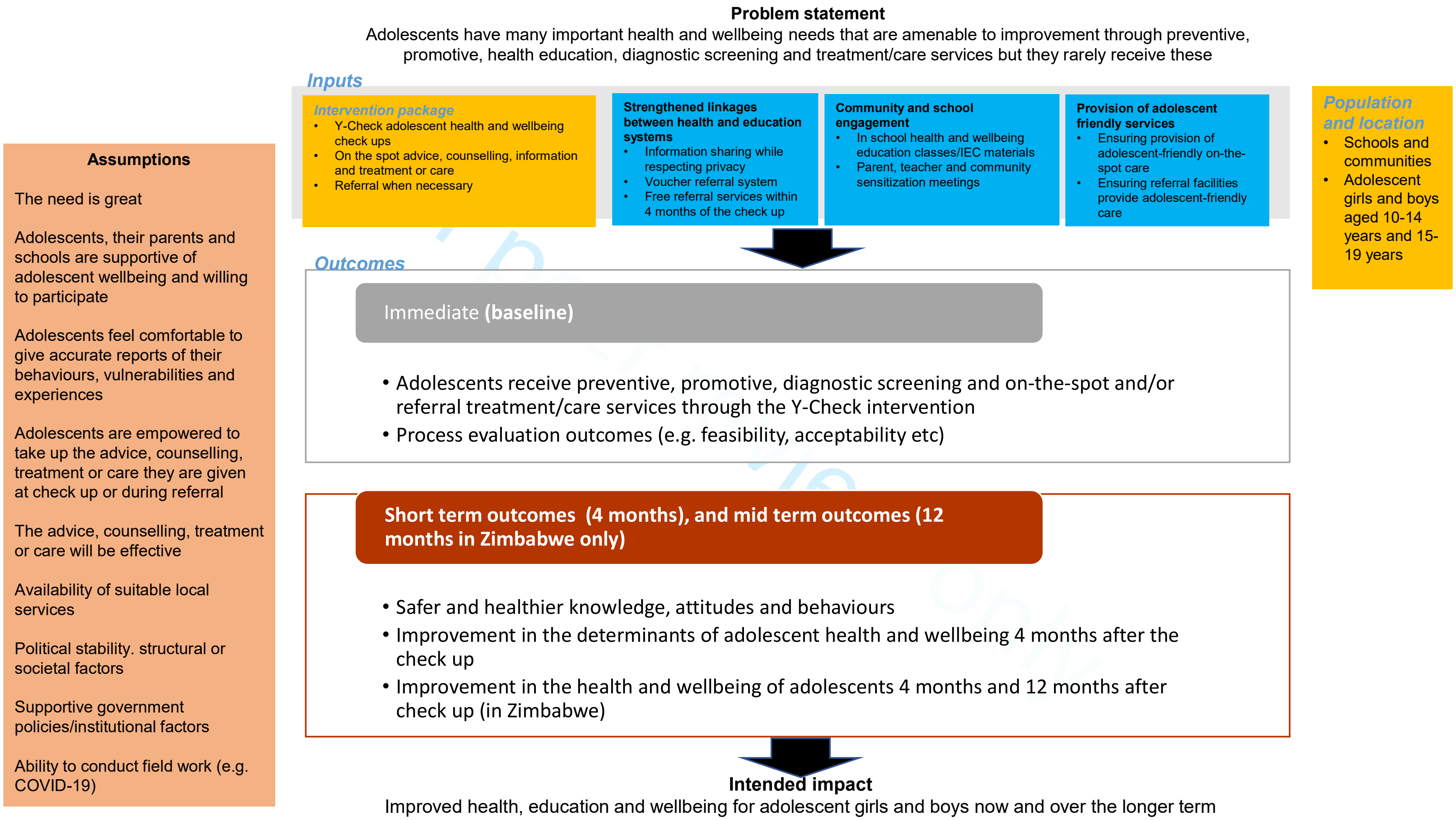

Figure 2: The Y-Check Intervention package[1]

Y-Check screening, on-the-spot care and referral services

Station 1
Registration and private pre-consultation screening questionnaire including tobacco and substance use, physical activity, diet, psychosocial and mental health, sexual activity (only in community settings) and other areas

Station 2
Physical examination including blood pressure, anthropometry, oral, vision and hearing exams and physical impairment

Station 3
Laboratory/ point of care tests including haemoglobin, HIVs and STIs (only in community settings), malaria, sickle cell and schistosomiasis (high prevalence cities only)

Station 4
Consultation review and intervention with clinician/nurse including on-the spot care for iron folic acid treatment, PrEP or STI treatment or contraception (sexual activity in community settings only) and further referral to services if indicated

Station 5
Health commodities Participants receive tooth cleaning kit, counselling, menstrual health kit (only girls), health promotion literature

[1] The intervention package may vary according to setting

For peer review only

Table 1: Template for Intervention Description and Replication (TIDieR) checklist describing the Y-Check intervention

Item	Item
**Brief name**	
1	Evaluating the effectiveness of adolescent health check-ups (Y-Check)
**Why?**	
2	Identifying adolescents with poor health, health-compromising behaviours or undiagnosed disability is important for their health and wellbeing, and also for communities and nations<br>Most adolescents only come into contact with health services when they are ill, and services are not always appropriate for their needs<br>Routine health and wellbeing check-up visits for adolescents that screen for multiple preventable and/or treatable conditions and risk behaviours could provide an entry point into services and be highly cost-effective
**What?**	
3	The intervention includes a comprehensive health check-up for priority conditions customized to national and local contexts.<br>Where indicated, Y-Check will provide on-the-spot care and cover all clinical costs associated with referrals to further care provided by the public health system or non-governmental organizations (NGOs).<br>During the check-up, adolescents will receive health promotion information and limited supplies of key health commodities.<br>Clinical costs of services are covered by the study if accessed within 4 months of the check-up.
4	Adolescent-friendly services will be provided, as defined by WHO (2018). Nationally-approved protocols will be applied. Adolescent privacy and confidentiality will be protected.
**Who provided?**	
5	Y-Check teams will be staffed with health professionals trained to provide quality adolescent-friendly health services in line with nationally-approved protocols. Y-Check teams will also be trained in the use of the digital application which will be used for data collection. Public and private not-for-profit care facilities providing referrals will meet national accreditation guidelines.
**How?**	
6	The Y-Check service will take place over a 60-90 minute period face-to-face. Any referrals will only be subsidized by the study if they take place within 4 months.
**Where?**	
7	The Y-Check service will be provided in schools and community venues, in outdoor tents where required.<br>Referrals will be to public or private not-for-profit providers as close as possible to the adolescent´s home. Providers will be vetted by the study team as being able to provide the necessary referral services to national and WHO-recommended standards.
**When and How Much?**	
8	Within the current phase of the study, each adolescent will receive Y-Check once. Within a routine programme the intention would be that the intervention will be delivered twice during adolescence, once when the adolescent is 10-14 years old, and a second time when they are 15-19 years old.
**Tailoring**	
9	The content of the intervention is tailored to local context. The exact set of conditions that will be assessed as part of Y-Check will be adapted based on burden of disease, and availability of local tests and referral services.
**Modifications**	

Item	Item
10	Any modifications will be reported in the article reporting the results of the study.
**How well?**	
11	Intervention fidelity (adherence, integrity, quality) will be evaluated through a process evaluation including youth-friendly health services quality.
12	Intervention fidelity will be reported in the article reporting the results of the study.

For peer review only

STANDARD PROTOCOL ITEMS: RECOMMENDATIONS FOR INTERVENTIONAL TRIALS

SPIRIT 2013 Checklist: Recommended items to address in a clinical trial protocol and related documents*

Section/item	Item No	Description
**Administrative information**		
Title	1	A hybrid evaluation of implementation and short-term cost-effectiveness of Y-Check, an adolescent health and wellbeing check-up programme in three African cities
Trial registration	2a	Registration Protocol ID WHO/ERC.0003778 28/08/2023
	2b	ClinicalTrials.gov Identifier: NCT06090006
Protocol version	3	January 10 2023, Version 4
Funding	4	World Health Organization, Botnar Foundation, UKRI, University of Ghana, Biomedical Research Training Institute Zimbabwe, Mwanza Intervention Trials Unit, Tanzania, London School of Hygiene and Tropical Medicine

For peer review only

Roles and responsibilities 5a

Name of collaborator	Institute and contact details
Prerna Banati (Chief investigator)	Research Scientist<br>World Health Organization<br>Ave Appia 20,<br>1211 Geneva, Switzerland
Valentina Baltag (co-PI)	Team lead<br>AYH, MCA<br>World Health Organization<br>Ave Appia 20,<br>1211 Geneva, Switzerland
Aoife Doyle (PI Zimbabwe)	Associate Professor<br>Biomedical Research and Training Institute, Harare, Zimbabwe<br>and<br>MRC International Statistics & Epidemiology Group<br>Department of Infectious Disease Epidemiology and International Health<br>London School of Hygiene & Tropical Medicine<br>Keppel Street
Rashida Ferrand (co-PI)	Professor<br>Biomedical Research and Training Institute, Harare, Zimbabwe<br>and<br>Department of Clinical Research<br>London School of Hygiene & Tropical Medicine<br>Keppel Street
Saidi Kapiga (PI Tanzania)	Professor<br>Mwanza Intervention Trials Unit,<br>National Institute for Medical Research,<br>Isamilo Road, Mwanza, Tanzania
Helen Weiss (co-PI)	Professor<br>MRC International Statistics & Epidemiology Group<br>Department of Infectious Disease Epidemiology and International Health<br>London School of Hygiene & Tropical Medicine<br>Keppel Street
Benedict Weobong (PI Ghana)	Lecturer<br>School of Public Health, University of Ghana
David Ross (Co-I)	Honorary Professor<br>Institute of Life course studies<br>Stellenbosch University<br>Stellenbosch South Africa
Mussa Nsanya (co-I)	*Research Scientist*,<br>Mwanza Intervention Trials Unit,<br>National Institute for Medical Research,<br>Isamilo Road, Mwanza, Tanzania
Gerry Mshana (co-I)	Principal Research Scientist,<br>Mwanza Intervention Trials Unit, National Institute for Medical Research, Isamilo Road, Mwanza, Tanzania
Yovitha Sedekia (co-I)	Post-doctoral Fellow,<br>Mwanza Intervention Trials Unit, National Institute for Medical Research, Isamilo Road, Mwanza, Tanzania
Chido Dziva Chikwari (co-I)	Biomedical Research and Training Institute, Harare, Zimbabwe<br>and<br>Department of Infectious Disease Epidemiology and International Health<br>London School of Hygiene & Tropical Medicine<br>Keppel Street
Salome Manyau (co-I)	Biomedical Research and Training Institute, Harare, Zimbabwe
Farirai Nzvere (co-I)	Research Manager<br>Biomedical Research and Training Institute, Harare, Zimbabwe
Franklin Glozah (co-I)	Research Scientist<br>School of Public Health, University of Ghana
Philip Adongo (co-I)	Professor<br>School of Public Health, University of Ghana
Eric Koka (co-I)	Research Scientist<br>University of Cape Coast<br>Ghana
Evans Agbeno (co-I)	Research Scientist<br>University of Cape Coast<br>Ghana
Kenneth Addo (Program manager)	Research manager<br>School of Public Health, University of Ghana
Hannah Taylor-Abdulai (co-I)	University of Cape Coast<br>Ghana
Giulia Greco (co-I)	*Assistant Professor in Economics*<br>Department of Global Health and Development<br>London School of Hygiene & Tropical Medicine<br>Keppel Street<br>London WC1E 7HT, UK
Constance Mackworth-Young (co-I)	*Assistant Professor*<br>Biomedical Research and Training Institute, Harare, Zimbabwe<br>and<br>Department of Global Health and Development<br>Faculty of Public Health and Policy<br>London School of Hygiene & Tropical Medicine<br>Keppel Street<br>London WC1E 7HT, UK
Victoria Simms (co-I)	Biomedical Research and Training Institute, Harare, Zimbabwe<br>and<br>MRC International Statistics & Epidemiology Group<br>Department of Infectious Disease Epidemiology and International Health<br>London School of Hygiene & Tropical Medicine<br>Keppel Street<br>London WC1E 7HT, UK

For peer review only - http://bmjopen.bmj.com/site/about/guidelines.xhtml

5b World Health Organization (Study sponsor), Ave Appia 20, 1211 Geneva Switzerland

5c Study sponsor provides country coordination, oversight and quality control of study design, data collection, management, analysis, and interpretation; writing of the report

5d **Coordinating center** (WHO) provides country coordination, oversight and quality control of study design, data collection, management, analysis, and interpretation, writing of the report. **Implementing centers** (BRTI, MITU, UGSPH) are responsible for identification, recruitment, data collection and completion of national ethical protocols, along with follow up of study participants and adherence to study protocol. **Programme Advisory Committee (independent)** provides research advise and review of technical and scientific aspects to the research, review and comment on papers; provide recommendations for uptake of results. **Data Safety Monitoring Board (DSMB) (independent)** monitors evidence for harm, assess the impact and relevance of external evidence, asesss whether study follow up should be stopped earlier, assess data quality, monitor recruitment figures and sample size, consider ethical implications, advise on modifications as needed.

For peer review only

Background and rationale	6a	To develop and implement a potentially sustainable adolescent health check-up programme in three African cities (Cape Coast, Ghana; Mwanza, Tanzania; Chitungwiza, Zimbabwe) and evaluate the acceptability, feasibility, short-term effects, and cost-effectiveness of the programme to improve adolescents´ health and well-being. Systematic reviews have identified individual interventions that are effective at improving various aspects of adolescent health and/or well-being. However, most adolescents only come in contact with health services when they are ill, and services are not always appropriate for their needs. This represents a missed opportunity for early detection of health problems and for health promotion, and for the development of beneficial health-seeking behaviours. Early and sustained engagement with health and social services could reap a triple dividend for human development by improving the health and well-being of adolescents, their health and well-being in adulthood and the health and well-being of their future offspring.<br><br>Routine health and well-being check-up visits for adolescents which screen for multiple conditions and risk behaviours, could provide an entry point into services and be highly cost-effective but there is little empirical evidence for their feasibility, acceptability and effects. Many high-income countries have national recommendations related to adolescent health check-ups (largely based on expert opinion). In low- and middle-income settings, preventive health services for adolescents are largely provided in schools, are usually limited to deworming and vaccination campaigns, and do not address other important conditions and risk factors such as nutrition, mental health, or disability. Obtaining evidence on check-ups is a high World Health Organization (WHO) priority for adolescent health research so that they can advise governments on whether or not to start, or to strengthen existing health and well-being check-ups during adolescence and, if so, to develop recommendations for the content and method of delivery of these preventive and promotive contacts.
	6b	In our study sites, a contemporaneous comparison group is not required since no routine screening is currently taking place, and as a result, assessments at baseline will serve as the counterfactual for internal comparisons. Similarly, since there is no routine screening and treatment provided to adolescents of the target ages in the study population, a before-after comparison is appropriate since it is plausible to assume that reductions in the prevalence of the chronic conditions between the original Y-Check visit and the follow-up at four months will be due to the interventions provided through Y-Check.

For peer review only - http://bmjopen.bmj.com/site/about/guidelines.xhtml

Objectives	7	(1) To develop and pilot test a check-up programme for adolescents that screens for important preventable and treatable health conditions using accurate and acceptable screening tests and provides locally accessible effective interventions.<br><br>(2) Through a prospective intervention study in selected schools and communities to: Estimate short-term impacts on adolescent health and wellbeing outcomes: clinical outcomes, health-related knowledge and behaviours, intentions, agency, and perceived social support for behaviour change; engagement with health services, Understand, through process evaluation, the feasibility and fidelity of implementation, the acceptability and uptake, and the influence of context. Estimate the cost-effectiveness of the programme in reducing overall disease burden and improving adolescent wellbeing.<br><br>(3) Obtain information on key parameters needed for the planning of an evaluation study: prevalence of health conditions and behaviours, acceptability of referral, feasibility of following-up programme participants and delivering quality follow-up care, initial estimates of the impact of the programme on longer-term health, educational and wellbeing outcomes based on the short-term implementation and effectiveness outcomes observed in this phase of the research programme, and factors related to the optimal implementation of the Y-Check intervention.<br><br>(4) To refine the programme and its theory of change, and finalise optimal methods for the measurement of the impact of the programme in future studies.
Trial design	8	In this study we propose to conduct implementation science studies to rigorously evaluate the check-ups in real life. We will not conduct a randomized controlled trial (RCT) because the logical next step is to check that it is really feasible and acceptable to deliver the intervention in real life before embarking on a large-scale RCT. As a result, no control group is proposed in this protocol. However, we will include a pilot implementation research study of the intervention that could be tested in the future that will establish the frequency of key health and behavioural outcomes and their short-term impact after 4 months on the health and well-being of the adolescents receiving the intervention through a before-after comparison. We will also use the opportunity to design and pilot test the creation of a Digital Adolescent Health and Well-being Club by recruiting adolescents into the club during the Y-Check screenings.

Methods: Participants, interventions, and outcomes

For peer review only

Study setting	9	**Ghana**: Cape Coast Metropolis has a total population of 169,894 with three-quarters of the households residing in urban areas. The population in the age group 11-24 years has a literacy rate of about 97%. In 2016 in Cape Coast, 11,233 (68.8%) of 12-14 year-olds were enrolled in junior high schools while 8,407 (91.6%) of 15-17 year-olds were enrolled in senior high schools. Primary and secondary net enrolment rates in 2018 were 84% and 58%, respectively. There are 36 health facilities (26 public and 10 private), including a regional hospital that serves as a secondary referral facility. In the formative phase of Y-Check four communities (Abura, Efutu, Akon, and Kwaprow) within the Cape Coast metropolitan area were involved. Abura and Kwaprow are relatively affluent communities with trading being the main source of livelihood. Akon and Effutu are relatively poorer communities where fishing and farming dominate economic activity, respectively. A total of 172 participants were involved in the study: 16 Key Informants (10 male); 41 younger adolescents (in one school, 11 students were selected (one additional girl in 8th year participated) (mean age: 12 years; 21 female) and their parents; and 37 older adolescents (mean age: 16 years; 22 female) and their parents. For this phase of Y-Check, the study will be conducted in 8 schools within the catchment area of health facilities in all the four communities in the first phase. **Tanzania**: Mwanza is the second largest city in Tanzania after the commercial city of Dar es Salaam. It is located on the southern shores of Lake Victoria in North-western (NW) Tanzania. It has a population of over 900,000 with an annual growth rate of 3%. The primary traditional economic activities include fishing and industrial fish processing for export markets, subsistence agriculture and large and small-scale mining of gold and diamond. Adolescents aged 10 to 19 years make up 24.2% of the total population. As of 2020, gross primary and secondary school enrolment stood at 96.9% and 31.4% respectively. Available public health services include 26 dispensaries, 5 health centres, 2 district hospitals, 1 regional hospital and 1 tertiary/teaching hospital. This study will be conducted in 4 – 6 purposive selected communities and in up to 8 primary schools and 8 secondary schools within the catchment area of health facilities serving the selected communities in the two districts (Nyamagana and Ilemela) within Mwanza city. **Zimbabwe**: Chitungwiza is the third largest city in Zimbabwe, located approximately 25km south of the capital city, Harare, and has a population of about 456 000. The houses are mostly high-density, single story, detached units with small yards that are generally used for growing vegetables. Most of the people work in Harare, as there is little industry in Chitungwiza. There is one tertiary hospital, 4 public primary healthcare facilities, 20 private medical facilities, and 34 government primary schools (all mixed sex). Four communities and four schools were chosen by Chitungwiza stakeholders to take part in the Y-Check Phase 1 formative work in 2019/20 (High schools: Seke High 6, Zengeza High 1; Primary schools: Dungwiza Primary, Chinembiri Primary). Communities and schools were selected to represent the diversity of wards in the town and took into account economic disparities. The selection of the schools and communities for this study will be conducted in collaboration with stakeholders including MoPSE, MoHCC, and the study Youth Advisory Group (YAG) taking into consideration previous participation in the formative work and the location of other ongoing projects. We will aim to work in four distinct communities which are representative of the urban, peri-urban and rural populations of Chitungwiza. Potentially eligible schools must meet the following criteria: Student population of at least 200 learners in Grade 6 or at least 75 learners in Form 5, and located in or close to one of the selected study communities.

For peer review only

Eligibility criteria	10	To be included in the study, adolescents aged 10-19 must fall into one of the following categories:<br><br>1) Be attending selected classes of Year 5 of primary school in Mwanza (median age 11 years); Grade 5/6 of primary school in Chitungwiza (median age 11 years); or Year 1 of Junior Secondary School in Cape Coast (median age 12 years) OR<br><br>2) Be attending selected classes in Year 3 of Secondary School in Mwanza (median age 17 years), Form 3/4 in Chitungwiza (median age 17 years), or Year 2 of Senior Secondary School in Cape Coast (median age 16 years) OR<br><br>3) Be resident in a selected community during the time of the Y-Check intervention, and be aged 16-19 years<br><br>AND<br><br>4) Have a completed and signed Informed Consent form, or a signed Informed Assent Form and signed Parental/Guardian Informed Consent Form if the adolescent is seen in the community and is below the national age of consent or is seen in a school, irrespective of their age.
Interventions	11a	Y-Check is a novel intervention delivering an adolescent friendly health and wellbeing check-up and where indicated will provide on-the-spot care and/or referral for common conditions on two occasions in adolescence (in young adolescents (10-14 year-olds) – soon after the onset of puberty - and in older adolescents (15-19 year-olds) – when many adolescents become, or are soon to become, sexually active). The intervention will be customised to national and local context. Adolescents will only be screened for conditions that have an accurate, low-cost, acceptable screening test and a locally accessible, effective intervention. The conditions selected for screening will be chosen to reflect the local epidemiological contexts (e.g. screening for malaria will only take place in malaria endemic areas). It will also provide health promotion information and materials to support positive behaviours and healthy lifestyles during adolescence and beyond. Respecting specific requests from the Ministries of Education in all three cities, the study will only include sexual and reproductive health screening and services at the community sites (which only include older adolescents). Locally accessible services will be identified and assessed in terms of their ability to provide the services recommended by local and WHO guidelines, willingness to accept referred adolescents, and the fees charged to the project will be negotiated by the research team for services provided to referred adolescents (where adequate services are not covered by national health insurance schemes, free NGO services or free public health care).

	11b	The consent form identifies the process for withdrawing from the study. If a participant withdraws from the research study and does not consent to further use of their data, we will remove their records from future use to the fullest extent this is possible. As all tests and procedures follow WHO or accredited national guidelines, these will be used as the basis for adaptation, discontinuing or modifying the diagnosis, treatment or care protocols for specific conditions. If urgent care is required during the course of the Y-Check up, they will be supported to attend a local health facility.
	11c	As for any health care, the tests and treatment provided can have side-effects that can be serious or minor. The tests could cause anxiety. The blood test could cause discomfort or a small bruise, as with any other blood test. While the possibility of this happening is low, the informed consent and assent forms will specify these risks clearly to make sure that participants are aware of the possibility. In the unlikely case of an adverse event, the team will be trained to provide care and support, as well as notify the relevant school authorities (for those seen in schools). If urgent care is required, they will be supported to attend a local health facility. Risks will be minimized by explaining the procedures in detail to adolescents during the school sessions, as well as during the process of obtaining informed consent in schools and community venues. Staff will be trained to detect adverse events and a protocol will be in place to ensure action in the rare case that such an event occurs. Table 3 defines the reporting schedule of adverse events. The use of a digital questionnaire is convenient and has the advantage of providing anonymity; however, adolescents may have fears over unauthorised access and trust. There is also a risk to participants of a breach of confidentiality and possible rejection and discrimination by friends and family if they test positive for any of these conditions. The study team will put in place procedures to minimize these risks. The Y-Check team will be trained in good clinical practice, data protection and confidentiality, and counselling for participants testing positive for any previously mentioned conditions.
	11d	There are no prohibitions during the trial period.
Outcomes	12	The primary outcome will be the proportion of those screening positive for at least one condition who receive appropriate on-the-spot care or complete appropriate referral for all identified conditions within 4 months. This will be measured using data collected at the initial check-up visit and through recovery of referral vouchers given to participants to allow them to access referral services for free during the 4-months after the Y-Check screening. Completed referral is defined as attending at least the first referral appointment.<br><br>Secondary implementation outcomes will include the proportion of those screening positive for each condition who receive appropriate on-the-spot care or complete appropriate referral for that condition within 4 months, the yield of previously untreated conditions, clinical outcomes at 4 months among those who had originally screened positive for each condition, and intervention acceptability, adoption, appropriateness, feasibility, fidelity and cost. Secondary effectiveness outcomes will include knowledge about health services and health behaviours, self-reported agency and self-efficacy to make decisions about their health, self-reported health-related risk and protective behaviours, reported engagement with health services, wellbeing, self-esteem and quality of life, clinical outcomes, and educational outcomes, which will be collected within the Y-Check and follow-up visits. The short-term cost-effectiveness of the intervention will be estimated (calculated by a comparison of the costs of the intervention against the primary and secondary outcomes and including short-term changes in self-reported quality of life).

For peer review only - http://bmjopen.bmj.com/site/about/guidelines.xhtml

Participant timeline 13

Activity	2021		2022		2023				2024				2025	
	Q 3	Q 4	Q 3	Q 4	Q 1	Q 2	Q 3	Q 4	Q 1	Q 2	Q 3	Q 4	Q 1	Q 2
Inception	*	*												
Intervention development and pilot testing			*	*	*									
Implementation of Y-Check					*	*								
Research cohort recruitment					*	*								
Follow-up at 4 months							*							
Process and economic evaluation									*	*				
Analysis, reporting and dissemination										*	*	*	*	*

*Study timeline for Zimbabwe.

Sample size 14

The package will be delivered to 2000 adolescents (500 per gender in each age group), however, the primary outcome is only measured among those who screen positive for at least one condition. Within one age group and gender, if 150 (30%) of 500 participants screen positive for at least one condition, and 75% of those who screen positive are correctly managed (n=112; primary outcome), the 95%CI for correct management will be +/- 7%.

The sample size also allows us to describe prevalence of individual conditions, and proportion with corrective action taken (secondary outcomes). For example, if 50 of 1000 participants in one age group (5%) screen positive for a given condition, and 75% of these have complete referral, the 95%CI will be 62%-87%, or a 95%CI of 35%-65% if 50% complete referral.

Recruitment 15

Participants recruited in schools will be reached through whole school sessions, as well as parent sessions. Participants recruited in communities will be reached through door to door community outreach as well as community meetings.

Methods: Assignment of interventions (for controlled trials)

Allocation:

Sequence generation 16a NA

Allocation concealment mechanism	16b	NA
Implementation	16c	NA
Blinding (masking)	17a	NA
	17b	NA

Methods: Data collection, management, and analysis

For peer review only

Data collection methods	18a	The data collection and entry processes will be as detailed in Standard Operating Procedures. We will use Open Data Kit (ODK) and the bespoke Y-Check application for electronic capture of quantitative data into custom-designed forms with built-in range, consistency and discrepancy checks. Answers to sensitive questions will be entered by adolescents themselves to reduce social desirability bias. Field supervisors will scan at least 10% of all forms within 48 hours of data collection to check that there are no obvious problems. They will initiate appropriate actions if there are, such as discussing this at the weekly meetings with the field teams, meeting with individual team members, or arranging specific refresher training. In addition, the data management team will continuously monitor the quality of the data through running frequency distributions of the results for each variable in order to identify unlikely patterns and outliers, and these results will be discussed at the weekly field team meetings. Tablets will be password protected and personal identifiers will be stored in an encrypted format. Service use will also be captured in paper logbooks and registers designed for the specific care and prevention services provided. In each case a log will be kept of the number of people reached, products used, tests performed, etc. Logbook data will be entered on to computers on a weekly basis on pre-designed forms.<br><br>De-identified field notes, team debriefing summaries, and outputs from Participatory Action Research (i.e. pictures from mapping, scoring and ranking activities) will be stored electronically in password-protected files. Audio recordings of discussions and interviews will be transcribed verbatim or summarised in detail and then translated (if necessary) into English for analysis by the research team and stored electronically in password-protected files. Each transcript will also have an accompanying summary form capturing details of the data collection and basic demographic details of the interviewee, as well as any pertinent issues related to the data collection session. Verbatim quotations may be included in reports or publications, but will only report the category of participant, their sex and age. De-identified routine health facility data on the uptake of health services by adolescents before, during and after the Y-Check implementation period will be collected. One of the senior social scientists on each of the three country teams will sit in on an average of at least 5% of the interviews, workshops etc, with a higher proportion early in the data collection to ensure quality and to provide feedback to the field researchers. We will also aim that one of the senior social scientists on each of the three country teams will review all qualitative transcripts and summaries within a fortnight of them having been collected so that problems related to how the interviews, participatory workshops, etc have been conducted or recorded/summarized will be identified and the opportunity taken for mentorship to happen.<br><br>For both quantitative and qualitative data, a major method that we will use to ensure data quality is that the data will be reviewed in real time as they are collected and will not be allowed to accumulate un-reviewed. This should allow problems and inconsistencies to be detected and appropriate steps taken to correct errors early in the data collection process.

For peer review only - http://bmjopen.bmj.com/site/about/guidelines.xhtml

	18b	Children in schools will be followed up through continued engagement with the schools. In communities, follow-up will be via phone numbers and addresses provided at the check-up visit. Data on many secondary outcomes will be missing for those lost to follow-up. However, socio-demographic data and primary outcome data will be available for those who are lost to follow-up and can be used to assess potential biases in secondary outcomes due to lost to follow-up.
Data management	19	Data collected off-line on tablet computers will later be synchronised over a local research wi-fi network to the ODK server. Any data transfer over wireless or mobile networks will use Virtual Private Networks or router protected dedicated internet protocol addresses. Data will be fully encrypted to comply with general data protection regulation (GDPR) standards, using a public and private key for encryption and decryption, respectively. All electronic data will be stored in password-protected database systems, with access granted to authorised staff only. When necessary, subsets of the redacted database or other data files may be stored on the PI's or senior staff's laptop to permit analyses during visits or travel. Laptop storage will be encrypted and password-protected to protect data from unauthorised access. Data transferred to LSHTM and/or WHO will be held on Secure Servers utilizing storage systems that provides access controls, integrity verification, encryption, automated daily backup and other functionality to ensure data authenticity and security. While records will not be collected on paper, in some situations (loss of wifi) this might be necessary. Paper records will be stored within the PI or Senior staff's office under lock and key, with access granted only to authorized staff. All data will be stored in multiple secure locations to guard against data loss, and will be stored in date-stamped folders to allow reconstruction of datasets from earlier versions in the unlikely event of a later file becoming corrupted.

view only

For peer review only - http://bmjopen.bmj.com/site/about/guidelines.xhtml

Statistical methods	20a	All primary analyses will be conducted separately by study city; Cape Coast, Chitungwiza and Mwanza. Where comparable, secondary analyses will be conducted with the data from all three cities combined.<br><br>**Validation study**<br>Data will be analysed to calculate the following measures: sensitivity, specificity, positive predictive value, negative predictive value.<br><br>**Programmatic data**<br>Quantitative programmatic data including screening tests results, services delivered, and referrals made and completed will be described according to age, sex, and location.<br><br>**Prospective intervention study**<br>We will follow the STROBE guidelines for the reporting of cohort studies. We will create a flowchart showing the number of communities and schools and the number of participants per community and school at each contact point in the cohort study. We will use descriptive analysis to compare the community-level and school-level characteristics of the study communities and schools.<br><br>The primary outcome is a single proportion which will be presented with a 95% confidence interval for each of the 4 target populations: 10-14 year old male, 10-14 year old female, 15-19 year old male, 15-19 year old female. Secondary outcomes which are measured at a single time point will be presented in a similar way. For outcomes which are measured at two or more time-points, a before-after analysis will be conducted comparing differences in measures between the two time-points. The unit of analysis will be the individual. For clinical outcomes which are measured at two or more time-points, the initial check-up visit (baseline) will give the prevalence of undiagnosed and untreated chronic conditions which will represent the counterfactual. The proportion of undiagnosed and untreated chronic conditions at the 4-month follow-up visit will be formally compared to this counterfactual to estimate the effects of the intervention in improving these clinical outcomes. We will assess health service and client determinants of correct management of conditions at 4 months using multivariable regression. A statistical analysis plan is available.
	20b	All analyses will be disaggregated by age and gender.
	20c	NA

Methods: Monitoring

For peer review only - http://bmjopen.bmj.com/site/about/guidelines.xhtml

Data monitoring	21a	The Data Safety Monitoring Board (DSMB) members will receive and review information on the progress and accruing data of this study. The DSMB should inform the Chair of the PAC if, in their view the results are likely to convince a broad range of clinicians, including those supporting the study and the general clinical community, that, on balance, provision of the Y-Check service is contraindicated for all participants or a particular category of participants, and there was a reasonable expectation that this new evidence would materially influence patient management. The members of the DSMB for this study are:<br>1. Prof Fred Binka, Professor of Clinical Epidemiology, School of Public Health, University of Health and Allied Sciences, Ho, Ghana. Chair, Clinician<br>2. Dr Andrew Abassa, Head of Statistics, MRC/UVRI Uganda Research Unit, Entebbe, Uganda. Statistician<br>3. Prof David Mabey, Professor, London School of Hygiene and Tropical medicine, Clinician<br>4. Dr Nothando Ngwenya, Head of Social Science and Research Ethics, AHRI, South Africa
	21b	The DSMB will be notified in the event of any adverse events. Final decision to terminate the study will rest with the study sponsor.
Harms	22	The DSMB will be notified in the event of any adverse events and make recommendations to the study sponsors.
Auditing	23	NA

Ethics and dissemination

Research ethics approval	24	Ethical clearance has been received from WHO Registration Protocol ID WHO/ERC.0003778 28/08/2023, from London School of Hygiene and Tropical Medicine Approval numbers 26395 and 28312 and from all country national ethics bodies.
Protocol amendments	25	Protocol modifications have been submitted to WHO ethics review committee, LSHTM and national ethics boards and approved by all.

For peer review only

Consent or assent	26a	To respect the autonomy of adolescents the decision of the minor should prevail. As a result, prior to the visit, adolescents will be shared the assent forms. After adolescents have assented, parents/guardians who would like their adolescent to receive the check-up will be asked to provide written parent/guardian consent. On the day of the check-up visit, a verbal confirmation will be requested from the adolescent. This will be the case for all adolescents taking part in Y-Check in school settings. In community settings, we can expect older and possibly emancipated minors to be participants of the Y-Check service. In Ghana and Tanzania where the age of consent to medical and health-related research is 18 years, adolescents who are not deemed emancipated minors will provide completed parental consent forms and provide written assent before proceeding through the check-up visit. In Zimbabwe where the age of consent to medical and health-related research is 16 years, clients aged 16 years and above who attend the check-ups in the community venues will be allowed to provide written consent for themselves. Emancipated minors will be treated as though they were above the nationally-applicable age of consent. The risks and benefits of the Y-Check intervention will be described to participants and their parents/guardians during the consent/assent process. Adolescents receiving parental consent will be informed that their parents will be notified of test results. Y-Check participants will benefit from early detection of health problems, health promotion, and the promotion of beneficial health-seeking behaviours. However, some conditions such as mental health disorders, HIV and sexually transmitted infections (STIs) are associated with stigma and anxiety. The Y-Check team will be trained in good clinical practice (GCP), data protection and confidentiality, and will provide counselling for participants testing positive for any condition. Furthermore, the protocols and procedures for communicating with adolescents and their families will be carefully developed in collaboration with the three Youth Advisory Groups (YAGs)/Community Advisory Board (CAB) and community stakeholders.
	26b	NA

For peer review only

Confidentiality 27

The protocol for sharing the results of the screening tests will vary according to the perceived seriousness of the condition and/or the seriousness of any stigma associated with the condition, and the age of the participant. A final decision on the classification of conditions as being either more or less serious or seriously stigmatized will be taken in collaboration with adolescents and stakeholders during Year 1. More serious and/or seriously stigmatised conditions may include HIV, STIs, pregnancy, drug use, excessive alcohol use, experiencing violence, suspected epilepsy, severe depressive or severe anxiety symptoms and serious musculoskeletal disorders. Less serious and/or less seriously stigmatised conditions may include anaemia, overweight, underweight, pre-hypertension, hypertension, mild depressive or mild anxiety symptoms, myopia or a hearing disorder. Adolescents who are of majority age or deemed emancipated minors will be given their results directly at the time of the screening. Although they will be encouraged to disclose and discuss the results with their parents, unless they think this would put themselves at risk, the Y-Check team will not disclose their results to their parents unless the adolescent asks for a joint meeting with their parents to discuss them. For all other cases: 1) in the event that the adolescent has no positive test results nor clinically important findings – a letter will be sent home with the child disclosing this information and encouraging continued healthy behaviours. The adolescent will also receive a one-page summary telling them what has been checked for and that nothing serious has been found. They will be encouraged to continue good health-related behaviours. The letter will remind them about health and well-being services available at the school, local health facilities and in the community. 2) in the event that the adolescent is not diagnosed with any condition that requires follow-up or referral (see below) but is diagnosed with a condition that is relatively minor (such as being overweight but not obese) – a letter will be sent home with the child that discloses the results and provides information that promotes and enables self-management, and encourages assistance from their parents. 3) in the event that the adolescent is not diagnosed with any condition that requires referral (see below) but is diagnosed with a condition that is relatively minor but needs follow-up (such as moderate anaemia) – a letter will be sent home with the child that discloses the results, provides information to the parent, and offers the opportunity for the parent to make an appointment with a member of the Y-Check team for further advice regarding the follow-up that may be required (eg. reassessment of the adolescent´s haemoglobin concentration after the three months of iron and folate treatment provided by Y-Check has been completed). 4) in the event that the adolescent is not diagnosed with any condition that requires referral and is stigmatizing (see below) but is diagnosed with a condition that requires referral but is not stigmatizing (such as myopia) – a letter will be sent home with the child that discloses the results and provides information to the parent. The parent will be offered the opportunity to make an appointment to rmeet with a member of the Y-Check team for further advice regarding the condition and the referral. If the parent does not take up the offer of an appointment, the study team will consult with the adolescent, and – if the adolescent gives their permission - school, health or social care staff before deciding on next steps. Potential action would include contacting the parent by phone or through a home visit. 5) in the event that the adolescent is diagnosed with a condition that is considered to be more serious and/or more seriously stigmatizing (such as HIV or epilepsy) – a letter will be sent home with the child that does not disclose results. Parents will be invited and encouraged to make an appointment with the Y-Check nurse/clinician or counsellor to discuss the adolescent's health. The nurse/clinician or counsellor would then meet with the adolescent and the parent together and would explain the condition or suspected condition and what is being recommended. They will ask for the parent´s support to manage the condition and answer any questions and give advice/support as needed. If the parent does not take up the offer of an appointment, the study team will consult with the adolescent, and – if the adolescent gives their permission - school, health or social care staff before deciding on next steps. Potential action would include contacting the parent by phone or through a home visit, and, in emergency situations only, referring the young person to health/social services without the support of the parent.

For Y-Check in community settings, the adolescent will receive the letter directly and will be encouraged to share it with their parents/guardians. For Y-Check in school settings, the letter will be sent from Y-Check via the school to the adolescent´s parents. All such letters to parents/guardians will be in sealed envelopes addressed to the "Private and Confidential: to the Parent/Guardian of <name of adolescent. In this study, SRH services are only provided in community settings. In Zimbabwe, community services will be provided to 16-19 year-olds, and adolescents who are 16+ in Zimbabwe are able to consent themselves, and therefore no parental disclosure is required, though we will suggest that adolescents inform their parents if they think that they will be able to provide support. In Ghana and Tanzania, older adolescents (16y+) will be able to access Y-Check in community settings. Unless they are deemed emancipated minors (section 7.5), they will require parental consent. The consent and assent forms note that test results will be disclosed to the parent.

We feel that parents have an important role to play in supporting legal minors, especially after diagnosis of highly stigmatizing conditions, including supporting them emotionally, connecting them with services, treatment or follow-up care. As a result, rather than apply a universal rule for this group, we propose development of a process that respects the best interest of the adolescent, that will enable the clinician to determine the benefits and risks of parental disclosure on a case-by-case basis. We will work during the first part of this study to discuss this with national and international clinicians and researchers as well as global and local ethics boards to determine what is the right course of action.

Victims of rape/sexual abuse
In the event that the Y-Check team discovers a case of rape or sexual abuse amongst the participants, the matter shall be referred to the Social Services department. Participants will be told of this legal requirement during the consent procedure so they can decide whether or not they wish to report any such events.

For peer review only

For peer review only - http://bmjopen.bmj.com/site/about/guidelines.xhtml

Declaration of interests	28	The principal investigators have no completing interests.
Access to data	29	All PIs and co-investigators will have access to the data. Once the study is completed, all data will be placed into an open access repository. Data will be deidentified and anonymised to ensure confidentiality and participant privacy
Ancillary and post-trial care	30	NA
Dissemination policy	31a	A publications policy has been developed. Results will be published in at least 3 country specific peer reviewed journal publications and one multicountry publication. There will also be videos, briefs, and webinars to disseminate results.
	31b	Topics suggested for presentation or publication will be circulated to the PIs of the management team, with an abstract, proposed authorship and proposed journal. A writing committee will be formed as described in the publications policy. Disputes regarding authorship will be settled as per the publication policy, and ultimately by the Lead PI if required.
	31c	All data will be placed into an open access repository. Data will be deidentified and anonymised to ensure confidentiality and participant privacy.
**Appendices**		
Informed consent materials	32	Attached in the submission to Ethics Review Committee.
Biological specimens	33	The laboratory tests will be conducted by a trained laboratory technician or laboratory assistant and will include:<br>• Anaemia, using haemoglobin measurement<br>• HIV testing for older adolescents using a HIV oral mucosal transudate test with confirmatory blood testing using Rapid Diagnostic Tests<br>• STI testing for the older adolescents using GeneXpert for Chlamydia trachomatis (CT) and Neisseria gonorrhoeae (NG) and a lateral flow assay for trichomonas vaginalis (TV).<br>• In the two cities where the prevalence of malaria is expected to be high (Mwanza and Cape Coast), all participants will be tested for malaria parasites using the rapid diagnostic test that is recommended by the national malaria control programme.<br>• In the two cities where the prevalence of schistosomiasis is thought to be high (Mwanza and Cape Coast), all participants will be asked to provide both a urine specimen that be tested for *Schistosoma haemotobium* and *Schistosoma mansoni*.<br>No samples will be stored. All samples will be destroyed after testing is completed.

For peer review only

*It is strongly recommended that this checklist be read in conjunction with the SPIRIT 2013 Explanation & Elaboration for important clarification on the items. Amendments to the protocol should be tracked and dated. The SPIRIT checklist is copyrighted by the SPIRIT Group under the Creative Commons "Attribution-NonCommercial-NoDerivs 3.0 Unported" license.

For peer review only
